# Synthesis of Urea‐Containing Derivatives and their Application as Potential Anti‐Methicillin‐Resistant Staphylococcus Aureus Agents

**DOI:** 10.1002/cmdc.202500521

**Published:** 2025-09-05

**Authors:** Jorge A. González‐Cruz, Gerardo González‐Gallardo, J. Ricardo Pérez‐Velázquez, Carlos D. García‐Mejía, José Manuel Guevara‐Vela, Jesús A. Oria‐Hernández, Adriana Castillo‐Villanueva, Tomás Rinza‐Rocha, Eduardo Hernández‐Vázquez

**Affiliations:** ^1^ Departamento de Química Orgánica Instituto de Química Universidad Nacional Autónoma de México (UNAM) Ciudad de México 04510 México; ^2^ Laboratorio de Bioquímica‐Genética Instituto Nacional de Pediatría Secretaría de Salud Ciudad de México Ciudad de México México; ^3^ School of Engineering and Physical Sciences Heriot‐Watt University Edinburgh EH14 4AS Scotland UK; ^4^ Departamento de Fisicoquímica Instituto de Química Universidad Nacional Autónoma de México (UNAM) Ciudad de México México

**Keywords:** antimicrobial resistance, FabI inhibitors, molecular docking, methicillin‐resistant *Staphylococcus aureus*, urea‐derivatives

## Abstract

We describe the synthesis and activity against methicillin‐resistant *Staphylococcus aureus* (MRSA) of a collection of urea‐containing amides. The approach considered the ureido group as a bioisoster of known FabI inhibitors. NMR characterization and density functional theory studies demonstrated the presence of *s‐cis* and *s*‐*trans* rotamers in the *N*‐benzyl examples (series **2**). Preliminary screening showed the ability of series **1** and **3** (*N*‐aryl and *N*‐arilpiperidone derivatives, respectively) to inhibit the bacterial growth of two MRSA strains (a clinical isolate and ATCC 33591). Compound **3b** inhibited 50% of the clinical strain and 34% of the ATCC. Subsequent biological assays let us determine the IC_50_ values of the most active ureas in both strains, standing out compounds **1a** (45.8 ± 2.3 μM) and **3b** (43.6 ± 2.0 μM). Finally, molecular docking suggests FabI as a possible molecular target for the designed compounds.

## Graphical abstract

A series of propylureido amides against methicillin‐resistant Staphylococcus aureus (MRSA) activity was prepared. The design involved replacing other hydrogen bond donors reported in FabI inhibitors with a urea moiety, while maintaining other pharmacophoric features. Compounds showed moderate activity against two MRSA strains, thus positioning them as a novel starting point for the design of more potent analogs.

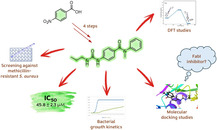

## 1 Introduction

The isolation of penicillin by A. Fleming in 1928,^[^ ^1^ ^]^ along with the synthesis of arsphenamine by the group of Paul Elrich some years before,^[^ ^2^ ^]^ positions as one of the most remarkable discoveries against bacterial pathogens. Eventually, several synthetic and natural antimicrobials joined the catalog of molecules for treating bacterial infections. Notwithstanding, the evolution of pathogens, accelerated by anthropogenic causes (mainly the misuse of antimicrobials), has depleted the bioactivity of such compounds and led to “the antimicrobial resistance” era. Accordingly, new strategies against pathogens must focus on unexplored biosynthetic and essential pathways (for example, cell wall, fatty acid, and nucleic acid biosynthesis) to retard the development of resistance.^[^ ^3^ ^]^ For example, current approaches include: targeting enzymes involved in cell wall recycling,^[^ ^4^ ^,^ ^5^ ^]^ attacking virulence and pathogenesis,^[^ ^6^ ^]^ and quenching quorum sensing.^[^ ^7^ ^]^

In recent years, investigating the fatty acid biosynthesis in bacteria resulted in the discovery of novel substances with antimicrobial activity, such as afabicin^[^ ^8^ ^]^ and nilofabicin.^[^ ^9^ ^]^ In bacteria, the type II fatty acid synthesis pathway (FASII) consists of single proteins (named Fab enzymes), which elongate malonyl‐ACP and construct the unsaturated acyl fragments necessary to assemble lipoic acid, phospholipids, and lipopolysaccharide.^[^ ^10^ ^]^ Recently, our efforts against multiresistant bacteria rely on Enoyl‐ACP reductase inhibition (also called FabI), the last participant during the long‐chain fatty acid elongation cycle.^[^ ^11^ ^]^ Although other Fab enzymes have reductase activity (such as FabK and FabL),^[^ ^12^ ^]^ FabI is essential for pathogens such as *Staphylococcus aureus*,^[^ ^13^ ^]^ thus bringing a narrow spectrum therapy and minimizing the development of resistance.

**Figure 1:**
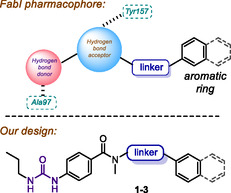
Main structural features found in fabI inhibitors. Below, the design of the urea derivatives **1–3** is shown.

After comparing the structures of synthetic inhibitors, we can deduce some recurrent features, as shown in **Figure** **1**. First, a hydrogen bond donor is necessary to interact with Ala97 within the FabI catalytic cavity. In addition, a hydrogen bond acceptor (carbonyl or azole ring) stabilizes the ligand–receptor complex by interacting with Tyr157. Finally, an aromatic ring (or bicyclic aromatic ring) at the end of the structure interacts with a hydrophobic cavity of the reductase.

In the case of the hydrogen bond donor, several functionalities decorate the inhibitors, like phenols,^[^ ^14^ ^]^ amines,^[^ ^15^ ^,^ ^16^ ^]^ or nitrogen‐containing heterocycles;^[^ ^17^ ^]^ nonetheless, the activity of urea derivatives has not been evaluated until now. Therefore, we envisioned that the urea moiety could bind to Ala97 through two hydrogen bonds in a similar fashion to Afabicin dephosphono.^[^ ^18^ ^]^ Herein, our goal covered the synthesis of propylureido‐containing amides that fulfill the pharmacophoric features found in Figure 1, varying the linker and aromatic moieties. We tested the prepared compounds against two strains of methicillin‐resistant *S. aureus* (MRSA); the results are described below.

## 2 Results and Discussion

### 2.1 Chemistry and Characterization

**Figure 2:**
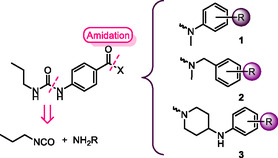
Design and retrosynthetic analysis of urea‐derivatives **1–3**.

According to Figure 1, we started investigating the potential antimicrobial activity of propyl ureas (due to the commercial availability of propyl isocyanate) by adding different linkers and aromatic rings at the final region of the molecule. Thus, series **1** and **2** included *N*‐aryl and *N*‐benzyl substituents, respectively, while series **3** included *N*‐arylpiperidines (**Figure** **2**). Those fragments can be incorporated through an amidation reaction, whereas the ureido portion may be installed by reacting the corresponding aniline with propyl isocyanate.

**Scheme 1:**
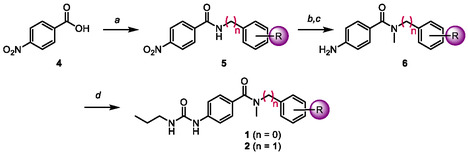
Synthesis of *N*‐aryl and *N*‐benzylbenzamides. Reagents and conditions: a) SOCl_2_, DMF, DCM, 0 °C, then corresponding aniline/benzylamine, TEA, DCM, 0 °C to r.t. 3–5 h; b) NaH, THF, 0 °C, then MeI, r.t., 5–8 h; c) Fe°, HCl, EtOH–H_2_O, reflux, 2–3 h; d) propyl isocyanate, MeCN, 45 °C, 24–48 h.

We prepared series **1** and **2** following a similar synthetic procedure (**Scheme** **1**). In the case of series **2**, our first idea involved the amide formation between 4‐nitrobenzoic acid and the *N*‐methylated benzylamines. Thus, we envisioned the synthesis of the alkylated benzylamines via reductive amination (using the corresponding benzaldehydes and methylamine), but the main isolated product corresponded to the symmetric tertiary amine. We obtained similar findings when using the corresponding benzylamine and paraformaldehyde. Therefore, the methylation of the amide in later synthetic stages replaced our original idea.

According to Scheme 1, 4‐nitrobenzoic acid (**4**) reacted with thionyl chloride (SOCl_2_) to generate the corresponding acyl chloride, which reacted with the corresponding amines (anilines for series **1** and benzylamines for series **2**). Then, methylation of amide **5** afforded the secondary *N*‐methyl amides, starting materials for the nitro group reduction using Bechamp conditions. Finally, treating aniline **6** with propyl isocyanate achieved the desired urea‐containing amides **1** and **2** (acetonitrile as solvent and heating at 50 °C resulted in the best conditions for the final addition step).

**Scheme 2:**
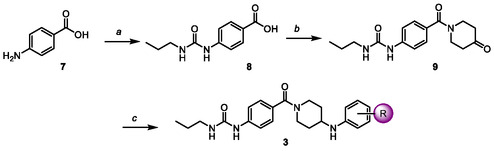
Synthesis of *N*‐acylpiperidines **3**. Reagents and conditions: a) propyl isocyanate, MeCN, 45 °C, 24 h; b) 4‐piperidone monohydrate monohydrochloride, DCC, 4‐DMAP, THF; c) corresponding aniline, AcOH, EtOH, Na_2_SO_4_, 12 h, 50 °C, then NaBH_3_CN, 2 h.

In the case of *N*‐arylpiperidines (**3**), the methodology involved the synthesis of the key ketone **9**, which served as the starting material for the reductive amination (**Scheme** **2**). Accordingly, treating **7** with propyl isocyanate gave the urea **8**, which was coupled with 4‐piperidone to isolate the ketone **9**. The last step consisted of mixing **9** and the corresponding aromatic amine in an acidic medium to give the expected imine, which was reduced in situ with sodium cyanoborohydride.

**Table** **1** shows the molecules belonging to each series. We selected substituents attached to the aromatic ring with different electronic properties, like electron‐donating (methyl and methoxy) and electron‐withdrawing groups (chlorine). The use of bicyclic rings seems relevant to the affinity toward FabI,^[^ ^19^ ^,^ ^20^ ^]^ so we decided to prepare some urea‐derivatives containing naphthyl (**1e, 1f**), and 2,3‐dihydrobenzo[b][1,4]dioxane (**1g**). The single‐crystal X‐ray analysis unambiguously confirmed the structure of the *N*‐aryl derivative **1a** (CCDC number = 2,430,359); besides, it is curious that the amide adopted a *s*‐*cis* conformation.

**Table 1:** Urea‐derivatives prepared. The 3 subsets are shown.

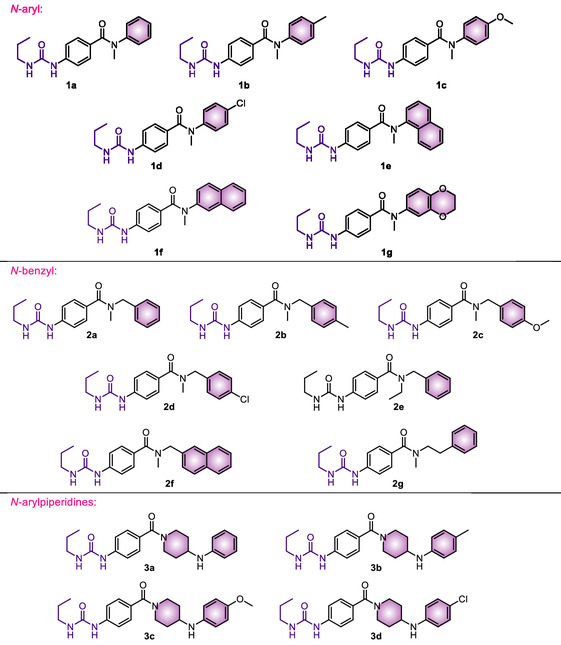

**Figure 3:**
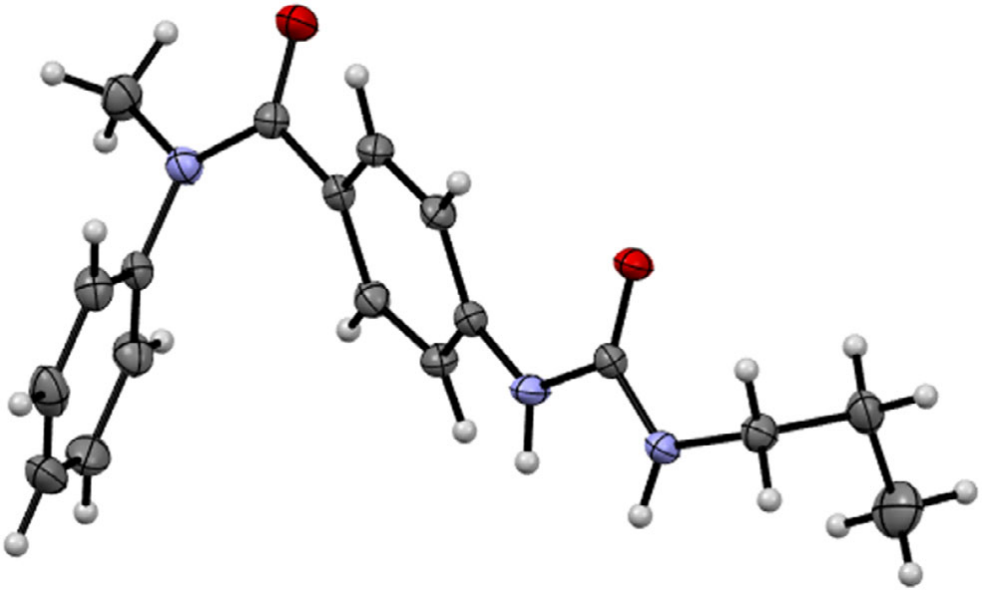
Single crystal X‐ray structure of **1a**. An unusual *s‐cis* conformation is preferred.

Spectroscopic (NMR and IR) and spectrometric techniques (mass spectrometry) characterized and confirmed the structures of the subsets **1**–**3**. We noticed that the ^1^H‐NMR of series **2** showed some aliphatic hydrogens as broad signals, attributed to the presence of conformers; those signals displayed the expected multiplicity when heated to 80 °C (**Figure** **3** shows the NMR comparison of **2e** as an example). The conformer shifting mainly impacted the amide region; thus, ureas of series **2** exist as a mixture of *s*‐*cis* and *s*‐*trans* isomers. Besides, the interchangeable conformers also affected the aliphatic carbons attached to the amide, and a delay of 4 s and more than 1000 scans were necessary for their observation. However, the subset **1** lacks this phenomenon, and the NMR spectra unveiled the expected multiplicity.

**Figure 4:**
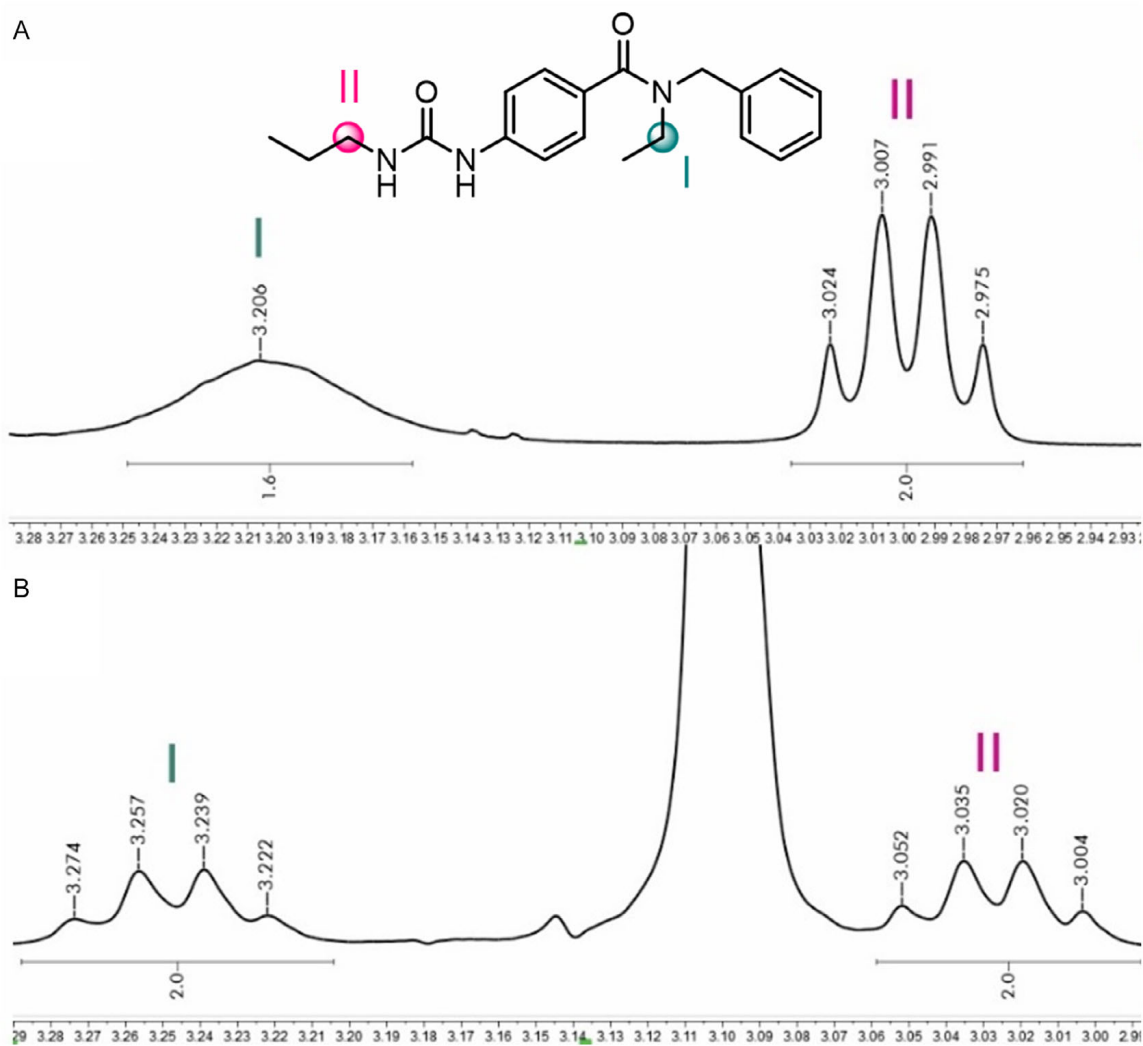
^1^H‐NMR expansion of **2e**: A) 25 °C; B) 80 °C. Heating to 80 °C (B) led to the visualization of the quartet multiplicity of hydrogens in **I**, while the NMR spectra at room temperature appeared as a broad signal (A).

**Figure 5:**
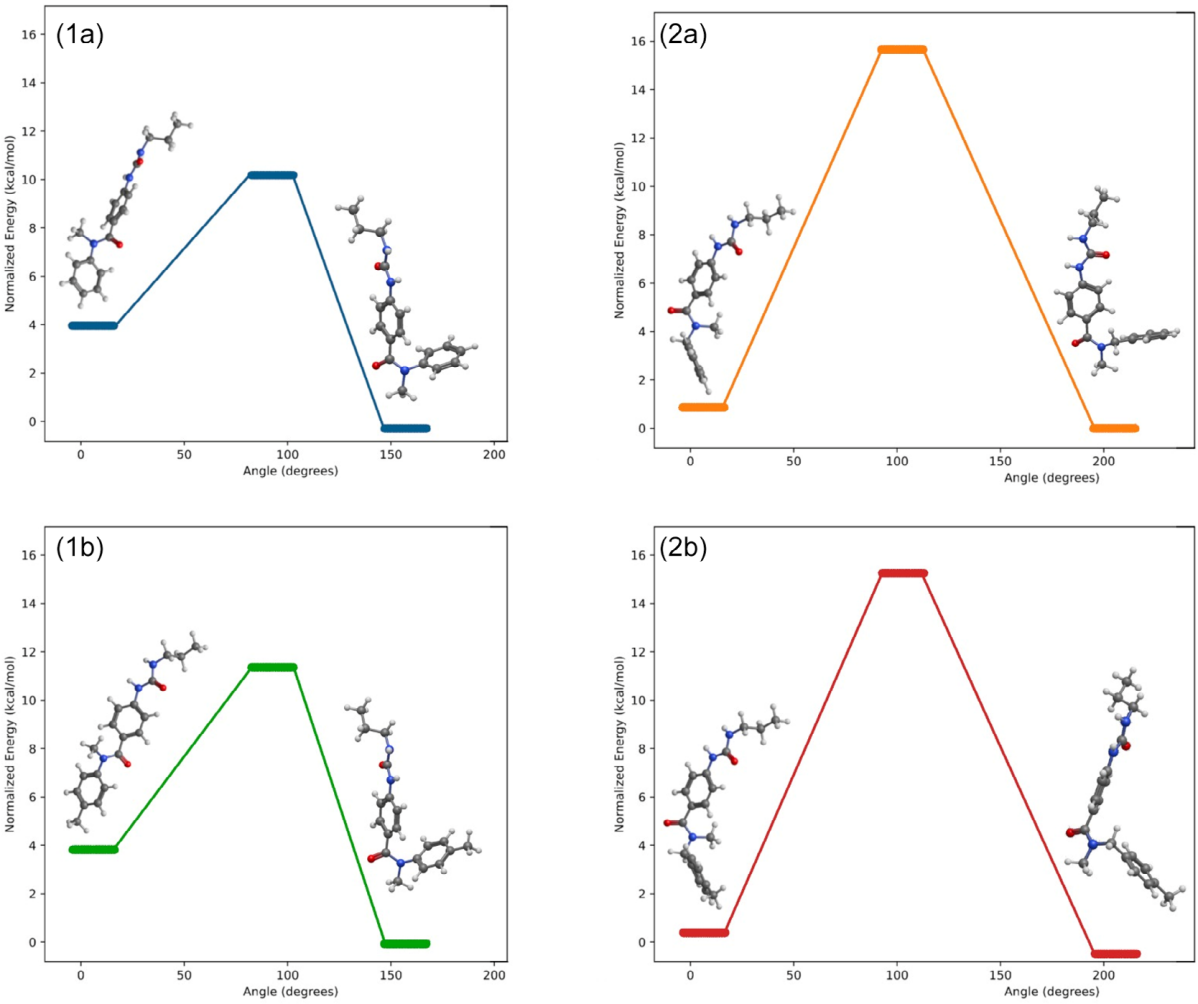
Conformational analysis of **1a**, **1b**, **2a**, and **2b**. The interconversion of benzylic conformers requires more energy independently of the substituent.

To propose a plausible explanation for this behavior, we conducted a computational study using density functional theory (DFT) calculations. The potential energy surface scans were performed by systematically varying the dihedral angles of the amide bond while optimizing all other degrees of freedom until both conformers were reached (*s*‐*cis* and *s*‐*trans*, **Figure** **4**). The analysis included **1a** and **1b** (as phenyl derivatives), while **2a** and **2b** acted as benzyl‐containing ureas. The *s*‐*cis* is more stable than the *s‐trans* conformer in all the minimized structures, while the X‐ray structure confirmed the theoretical finding. We propose that an intramolecular *π*–*π* interaction involving the two phenyl rings stabilizes the *s‐cis* conformation (See **Figure** 4 and **5**);^[^ ^21^ ^]^ besides, the phenyl or benzyl substituent offers higher steric hindrance than the methyl group, thus avoiding the *s‐trans* conformation.

Furthermore, during the amidation reaction, both isomers can be obtained. The energy barrier in the case of series **1** is low enough to allow the quick interconversion of rotamers (4–12 kcal mol^−^^1^). Instead, for the benzylic ureas, both rotamers have similar energies, so we expect an equal concentration of both conformers. However, the high energy barrier (15–16 kcal mol^−^^1^) avoids the interconversion. Therefore, after urea formation, both conformers remain, and the aliphatic hydrogens near the amide bond in the NMR spectrum at room temperature appear as broad signals. When heated to 80 °C, both isomers reach equilibrium, and the aliphatic hydrogens become well‐defined and sharpened signals. It is noteworthy that the substituent attached to the phenyl ring seems irrelevant in both series.

### 2.2 Activity against MRSA

**Figure 6:**
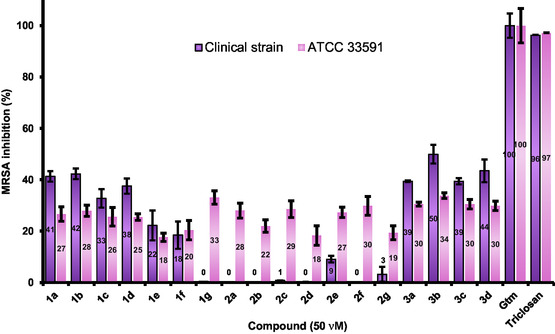
Percentage of MRSA inhibition at 50 μM for ureas, gentamicin (Gtm), and triclosan. The data represent the mean ± SEM from three different experiments.

The series’ design considered the reductase FabI as the receptor, so the experiments focused on the pathogen *Staphylococcus aureus.* We cultivated two strains of MRSA: an ATCC (**33591**) and a clinically isolated strain (resistant to methicillin and oxacillin). Gentamicin (Gtm) nd triclosan (a well‐known FabI inhibitor)^[^ ^22^ ^]^ were used as positive controls. **Figure** **6** shows the MRSA inhibition at a concentration of 50 μM. Although both strains correspond to the same gram‐positive pathogen, we detected notable differences in their susceptibility. Regarding the clinical strain, benzylic ureas (**2a–g**) lack activity, whereas the remaining series showed antimicrobial effects, especially when a methyl group decorated the aromatic ring (42 and 50% inhibition for **1b** and **3b**, respectively). Substitution at the phenyl ring had little impact on activity: both electron‐donating (methyl and methoxy) or electron‐withdrawing groups (chlorine) reduced bacterial growth in a similar quantity. Contrary to other FabI inhibitors, bicyclic moiety drastically reduced the antimicrobial effect, as shown in examples **1e**, **1f** (naphthyl), and **1g** (1,4‐benzodioxane). To our surprise, the piperidine‐derived ureas (**3a–d**) proved to be the most effective subseries, with inhibitions ranging from 39 to 50%. Some previously published synthetic antimicrobials contain an internal piperidine (or the diaza analog piperazine)^[^ ^14^ ^,^ ^23^ ^,^ ^24^ ^]^ in their structure, thus series **3** may be explored in future investigations.

All compounds inhibited the growth of MRSA ATCC 33591;^[^ ^25^ ^,^ ^26^ ^]^ however, preliminary screening revealed a different relationship: the phenyl series **1** displayed less activity than in the clinical isolate (except for **1g**, which showed 33% inhibition); however, the inhibition improved when a methylene group was added (series **2**). Again, a methyl group at the aromatic fragment favored the antimicrobial effect (**1b** and **3b**). Analogs **3a–d** resulted in the most active subset, where compound **3b** stands out as the most interesting analog in both MRSA strains.

**Figure 7:**
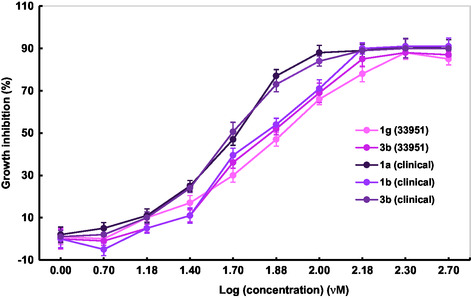
Concentration‐response curves for the determination of the IC_50_ of the most active ureas (**1a**, **1b**, **1g**, and **3b**).

After the preliminary screening, we proceeded to calculate the IC_50_ of the most active analogs. As observed in **Figure** **7**, all tested compounds exhibited a concentration–response curve, with increasing inhibition at higher concentrations. In accordance with Figure 6, the clinical strain was more susceptible than the ATCC strain. The unsubstituted analog **1a** became the most active analog (IC_50_ = 45.8 ± 2.3 μM), followed by the methylated **1b** (60.2 ± 4.5 μM) and the piperidine **3b** (62.0 ± 3.9 μM). In the case of the two tested urea derivatives against MRSA ATCC, **3b** had higher activity than the bicyclic **1g** (43.6 ± 2.0 and 69.1 ± 1.8 μM, respectively).

**Figure 8:**
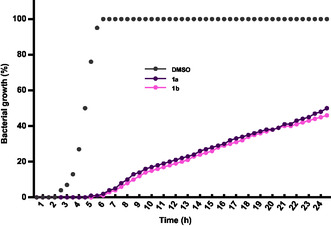
Effect of ureas **1a** and **1b** on the time‐kill curves of MRSA (clinical). The IC_50_ was used in both cases.

Having the IC_50_ values, we analyzed the bacterial growth kinetics after treatment with the phenyl derivatives **1a** and **1b**. Compared to the vehicle (DMSO), both compounds delayed the log phase until 7 h and reduced MRSA growth to less than 60% (even at 24 h after administration). Furthermore, while the negative control reached the stationary phase at 6 h, we did not identify that phase after treatment with the ureas (**Figure** **8**).

Finally, both MRSA ATCC 33951 and the clinical strain secrete biofilm,^[^ ^27^ ^]^ a quorum‐sensing mediated virulence factor.^[^ ^28^ ^]^ Biofilms limit antimicrobial penetration and confer resistance to them (up to 1000 times more resistant compared to planktonic cells).^[^ ^29^ ^]^ Therefore, we employed the crystal violet assay to investigate whether the most active compounds reduce biofilm in MRSA strains. According to **Table** **2**, only **1d** and piperidone **3b** reduced the biofilm by more than 20%. Though the activity is poor, the idea of having compounds with antimicrobial and antibiofilm properties makes **1d** and **3b** interesting molecules for future investigations.

**Table 2:** Activity of selected ureas against MRSA biofilms at 50 μM. The table represents the mean ± SEM.

Compound	Biofilm reduction [%]	
	ATCC 33951	Clinical strain
**1a**	17.3 ± 3.6	7.1 ± 5.0
**1d**	31.7 ± 4.8	21.4 ± 4.1
**1h**	13.5 ± 2.7	12.0 ± 3.0
**2a**	10.2 ± 2.8	3.8 ± 3.4
**2b**	12.8 ± 5.7	0.1 ± 4.6
**2c**	8.9 ± 4.4	10.7 ± 7.7
**3b**	27.0 ± 3.1	21.3 ± 4.1

The series had a less antimicrobial effect than the FabI inhibitor triclosan (IC_50_ of 104 ± 2.71 nM for ATCC and 115 ± 3.50 nM for the clinical strain); therefore, we can deduce that urea has a weaker interaction with Ala 97 than other groups, such as naphthyridone or amino (found in afabicin dephosphono and nilofabicin, respectively). Nonetheless, we hope these series can serve as a starting point for the design of new anti‐MRSA agents, specifically by adding more or longer aliphatic chains to series **1**, or enhancing the diversity of substituents in series **3**.

### 2.3 Molecular Docking Studies

**Figure 9:**
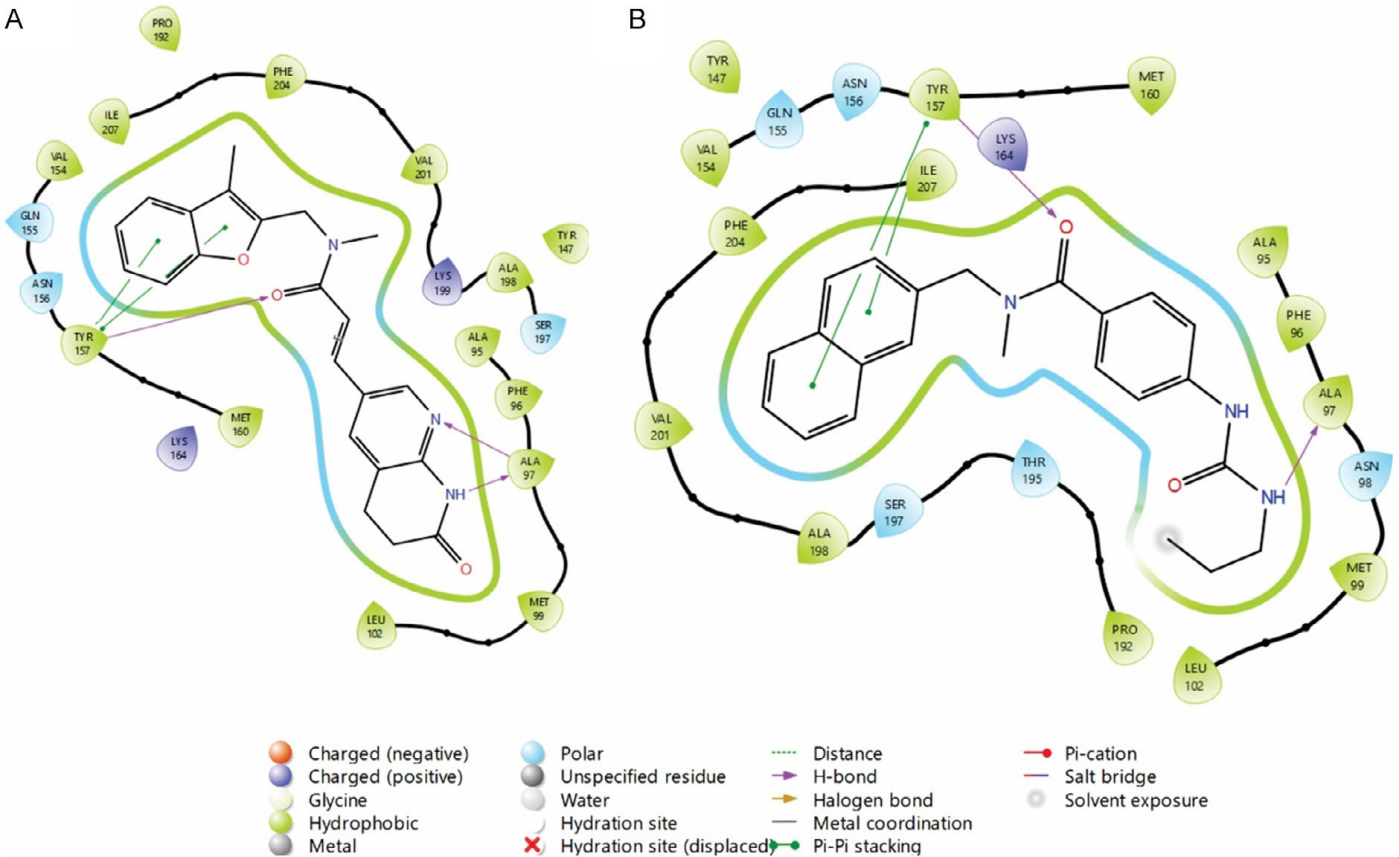
Two–dimensional interaction plot of afabicin dephosphono A) and **2f** B) inside the fabI catalytic domain (PDB: 4FS3).

We docked the series and afabicin dephosphono into the catalytic domain of *Staphylococcus aureus* FabI (PDB: 4FS3). The GLIDE program (Extra Precision Protocol) served as the methodology for the computational study. Accordingly, compounds showed negative docking scores, indicating a favorable interaction with the FabI catalytic domain (**Table** **3**). Afabicin displayed the best affinity (−11.35), attributable to the hydrogen bonds with Tyr157 and Ala97 and the hydrophobic interactions with Tyr157 (**Figure** **9A**). Regarding our work, the benzylic **2** subset displayed optimal scores (scores ranging from −8.81 to −10.94), being the analogs with a bicyclic ring (naphthalene and benzodioxolane) being the most affinal to the FabI catalytic domain. The binding pose of **2f** (docking score of −10.94) shares a similar pose to afabicin: the carbonyl of the amide interacts with Tyr157 (including hydrophobic and hydrogen bond), and the urea forms a hydrogen bond with Ala97 (Figure 9). We propose that the existence of two uninterchangeable isomers in series **2** may affect the bactericidal activity. While the *s‐trans* conformation is the bioactive (see Figure 9), the *s‐cis* may exhibit unfavorable interactions with the FabI catalytic domain. This inequality is frequently observed in medicinal chemistry and establishes a threat during drug discovery.^[^ ^30^ ^]^

**Table 3:** *In silico* affinity of ureido‐containing amides.

Compound	Docking score	Compound	Docking score
**1a**	−7.28	**2d**	−9.12
**1b**	−6.71	**2e**	−8.81
**1c**	−5.46	**2f**	−10.94
**1d**	−7.02	**2g**	−9.21
**1e**	−9.23	**3a**	−9.69
**1f**	−5.00	**3b**	−7.70
**1g**	−7.81	**3c**	−3.96
**2a**	−9.21	**3d**	−4.16
**2b**	−9.31	**Afabicin** **dephosphono**	−11.35
**2c**	−8.90		

Molecular docking studies suggest that propyl ureido derivatives are plausible inhibitors of *S. aureus* FabI. Furthermore, favorable SwissADME predictions^[^ ^31^ ^]^ and no violations of the Lipinski suggestions (Table S2) reinforce their potential as plausible new antibacterial molecular scaffolds. At this stage, however, bacteriological experiments indicated moderate to low activity against MRSA strains. The discrepancy can be related to intrinsic molecular properties (for example, low solubility in bicyclic derivatives) or bacterial factors, such as efflux pumps (a mechanism that eliminates toxic substances from the cytoplasm).^[^ ^32^ ^]^ Moreover, considering that the antimicrobial effect of the tested ureas starts at 7 h after incubation and the series had a reduced antibiofilm activity, we suggest that the pathogens constructed biofilm, thus limiting penetration or even metabolizing the compounds. In the case of positive controls (gentamicin and triclosan), the bactericidal activity is so fast that the secretion of exopolysaccharide, the main component of biofilm, is limited. Notwithstanding, the outcomes shown herein provide a foundation for subsequent design, synthesis, and biological testing cycles focused on enhancing the pharmacological potential of these urea‐containing amides.

## 3 Conclusions

As an effort to combat MRSA, we prepared a series of propyl‐ureido derivatives aimed to inhibit FabI, a vital enzyme for this pathogen. The design involved the bioisosteric replacement of previously reported hydrogen bond donors/acceptors with Ala97. After spectroscopic characterization, we detected the presence of rotamers in examples of series **2**, and DFT studies confirmed a high energetic barrier required for their interconversion. Compounds were tested against two MRSA strains, displaying different susceptibility. Series **3** (piperidines) resulted in the most active group (reaching, in some cases, 50% inhibition against the clinical isolate). Concerning the effect of substituents, methylation gave better results than electron‐withdrawing groups. Molecular docking confirmed the similar binding mode of the ureido‐amides and afabicin inside the FabI catalytic domain, in which the urea functionality interacts with Ala97 as predicted during the design. The piperidine **3b** becomes the most interesting derivative based on its activity against both strains. Altogether, the results warrant further investigation of new ureido‐derivatives intended to improve anti‐MRSA activity.

## 4 Experimental Section

4.1

4.1.1

##### General Considerations

Reagents and solvents were purchased from Merck and Química Rique and were used without purification. The advance of the reactions was monitored by TLC using the appropriate eluent (generally a hexane–ethyl acetate mixture). NMR spectra were recorded with a Jeol Eclipse (300 MHZ) or on a Jeol JNM‐ECZ400S 400 MHz spectrometer using CDCl_3_ and DMSO‐d6. 700 MHZ spectra were recorded on a Bruker Ascend 700. Mass spectra (MS) and High‐resolution mass spectra (HRMS) were measured using a Jeol JMS‐T100LC the AccuTOF mass spectrometer with direct analysis in real time (DART) ionization technique, or the MStation JMS‐700 (electronic impact). Infrared (IR) spectra were collected with an FTIR NICOLET IS‐50 equipped using the attenuated total reflection) technique. Plates were read on a BioTek Cytation 5 Cell Imaging Multi‐Mode Reader. Compound characterization data (including full ^1^H‐NMR, ^13^C‐NMR, and HRMS) are detailed in the supporting file.

##### Synthesis of 4‐aminobenzamides 6a–h

The corresponding 4‐nitrobenzamide **5a–h** (0.42 mmol) was dissolved in anhydrous THF (2.0 mL) and cooled to 0 °C. Later, sodium hydride in mineral oil at 60% (0.71 mmol) was added in one portion. The mixture was stirred for 15 min. After this, the solution turned red, and methyl iodide (0.48 mmol) was added. The methylation was allowed to react for 8–10 h (the *N*‐methylamides had less Rf than the starting material in TLC). The solvent was removed under vacuum distillation, and the remnant was redissolved in ethyl acetate (AcOEt) and washed with an aqueous solution of NaHCO_3_ (10%). The organic layer was dried over sodium sulfate and evaporated under reduced pressure.

The unpurified *N*‐methylamides and iron (1.15 mmol) were added to a round‐bottom flask and suspended in a mixture of EOH–Water (2:1, 67 mM). A catalytic amount of concentrated HCl was added, and the reduction was heated to reflux for 3–6 h until the completion of the starting material. The reaction was cooled to room temperature and evaporated under reduced pressure. The crude was resuspended in an aqueous solution of NaOH (0.1 M) and extracted with AcOEt (3  × 10 mL). The organic phase was dried over sodium sulfate, evaporated, and purified by flash column chromatography.

##### 4‐amino‐*N*‐methyl‐*N*‐phenylbenzamide (6a)

Purified by flash column chromatography (Hex‐AcOEt, 6:4). Yellowish solid in 74% yield. ^**1**^ **H‐NMR** (400 MHz, CDCl_3_) *δ*: 7.22–7.18 (m, 2H), 7.13–7.08 (m, 3H), 7.02–7.00 (AA´BB´, 2H), 6.36–6.33 (AA´BB´, 2H), 4.64 (br s, 2H, NH_2_), 3.42 (s, 3H, CH_3_N); ^**13**^ **C‐NMR** (100 MHz, CDCl_3_) *δ*: 170.8, 148.2, 145.8, 131.1, 129.2, 126.8, 126.1, 125.0, 113.6, 38.7; **MS** (DART+) m/z [M + H]^+^: 227 m/z; **HRMS** m/z calcd for ^12^C_14_^1^H_15_^14^N_2_^16^O_1_ [M + H]^+^, 227.11844; found 227.11840.

##### 4‐amino‐*N*‐methyl‐*N*‐(*p*‐tolyl)benzamide (6b)

Purified by flash column chromatography (Hex‐AcOEt, 6:4). Yellowish solid in 47% yield. ^**1**^ **H‐NMR** (400 MHz, CDCl_3_) *δ*: 7.14–7.11 (AA´BB´, 2H), 7.02–7.00 (AA´BB´, 2H), 6.92–6.90 (AA´BB´, 2H), 6.40–6.38 (AA´BB´, 2H), 3.80 (br s, 2H, NH_2_), 3.43 (s, 3H, CH_3_N), 2.28 (s, 3H, CH_3_Ph); ^**13**^ **C‐NMR** (100 MHz, CDCl_3_) *δ*: 170.0, 148.0, 143.4, 135.9, 131.1, 129.8, 126.7, 125.5, 113.7, 38.8, 21.0; **MS** (DART+) m/z [M + H]^+^: 241 m/z; **HRMS** m/z calcd for ^12^C_15_^1^H_17_^14^N_2_^16^O_1_ [M + H]^+^, 241.13409; found 241.13421.

##### 4‐amino‐*N*‐(4‐methoxyphenyl)‐*N*‐methylbenzamide (6c)

Purified by flash column chromatography (Hex‐AcOEt, 45:55). Yellowish solid in 39% yield. ^**1**^ **H‐NMR** (400 MHz, CDCl_3_) *δ*: 7.11–7.09 (AA´BB´, 2H), 6.94–6.92 (AA´BB´, 2H), 6.74–6.72 (AA´BB´, 2H), 6.39–6.37 (AA´BB´, 2H), 3.85 (br s, 2H, NH_2_), 3.72 (s, 3H, CH_3_O), 3.38 (s, 3H, CH_3_N); ^**13**^ **C‐NMR** (100 MHz, CDCl_3_) *δ*: 170.7, 157.67, 148.0, 138.8, 131.0, 128.0, 125.3, 114.4, 113.6, 55.4, 38.9; **MS** (DART+) m/z [M + H]^+^: 257 m/z; **HRMS** m/z calcd for ^12^C_15_^1^H_17_^14^N_2_^16^O_2_ [M + H]^+^, 257.12900; found 257.12924.

##### 4‐amino‐*N*‐(4‐chlorophenyl)‐*N*‐methylbenzamide (6d)

Purified by flash column chromatography (Hex‐AcOEt, 65:35). Yellowish solid in 57% yield. ^**1**^ **H‐NMR** (400 MHz, CDCl_3_) *δ*: 7.20–7.18 (AA´BB´, 2H), 7.13–7.11 (AA´BB´, 2H), 6.98–6.96 (AA´BB´, 2H), 6.43–6.41 (AA´BB´, 2H), 3.89 (br s, 2H, NH_2_), 3.43 (s, 3H, CH_3_N); ^**13**^ **C‐NMR** (100 MHz, CDCl_3_) *δ*: 170.7, 148.4, 144.5, 131.6, 131.2, 129.4, 128.1, 124.8, 113.8, 38.7; **MS** (DART+) m/z [M + H]^+^: 261 m/z; **HRMS** m/z calcd for ^12^C_14_^1^H_14_^35^Cl_1_^14^N_2_^16^O_1_ [M + H]^+^, 261.07947; found 261.07948.

##### 4‐amino‐*N*‐methyl‐*N*‐(naphthalen‐1‐yl)benzamide (6e)

Purified by flash column chromatography (Hex‐AcOEt, 1:1). Yellowish solid in 41% yield. ^**1**^ **H‐NMR** (400 MHz, CDCl_3_) *δ*: 7.99 (d, *J* = 8.4 Hz, 1H), 7.85 (d, *J* = 8.0 Hz, 1H), 7.69 (d, *J* = 8.4 Hz, 1H), 7.59–7.55 (m, 1H), 7.52–7.48 (m, 1H), 7.28–7.25 (m, 1H), 7.08 (br s, 3H), 6.21–6.19 (AA´BB´, 2H), 3.67 (br s, 2H, NH_2_), 3.46 (s, 3H, CH_3_N); ^**13**^ **C‐NMR** (100 MHz, CDCl_3_) *δ*: 171.8, 148.2, 142.2, 134.7, 130.2, 130.0, 128.8, 127.7, 127.3, 126.5, 126.3, 125.8, 125.3, 123.0, 113.6, 38.9; **MS** (DART+) m/z [M + H]^+^: 277 m/z; **HRMS** m/z calcd for ^12^C_18_^1^H_17_^14^N_2_^16^O_1_ [M + H]^+^, 277.13409; found 277.13412.

##### 4‐amino‐*N*‐methyl‐*N*‐(naphthalen‐2‐yl)benzamide (6f)

Purified by flash column chromatography (Hex‐AcOEt, 1:1). Yellowish solid in 58% yield. ^**1**^ **H‐NMR** (300 MHz, CDCl_3_) *δ*: 8.00 (d, *J* = 8.1 Hz, 1H), 7.86 (d, *J* = 7.8 Hz, 1H), 7.71 (d, *J* = 8.4 Hz, 1H), 7.59 (ddd, *J* = 8.1, 6.9 and 1.2 Hz, 1H), 7.52 (ddd, *J* = 8.6, 6.8 and 1.2 Hz, 1H), 7.09–7.06 (m, 3H), 6.23–6.21 (AA´BB´, 2H), 3.70 (br s, 2H, NH_2_), 3.47 (s, 3H, CH_3_N); ^**13**^ **C‐NMR** (75 MHz, CDCl_3_) *δ*: 171.8, 148.1, 142.2, 134.7, 130.2, 130.0, 128.8, 127.7, 127.3, 126.5, 126.4, 125.8, 125.3, 123.0, 113.6, 38.9; **MS** (DART+) m/z [M + H]^+^: 277 m/z; **HRMS** m/z calcd for ^12^C_18_^1^H_17_^14^N_2_^16^O_1_ [M + H]^+^, 277.13409; found 277.13429.

##### 4‐amino‐*N*‐(2,3‐dihydrobenzo[b][1,4]dioxin‐6‐yl)‐N‐methylbenzamide (6h)

Purified by flash column chromatography (Hex‐AcOEt, 4:6). Orange crystals in 50% yield. ^**1**^ **H‐NMR** (400 MHz, CDCl_3_) *δ*: 7.20–7.16 (AA´BB´, 2H), 6.69 (d, *J* = 8.4 Hz, 1H), 6.62 (d, *J* = 2.8 Hz, 1H), 6.50 (dd, *J* = 8.4 and 2.4 Hz, 1H), 6.45–6.42 (AA´BB´, 2H), 4.22 (br s, 4H), 3.80 (br s, 2H, NH_2_), 3.39 (s, 3H, CH_3_N); ^**13**^ **C‐NMR** (100 MHz, CDCl_3_) *δ*: 170.8, 153.0, 147.6, 146.2, 131.0, 127.9, 120.4, 117.8, 117.5, 115.8, 113.8, 64.3, 64.3, 53.5; **MS** (DART+) m/z [M + H]^+^: 285 m/z; **HRMS** m/z calcd for ^12^C_16_^1^H_16_^14^N_2_^16^O_3_ [M + H]^+^, 285.12392; found 285.12390.

##### Synthesis of Ureas (Series 1 and 2)

In a round‐bottom flask, the corresponding *N*‐methylbenzamide (0.11 mmol) was dissolved in MeCN (1.0 mmol), and propyl isocyanate (0.13 mmol) was added. The mixture was heated at 50 °C for 12 h. After the consumption of the starting material, acetonitrile was eliminated through distillation under reduced pressure, and the product was purified by flash column chromatography.

##### *N*‐methyl‐*N*‐phenyl‐4‐(3‐propylureido)benzamide (1a)

Purified by flash column chromatography (Hex‐AcOEt, 4:6). Beige solid in 63% yield. ^**1**^ **H‐NMR** (400 MHz, CDCl_3_) *δ*: 8.00 (s, 1H, NH), 7.22–7.17 (m, 2H), 7.15–7.12 (m, 1H), 7.11–7.08 (AA´BB´, 2H), 7.03–6.98 (m, 4H), 5.90 (t, *J* = 5.6 Hz, 1H, NH), 3.45 (s, 3H, CH_3_N), 3.10 (q, *J* = 6.8 Hz, 2H), 1.44 (sext, *J* = 7.2 Hz, 2H), 0.84 (t, *J* = 7.2 Hz, 3H, CH_3_); ^**13**^ **C‐NMR** (100 MHz, CDCl_3_) *δ*: 171.1, 156.0, 144.9, 142.0, 130.0, 129.4, 128.2, 126.8, 117.3, 41.7, 38.9, 23.5, 23.4, 11.5; **MS** (DART+) m/z [M + H]^+^: 312 m/z; **HRMS** m/z calcd for ^12^C_18_^1^H_22_^14^N_3_^16^O_2_ [M + H]^+^, 312.17120; found 312.17140.

##### *N*‐methyl‐4‐(3‐propylureido)‐*N*‐(p‐tolyl)benzamide (1b)

Purified by flash column chromatography (Hex‐AcOEt, 45:55). Yellowish solid in 69% yield. ^**1**^ **H‐NMR** (400 MHz, CDCl_3_) *δ*: 7.72 (s, 1H, NH), 7.11–7.09 (AA´BB´, 2H), 7.00–6.96 (m, 4H), 6.91–6.89 (AA´BB´, 2H), 5.74 (t, *J* = 5.6 Hz, 1H, NH), 3.43 (s, 3H, CH_3_N), 3.12 (q, *J* = 6.4 Hz, 2H), 2.26 (s, 3H, MePh), 1.46 (sext, *J* = 7.4 Hz, 2H), 0.88 (t, *J* = 7.4 Hz, 3H); ^**13**^ **C‐NMR** (100 MHz, CDCl_3_) *δ*: 171.2, 155.9, 142.3, 141.7, 136.8, 130.1, 130.0, 128.5, 126.6, 117.5, 41.8, 39.0, 23.4, 21.1, 11.5; **MS** (DART+) m/z [M + H]^+^: 326 m/z; **HRMS** m/z calcd for ^12^C_19_^1^H_24_^14^N_3_^16^O_2_ [M + H]^+^, 326.18685; found 326.18655.

##### *N*‐(4‐methoxyphenyl)‐*N*‐methyl‐4‐(3‐propylureido)benzamide (1c)

Purified by flash column chromatography (Hex‐AcOEt, 4:6). Light yellow solid in 33% yield. ^**1**^ **H‐NMR** (400 MHz, CDCl_3_) *δ*: 7.91 (s, 1H, NH), 7.10–7.07 (AA´BB´, 2H), 6.98–6.96 (AA´BB´, 2H), 6.94–6.92 (AA´BB´, 2H), 6.72–6.69 (AA´BB´, 2H), 5.86 (t, *J* = 5.6 Hz, 1H, NH), 3.71 (s, 3H, CH_3_O), 3.41 (s, 3H, CH_3_N), 3.09 (q, *J* = 7.0 Hz, 2H), 1.44 (sext, *J* = 7.2 Hz, 2H), 0.86 (t, *J* = 7.2 Hz, 3H); ^**13**^ **C‐NMR** (100 MHz, CDCl_3_) *δ*: 171.2, 158.2, 156.0, 141.7, 137.7, 129.9, 128.4, 128.0, 117.4, 114.6, 55.5, 41.8, 39.1, 23.4, 11.5; **MS** (DART+) m/z [M + H]^+^: 342 m/z; **HRMS** m/z calcd for ^12^C_19_^1^H_24_^14^N_3_^16^O_3_ [M + H]^+^, 342.18177; found 342.18187.

##### *N*‐(4‐chlorophenyl)‐*N*‐methyl‐4‐(3‐propylureido)benzamide (1d)

Purified by flash column chromatography (Hex‐AcOEt, 1:1). Yellowish solid in 59% yield. ^**1**^ **H‐NMR** (400 MHz, CDCl_3_ and some drops of DMSO‐d6) *δ*: 7.95 (s, 1H, NH), 7.17–7.14 (AA´BB´, 2H), 7.13–7.09 (m, 4H), 6.92–6.88 (AA´BB´, 2H), 5.66 (t, *J* = 5.6 Hz, 1H, NH), 3.36 (s, 3H, CH_3_N), 3.07 (q, *J* = 7.0 Hz, 2H), 1.42 (sext, *J* = 7.2 Hz, 2H), 0.83 (t, *J* = 7.4 Hz, 3H); ^**13**^ **C‐NMR** (100 MHz, CDCl_3_) *δ*: 170.5, 155.6, 143.9, 142.1, 131.7, 130.0, 129.3, 128.0, 127.8, 116.7, 41.4, 38.5, 23.3, 11.4; **MS** (DART+) m/z [M + H]^+^: 346 m/z; **HRMS** m/z calcd for ^12^C_18_^1^H_21_^35^Cl_1_^14^N_3_^16^O_2_ [M + H]^+^, 346.13223; found 346.13229.

##### *N*‐methyl‐*N*‐(naphthalen‐1‐yl)‐4‐(3‐propylureido)benzamide (1e)

Purified by flash column chromatography (Hex‐AcOEt, 3:7). Yellow solid in 24% yield.^**1**^ **H‐NMR** (400 MHz, CDCl_3_ and some drops of DMSO‐d6) *δ*: 7.89 (d, *J* = 8.4 Hz, 1H), 7.81 (br s, 1H), 7.76 (d, *J* = 8.0 Hz, 1H), 7.61 (d, *J* = 8.0 Hz, 1H), 7.48 (t, *J* = 7.6 Hz, 1H), 7.42 (t, *J* = 7.4 Hz, 1H), 7.17–7.15 (m, 1H), 7.03–6.98 (m, 3H), 6.62–6.90 (AA´BB´, 2H), 5.60 (br s, 1H, NH), 3.93 (s, 3H, CH_3_N), 2.97 (q, *J* = 5.6 Hz, 2H), 1.33 (sext, *J* = 6.8 Hz, 2H), 0.75 (t, *J* = 7.2 Hz, 3H); ^**13**^ **C‐NMR** (100 MHz, CDCl_3_) *δ*: 171.5, 155.5, 141.8, 141.4, 134.4, 129.7, 128.9, 128.6, 128.1, 127.8, 127.1, 126.3, 126.2, 125.5, 122.6, 116.4, 41.2, 38.6, 23.1, 11.2; **MS** (DART+) m/z [M + H]^+^: 362 m/z; **HRMS** m/z calcd for ^12^C_22_^1^H_24_^14^N_3_^16^O_2_ [M + H]^+^, 362.18685; found 362.18693.

##### *N*‐methyl‐*N*‐(naphthalen‐2‐yl)‐4‐(3‐propylureido)benzamide (1f)

Purified by flash column chromatography (Hex‐AcOEt, 4:6). Yellow solid in 38% yield. ^**1**^ **H‐NMR** (400 MHz, CDCl_3_ and some drops of DMSO‐d6) *δ*: 7.92 (d, *J* = 8.4 Hz, 1H), 7.80–7.79 (m, 2H), 7.64 (d, *J* = 8.0 Hz, 1H), 7.53 (t, *J* = 7.6 Hz, 1H), 7.47 (t, *J* = 7.7 Hz, 1H), 7.20 (t, *J* = 7.8 Hz, 1H), 7.06–7.00 (m, 3H), 6.94–6.92 (AA´BB´, 2H), 5.62 (t, *J* = 4.8 Hz, 1H, NH), 3.43 (s, 3H, CH_3_N), 2.99 (q, *J* = 5.6 Hz, 2H), 1.36 (sext, *J* = 7.4 Hz, 2H), 0.78 (t, *J* = 7.6 Hz, 3H); ^**13**^ **C‐NMR** (100 MHz, CDCl_3_) *δ*: 171.8, 155.6, 141.8, 141.5, 134.5, 129.8, 129.1, 128.7, 128.3, 127.9, 127.2, 126.4, 126.3, 125.6, 122.7, 116.6, 41.4, 38.7, 23.3, 11.3; **MS** (DART+) m/z [M + H]^+^: 362 m/z; **HRMS** m/z calcd for ^12^C_22_^1^H_24_^14^N_3_^16^O_2_ [M + H]^+^, 362.18685; found 362.18665.

##### *N*‐(2,3‐dihydrobenzo[b][1,4]dioxin‐6‐yl)‐*N*‐methyl‐4‐(3‐propylureido)benzamide (1g)

Purified by flash column chromatography (Hex‐AcOEt, 1:1). Orange solid in 85% yield. ^**1**^ **H‐NMR** (400 MHz, CDCl_3_) *δ*: 8.05 (br s, 1H, NH), 7.15–7.13 (AA´BB´, 2H), 7.04–7.02 (AA´BB´, 2H), 6.65 (d, *J* = 8.4 Hz, 1H), 6.60 (d, *J* = 2.4 Hz, 1H), 6.47 (dd, *J* = 8.8 and 2.4 Hz, 1H), 5.93 (t, *J* = 5.6 Hz, 1H, NH), 4.17 (br s, 1H), 3.38 (s, 3H, CH_3_N), 3.09 (q, *J* = 6.4 Hz, 2H), 1.42 (sext, *J* = 7.2 Hz, 2H), 0.84 (t, *J* = 7.4, 3H); ^**13**^ **C‐NMR** (100 MHz, CDCl_3_) *δ*: 171.2, 156.1, 143.8, 142.5, 141.9, 138.3, 129.8, 128.3, 120.2, 117.7, 117.3, 115.7, 64.3, 64.3, 41.7, 39.2, 23.4, 11.5; **MS** (EI) m/z [M]^+^: 369 m/z; **HRMS** m/z calcd for ^12^C_20_^1^H_23_^14^N_3_^16^O_4_ [M]^+^, 369.1689; found 369.1697.

##### *N*‐benzyl‐*N*‐methyl‐4‐(3‐propylureido)benzamide (2a, Mixture of Rotamers)

Purified by flash column chromatography (Hex‐AcOEt, 2:8). Light orange solid in 37% yield. ^**1**^ **H‐NMR** (700 MHz, CDCl_3_) *δ*: 8.23 (br s, 1H, NH), 7.37–7.36 (m, 2H), 7.33–7.28 (m, 4H), 7.24 (br s, 1H), 7.15 (br s, 1H), 6.02 (br s, 1H, NH), 4.74–4.56 (m, 2H, CH_2_N), 3.09 (q, *J* = 7.0 Hz, 2H), 3.01—2.9 (m, 3H, CH_3_N), 1.46 (sext, *J* = 7.0 Hz, 2H), 0.88 (t, *J* = 7.0, 3H); ^**13**^ **C‐NMR** (176 MHz, CDCl_3_) *δ*: 13C NMR (176 MHz, CDCl_3_) *δ*: 173.0, 172.3, 159.0, 156.1, 142.0, 136.6, 128.9, 128.2, 128.0, 127.9, 127.7, 126.8, 118.1, 55.5, 51.2, 41.6, 37.5, 33.5, 23.3, 11.4; **MS** (DART+) m/z [M + H]^+^: 326 m/z; **HRMS** m/z calcd for ^12^C_19_^1^H_24_^14^N_3_^16^O_2_ [M + H]^+^, 326.18685; found 326.18649.

##### *N*‐methyl‐*N*‐(4‐methylbenzyl)‐4‐(3‐propylureido)benzamide (2b, Mixture of Rotamers)

Purified by flash column chromatography (Hex‐AcOEt, 2:8). Colorless crystals in 82% yield. ^**1**^ **H‐NMR** (700 MHz, CDCl_3_) *δ*: 7.98–7.92 (m, 1H, NH), 7.25–7.24 (AA´BB´, 2H), 7.20 (br s, 1H), 7.16–7.15 (AA´BB´, 2H), 7.13–7.12 (AA´BB´, 2H), 7.01 (br s, 1H), 5.88–5.81 (m, 1H, NH), 4.86–4.49 (m, 2H), 3.13 (br s, 2H), 2.98–2.89 (m, 3H), 2.34 (s, 3H), 1.49–1.42 (m, 2H), 0.89–0.87 (m, 3H); ^**13**^ **C‐NMR** (176 MHz, CDCl_3_) *δ*: 173.0, 172.4, 156.1, 141.9, 137.5, 133.7, 133.4, 129.7, 128.6, 126.9, 118.5, 55.4, 51.1, 41.8, 37.5, 33.5, 23.5, 21.2, 11.5; **MS** (DART+) m/z [M + H]^+^: 340 m/z; **HRMS** m/z calcd for ^12^C_20_^1^H_26_^14^N_3_^16^O_2_ [M + H]^+^, 340.20250; found 340.20262.

##### *N*‐(4‐methoxybenzyl)‐*N*‐methyl‐4‐(3‐propylureido)benzamide. (2c, Mixture of Rotamers)

Purified by flash column chromatography (Hex‐AcOEt, 2:8). Orange solid in 89% yield. ^**1**^ **H‐NMR** (700 MHz, DMSO‐d6) *δ*: 8.60 (s, 1H, NH), 7.43–7.42 (AA´BB´, 2H), 7.32–7.31 (AA´BB´, 2H), 7.12 (br s, 2H), 6.93–6.91 (AA´BB´, 2H), 6.20 (t, *J* = 5.6 Hz, 1H, NH),4.52 (br s, 2H, CH_2_N), 3.74 (s, 3H, CH_3_O), 3.04 (q, *J* = 6.3 Hz, 2H), 2.82 (s, 3H, CH_3_N), 1.43 (sext, *J* = 7.0 Hz, 2H), 0.86 (t, *J* = 7.0 Hz, 3H); ^**13**^ **C‐NMR** (176 MHz, CDCl_3_) *δ*: 171.3, 159.0, 158.6, 155.5, 142.3, 129.7, 128.8, 128.4, 117.2, 114.5, 55.5, 54.2, 49.8, 41.3, 37.2, 33.0, 23.4, 11.8; **MS** (DART+) m/z [M + H]^+^: 356 m/z; **HRMS** m/z calcd for ^12^C_20_^1^H_26_^14^N_3_^16^O_3_ [M + H]^+^, 356.19742; found 356.19725.

##### *N*‐(4‐chlorobenzyl)‐*N*‐methyl‐4‐(3‐propylureido)benzamide (2d, Mixture of Rotamers)

Purified by flash column chromatography (Hex‐AcOEt, 2:8). Brown solid in 17% yield. ^**1**^ **H‐NMR** (700 MHz, CDCl_3_) *δ*: 8.23 (s, 1H, NH), 7.35–7.34 (AA´BB´, 2H), 7.31–7.29 (m, 4H), 7.11 (br s, 2H), 6.00 (br s, 1H, NH), 4.69–4.50 (m, 2H, CH_2_N), 3.11 (q, *J* = 5.6 Hz, 2H), 2.95 (br s, 3H),1.49 (sext, *J* = 7.0 Hz, 2H), 0.91 (t, *J* = 7.7 Hz, 3H); ^**13**^ **C**‐**NMR** (176 MHz, CDCl_3_) *δ*: 172.9, 172.2, 159.0, 156.2, 142.3, 135.5, 133.6, 129.5, 129.1, 128.2, 118.2, 54.9, 50.7, 42.4, 37.6, 33.6, 23.6, 11.5, 11.5; **MS** (DART+) m/z [M + H]^+^: 360 m/z; **HRMS** m/z calcd for ^12^C_19_^1^H_23_^35^Cl_1_^14^N_3_^16^O_2_ [M + H]^+^, 360.14788; found 360.14801.

##### *N*‐benzyl‐*N*‐ethyl‐4‐(3‐propylureido)benzamide (2e, Mixture of Rotamers)

Purified by flash column chromatography (Hex‐AcOEt, 2:8). White solid in 27% yield. ^**1**^ **H‐NMR** (400 MHz, DMSO‐d6) *δ*: 8.38 (s, 1H, NH), 7.44–7.42 (AA´BB´, 2H), 7.37–7.33 (AA´BB´, 2H), 7.30–7.27 (m, 5H), 6.09 (t, *J* = 5.6 Hz, 1H, NH), 4.60 (s, 2H, CH_2_N), 3.29 (q, *J* = 6.8 Hz, 2H), 3.07 (q, *J* = 6.4 Hz, 2H), 1.46 (sext, *J* = 6.8 Hz, 2H), 1.05 (t, *J* = 7.0 Hz, 3H), 0.89 (t, *J* = 7.4 Hz, 3H); ^**13**^ **C**‐**NMR** (176 MHz, CDCl_3_) *δ*: 170.8, 155.1, 141.7, 137.9, 128.9, 128.6, 127.4, 116.9, 51.5, 46.8, 43.2, 40.89, 23.0, 13.7, 11.3; **MS** (DART+) m/z [M + H]^+^: 340 m/z; **HRMS** m/z calcd for ^12^C_20_^1^H_26_^14^N_3_^16^O_2_ [M + H]^+^, 340.20250; found 340.20256.

##### *N*‐methyl‐*N*‐(naphthalen‐2‐ylmethyl)‐4‐(3‐propylureido)benzamide (2f, Mixture of Rotamers)

Purified by flash column chromatography (Hex‐AcOEt, 2:8). Orange solid in 31% yield. ^**1**^ **H‐NMR** (700 MHz, DMSO‐d6) *δ*: 8.62(s, 1H, NH), 7.93–7.91 (m, 3H), 7.79 (br s, 1H), 7.53 (ddd, *J* = 7.0, 5.6 and 1.4 Hz, 1H), 7.53 (ddd, *J* = 7.0, 6.3 and 1.4 Hz, 1H), 7.45 (br s, 3H), 7.39 (br s, 2H), 6.21 (t, *J* = 5.6 Hz, 1H), 4.78 (br s, 2H, CH_2_N), 3.04 (q, *J* = 7.0 Hz, 2H), 2.92 (s, 3H, CH_3_N), 1.44 (sext, *J* = 7.0 Hz, 2H), 0.87 (t, *J* = 7.0 Hz, 3H); ^**13**^ **C**‐**NMR** (176 MHz, CDCl_3_) *δ*: 170.6, 155.0, 142.0, 135.1, 133.0, 132.3, 129.0, 128.4, 128.3, 128.0 (br s), 127.7, 127.6, 126.3, 125.9, 116.8, 54.5, 50.2, 37.1, 33.1, 22.9, 11.3; **MS** (DART+) m/z [M + H]^+^: 376 m/z; **HRMS** m/z calcd for ^12^C_23_^1^H_26_^14^N_3_^16^O_2_ [M + H]^+^, 376.20250; found 376.20244.

##### *N*‐methyl‐*N*‐phenethyl‐4‐(3‐propylureido)benzamide (2g, Mixture of Rotamers)

Purified by flash column chromatography (Hex‐AcOEt, 2:8). Colorless crystals in 69% yield. ^**1**^ **H‐NMR** (700 MHz, DMSO‐d6) *δ*: 8.57 (s, 1H, NH), 7.38 (br s, 2H), 7.27 (br s, 3H), 7.21–7.20 (m, 2H), 7.03 (br s, 2H), 6.19 (t, *J* = 5.6 Hz, 1H, NH), 3.62–3.43 (m, 2H, CH_2_N), 3.04 (q, *J* = 6.3 Hz, 2H), 2.96 (br s, 2H), 2.84 (br s, 2H), 1.44 (sext, *J* = 7.0 Hz, 2H), 0.87 (t, *J* = 7.0 Hz, 3H); ^**13**^ **C**‐**NMR** (176 MHz, CDCl_3_) *δ*: 170.9, 169.9, 155.0, 141.6, 139.2, 138.5, 128.8, 128.7, 128.4, 127.9, 127.4, 126.2, 116.6, 52.3, 48.6, 40.9, 37.6, 33.9, 32.8, 22.9, 11.3; **MS** (DART+) m/z [M + H]^+^: 340 m/z; **HRMS** m/z calcd for ^12^C_20_^1^H_26_^14^N_3_^16^O_2_ [M + H]^+^, 340.20250; found 340.20225.

##### Synthesis of Piperidine‐Containing Ureas 3a–d

In a round‐bottom flask, 4‐aminobenzoic acid (**7**, 2.17 mmol) and propyl isocyanate (3.25 mmol) were dissolved in MeCN (200 mM). The reaction was stirred at 40 °C for 24 h. The white solid was filtered and used in the next step.

The ureido compound **8** (0.225 mmol), 4‐DMAP (0.061 mmol) were dissolved in THF (137 mM) at 0 °C. Afterward, triethylamine (0.245 mmol) and piperidone monohydrate hydrochloride (0.205 mmol) were sequentially added. After 10 min of stirring, dicyclohexylcarbodiimide (DCC, 0.266 mmol) was added, and the mixture was allowed to warm to room temperature. The mixture was reacted for 24 h at room temperature. After the consumption of the starting material, the solid was filtered, and the organic phase was evaporated. The residue was redissolved with MeCN (2 mL) and cooled for 1–2 h. The excess of DCU was removed by filtration, and the desired piperidone was used without further purification.

Finally, piperidone **9** (0.099 mmol), the corresponding aniline (0.151 mmol), a few drops of glacial acetic acid, and Na_2_SO_4_ (50 mg) were suspended in EtOH (60 mM). The imine was formed after 24 h of stirring at 50 °C. Then, the reaction was cooled to 0 °C and NaBH_3_CN (0.297 mmol) was added in two portions. After completion of the reaction, the ethanol was removed, and a saturated solution of sodium bicarbonate was added (5 mL). The product was extracted with ethyl acetate (2 × 10 mL). The product was purified by flash column chromatography with an adequate mixture of solvents.

##### 1‐(4‐(4‐(phenylamino)piperidine‐1‐carbonyl)phenyl)‐3‐propylurea (3a)

Obtained as a light brown solid in 27% yield after purification by flash column chromatography (Hex‐AcOEt 2:8). ^**1**^ **H‐NMR** (400 MHz, CDCl_3_ and DMSO‐_d6_) *δ*: 8.02 (s, 1H, NH), 7.34–7.32 (AA´BB´, 2H), 7.24–7.22 (AA´BB´, 2H), 7.13–7.09 (comp, 2H), 6.66–6.61 (comp, 1H), 6.57–6.54 (m, 2H), 5.72 (t, *J* = 6.0 Hz, 1H, NH), 4.17–3.92 (m, 1H), 3.52–3.45 (m, 1H), 3.12 (q, *J* = 6.4 Hz, 2H), 3.06 (br s, 1H), 2.24 (br s, 1H), 2.08–1.96 (m, 2H), 1.47 (sext, *J* = 7.2 Hz, 2H), 1.36 (br s, 2H); 0.88 (t, *J* = 7.2 Hz, 3H); ^**13**^ **C‐NMR** (100 MHz, CDCl_3_ and DMSO‐_d6_) *δ*: 170.7, 155.9, 146.7, 141.9, 129.4, 128.5, 128.1, 117.8, 117.5, 113.3, 50.0, 41.5, 32.7 (br s), 23.4, 11.4; **MS** (DART+) m/z [M + H]^+^: 381 m/z; **HRMS** m/z calcd for ^12^C_22_^1^H_29_^14^N_4_^16^O_2_ [M + H]^+^, 381.22905; found 381.22921.

##### 1‐propyl‐3‐(4‐(4‐(*p*‐tolylamino)piperidine‐1‐carbonyl)phenyl)urea (3b)

Obtained as a reddish solid in 46% yield after purification by flash column chromatography (Hex‐AcOEt 2:8). ^**1**^ **H‐NMR** (400 MHz, CDCl3 and DMSO‐d6) *δ*: 8.02 (s, 1H, NH), 7.17–7.15 (AA´BB´, 2H), 7.10–7.08 (AA´BB´, 2H), 7.00–6.98 (AA´BB´, 2H), 6.55–6.53 (AA´BB´, 2H), 5.94 (t, *J* = 6.0 Hz, 1H), 4.48 (br s, 1H), 3.80 (br s, 1H), 3.53–3.46 (m, 1H), 3.17 (q, *J* = 6.8 Hz, 2H), 3.10 (br s, 2H), 2.23 (s, 3H), 2.06 (br s, 2H), 1.51 (sext, *J* = 7.2 Hz, 2H), 1.43–1.29 (m, 2H), 0.92 (t, *J* = 7.2 Hz, 3H); ^**13**^ **C‐NMR** (100 MHz, CDCl_3_ and DMSO‐_d6_) *δ*: 171.1, 156.2, 144.4, 141.9, 130.0, 128.4, 128.0, 127.2, 118.5, 113.8, 50.4, 41.8, 32.4 (br s), 29.8, 23.5, 20.5, 11.5; **MS** (DART+) m/z [M + H]^+^: 395 m/z; **HRMS** m/z calcd for ^12^C_23_^1^H_31_^14^N_4_^16^O_2_ [M + H]^+^, 395.24470; found 395.24465.

##### 1‐(4‐(4‐((4‐methoxyphenyl)amino)piperidine‐1‐carbonyl)phenyl)‐3‐propylurea (3c)

Obtained as a dark solid in 72% yield after purification by flash column chromatography (Hex‐AcOEt 1:9). ^**1**^ **H‐NMR** (400 MHz, CDCl3 and DMSO‐d6) *δ*: 8.11 (s, 1H, NH), 7.27–7.24 (AA´BB´, 2H), 7.19–7.17 (AA´BB´, 2H), 6.74–6.70 (AA´BB´, 2H), 6.57–6.53 (AA´BB´, 2H), 5.85 (t, *J* = 5.6 Hz, 1H, NH), 4.41 (br s, 1H), 3.69 (s, 4H), 3.42–3.35 (m, 1H), 3.10 (q, *J* = 6.8 Hz, 2H), 3.03 (br s, 2H), 2.03–1.93 (comp, 2H), 1.44 (sext, *J* = 7.2 Hz, 2H), 1.34 (br s, 2H), 0.086 (t, *J* = 7.2, 3H); ^**13**^ **C‐NMR** (100 MHz, CDCl_3_ and DMSO‐_d6_) *δ*: 170.8, 156.1, 152.4, 141.9, 140.7, 128.4, 128.1, 118.0, 115.3, 115.1, 55.8, 51.2, 41.6, 32.6, 29.7, 23.4, 11.5; **MS** (DART+) m/z [M + H]^+^: 411 m/z; **HRMS** m/z calcd for ^12^C_23_^1^H_31_^14^N_4_^16^O_3_ [M + H]^+^, 411.23961; found 411.23939.

##### 1‐(4‐(4‐((4‐chlorophenyl)amino)piperidine‐1‐carbonyl)phenyl)‐3‐propylurea (3d)

Obtained as a light brown solid in 46% yield after purification by flash column chromatography (Hex‐AcOEt 2:8). ^**1**^ **H‐NMR** (400 MHz, CDCl_3_) *δ*: 7.95 (s, 1H, NH), 7.16–7.13 (AA´BB´, 2H), 7.12–7.09 (comp, 4H), 6.55–6.51 (AA´BB´, 2H), 5.88 (t, *J* = 5.6 Hz, 1H; NH), 4.40 (br s, 1H), 3.85–3.69 (m, 1H), 3.50–3.45 (m, 1H), 3.17 (q, *J* = 6.8 Hz, 2H), 3.12–3.04 (m, 2H), 2.17–1.87 (m, 2H), 1.51 (sext, 2 Hz, 2H), 1.34 (br s, 2H), 0.92 (t, *J* = 7.2 Hz, 3H); ^**13**^ **C‐NMR** (100 MHz, CDCl_3_) *δ*: 171.0, 156.2, 145.3, 141.8, 129.4, 128.4, 122.0, 122.3, 118.6, 114.5, 50.1, 41.9, 34.0, 29.8, 23.5, 11.5; **MS** (DART+) m/z [M + H]^+^: 415 m/z; **HRMS** m/z calcd for ^12^C_22_^1^H_28_^35^Cl_1_^14^N_4_^16^O_2_ [M + H]^+^, 415.19008; found 415.19055.

##### Antibacterial Screening and IC_50_ Determination against MRSA

Antibacterial activity was evaluated against methicillin‐resistant *Staphylococcus aureus* (ATCC 33591) and a clinical isolate. The experimental workflow was slightly modified from the standardized protocol described by Wiegand and cols. as follows.^[^ ^33^ ^]^ For the initial screening, each bacterial strain was cultivated overnight at 37 °C in 5 mL of Mueller–Hinton Broth (MHB) medium. The following day, fresh subcultures were prepared under the same conditions and inoculated to an optical density of 0.01 at 600 nm (OD_600_). Aliquots of 90 μL of this bacterial suspension were transferred into 96‐well flat‐bottom microtiter plates. Subsequently, 10 μL of each test compound (dissolved in DMSO) was added to achieve a final concentration of 50 μM per well; then, the plates were incubated at 37 °C for 24 h. Each assay included a sterile control (medium and DMSO only), and a growth control (medium, bacteria, and DMSO without compounds). After incubation, samples were analyzed by recording OD_600_ with a microplate spectrophotometer. Bacterial growth was calculated by using the following equation:

(1) $$\text{\% Growth}=\frac{\left({\text{Sample}}_{A600}-{\text{Sterile Control}}_{A600}\right)}{\left({\text{Growth Control}}_{A600}-{\text{Sterile Control}}_{A600}\right)}\times 100$$

Five compounds were selected for the determination of their half‐maximal inhibitory concentration (IC_50_). For these assays, experiments were conducted as indicated, except that compounds were tested in a 1–500 μM range. All experiments were performed in triplicate; data were analyzed using GraphPad Prism software version 8.

##### Evaluation of Lead Compounds on S. aureus Strains Biofilm Formation

The antibiofilm potential of selected lead compounds was evaluated using a crystal violet staining assay, as described by Haney with slight modifications.^[^ ^34^ ^]^ Briefly, 5 mL of Tryptic Soy Broth (TSB) cultures were inoculated with three isolated colonies of each strain and incubated at 37 °C for 18 h under agitation. A subculture was then prepared and diluted to an OD_600_ of 0.05 in fresh TSB medium supplemented with 1% glucose. Aliquots of 180 µL were dispensed into flat‐bottom 96‐well plates, followed by the addition of 20 µL of compound solution (dissolved in DMSO) to achieve a final concentration of 50 µM. Plates were incubated statically at 37 °C for 24 h. To ensure accurate results, a growth control (without compound) and a sterile control (without bacteria) were included in each experiment. After incubation, planktonic cells were discarded, and wells were gently washed three times with sterile distilled water. Plates were dried at room temperature for 30 min, and biofilms were stained with 205 µL of 0.1% crystal violet for 30 min. Excess dye was removed, and wells were washed three times with sterile distilled water. The retained dye, corresponding to biofilm biomass, was solubilized with 210 µL of 33% (v/v) acetic acid, and absorbance was measured at 580 nm using a microplate reader.

The percentage of biofilm formation was calculated using the following Equation:

(2) $$\%\text{Biofilm formation}=\frac{\left({\text{Sample}}_{A580}-{\text{Sterile Control}}_{A580}\right)}{\left({\text{Growth Control}}_{A580}-{\text{Sterile Control}}_{A580}\right)}\times 100$$

##### Computational Study

Potential energy surface scans were performed by systematically varying the dihedral angles of interest while optimizing all other degrees of freedom. All calculations were carried out using the PBEh‐3c composite method^[^ ^35^ ^]^ as implemented in the ORCA 6 software package.^[^ ^36^ ^]^ This method provides a cost‐efficient approach with good accuracy for noncovalent interactions and conformational energies, and therefore, is suitable for the exploration of torsional profiles in the studied systems.

## Conflict of Interest

The authors declare no conflict of interest.

## Supporting information

Supplementary Material

## Data Availability

The data that support the findings of this study are available from the corresponding author upon reasonable request.

## References

[1] R. Gaynes , Emerging Infect. Dis. 2017, 23, 849.

[2] F. Bosch , L. Rosich , Pharmacology 2008, 82, 171. 18679046 10.1159/000149583 PMC2790789

[3] T. M. Uddin , A. J. Chakraborty , A. Khusro , B. R. M. Zidan , S. Mitra , T. B. Emran , K. Dhama , M. K. H. Ripon , M. Gajdács , M. U. K. Sahibzada , M. J. Hossain , N. Koirala , J. Infect. Public Health 2021, 14, 1750. 34756812 10.1016/j.jiph.2021.10.020

[4] D. Kumar , N. Sarkar , K. K. Roy , D. Bisht , D. Kumar , B. Mandal , M. Rajagopal , Y. N. Dey , Curr. Drug Targets 2023, 24, 627. 37291783 10.2174/1389450124666230608150759

[5] M. Rosas‐Cruz , A. Madariaga‐Mazón , C. D. García‐Mejía , E. Hernández‐Vázquez , H. Gómez‐Velsaco , R. S. Farías‐Gaytán , J. A. Hermoso , S. Martínez‐Caballero , ACS Omega 2024, 9, 46461. 39583660 10.1021/acsomega.4c07964 PMC11579945

[6] T. M. Belete , Hum. Microbiome J. 2019, 11, 100052.

[7] M. Wang , Y. Lian , Y. Wang , L. Zhu , Environ. Pollut. 2023, 322, 121238. 36758922 10.1016/j.envpol.2023.121238

[8] F. Wittke , C. Vincent , J. Chen , B. Heller , H. Kabler , J. S. Overcash , F. Leylavergne , G. Dieppois , Antimicrob. Agents Chemother. 2020, 64, e00250. 32747361 10.1128/AAC.00250-20 PMC7508579

[9] E. J. A. Douglas , S. W. Wulandari , S. D. Lovell , M. Laabei , Microb. Biotechnol. 2023, 16, 1456. 37178319 10.1111/1751-7915.14268 PMC10281381

[10] J. E. Cronan , Curr. Opin. Chem. Biol. 2018, 47, 60. 30236800 10.1016/j.cbpa.2018.08.004 PMC6289770

[11] A. Karioti , H. Skaltsa , X. Zhang , P. J. Tonge , R. Perozzo , M. Kaiser , S. G. Franzblau , D. Tasdemir , Phytomedicine 2008, 15, 1125. 18424102 10.1016/j.phymed.2008.02.018

[12] P. Rana , S. M. Ghouse , R. Akunuri , Y. V. Madhavi , S. Chopra , S. Nanduri , Eur. J. Med. Chem. 2020, 208, 112757. 32883635 10.1016/j.ejmech.2020.112757

[13] W. Balemans , N. Lounis , R. Gilissen , J. Guillemont , K. Simmen , K. Andries , A. Koul , Nature 2010, 463, E3. 20090698 10.1038/nature08667

[14] E. Hernández‐Vázquez , Á. Ramírez‐Trinidad , C. E. Tovar‐Román , J. A. R. Chávez , E. Huerta‐Salazar , Bioorg. Med. Chem. Lett. 2024, 112, 129936. 39214507 10.1016/j.bmcl.2024.129936

[15] L. Bibens , J.‐P. Becker , A. Dassonville‐Klimpt , P. Sonnet , Pharmaceuticals 2023, 16, 425. 36986522 10.3390/ph16030425 PMC10054515

[16] W. H. Miller , M. A. Seefeld , K. A. Newlander , I. N. Uzinskas , W. J. Burgess , D. A. Heerding , C. C. K. Yuan , M. S. Head , D. J. Payne , S. F. Rittenhouse , T. D. Moore , S. C. Pearson , V. Berry , W. E. DeWolf , P. M. Keller , B. J. Polizzi , X. Qiu , C. A. Janson , W. F. Huffman , J. Med. Chem. 2002, 45, 3246. 12109908 10.1021/jm020050+

[17] G. Akhter , H. Hamid , B. Dhawan , A. K. Das , M. A. Tantray , M. S. Alam , K. Sharma , ChemistrySelect 2024, 9, e202404473.

[18] N. Kaplan , M. Albert , D. Awrey , E. Bardouniotis , J. Berman , T. Clarke , M. Dorsey , B. Hafkin , J. Ramnauth , V. Romanov , M. B. Schmid , R. Thalakada , J. Yethon , H. W. Pauls , Antimicrob. Agents Chemother. 2012, 56, 5865. 22948878 10.1128/AAC.01411-12 PMC3486558

[19] L. Zhang , J. Yang , X. Xu , J. Zhang , Z. Qiu , Y. Ju , B. Luo , Y. Liu , X. Gou , J. Sui , B. Chen , Y. Wang , T. Tao , L. He , T. Yang , Y. Luo , J. Med. Chem. 2024, 67, 10096. 38845361 10.1021/acs.jmedchem.4c00320

[20] E. N. Parker , B. N. Cain , B. Hajian , R. J. Ulrich , E. J. Geddes , S. Barkho , H. Y. Lee , J. D. Williams , M. Raynor , D. Caridha , A. Zaino , M. Shekhar , K. A. Muñoz , K. M. Rzasa , E. R. Temple , D. Hunt , X. Jin , C. Vuong , K. Pannone , A. M. Kelly , M. P. Mulligan , K. K. Lee , G. W. Lau , D. T. Hung , P. J. Hergenrother , ACS Cent. Sci. 2022, 8, 1145. 36032774 10.1021/acscentsci.2c00598 PMC9413440

[21] S. R. Shaikh , R. L. Gawade , D. Kumar , A. Kotmale , R. G. Gonnade , T. Stürzer , Cryst. Growth Des. 2019, 19, 5665.

[22] T. Kronenberger , P. de Oliveira Fernades , I. D. Franco , A. Poso , V. Gonçalves Maltarollo , ChemMedChem 2019, 14, 1995. 31670463 10.1002/cmdc.201900415 PMC6916556

[23] K. A. Bobesh , J. Renuka , R. R. Srilakshmi , S. Yellanki , P. Kulkarni , P. Yogeeswari , D. Sriram , Bioorg. Med. Chem. 2016, 24, 42. 26678175 10.1016/j.bmc.2015.11.039

[24] S. Tahir , T. Mahmood , F. Dastgir , I. Haq , A. Waseem , U. Rashid , Eur. J. Med. Chem. 2019, 166, 224. 30711832 10.1016/j.ejmech.2019.01.062

[25] F. Elghali , I. Ibrahim , M. Guesmi , F. Frikha , S. Mnif , Braz. J. Microbiol. 2024, 55, 2057. 38775905 10.1007/s42770-024-01374-2 PMC11405564

[26] Synth. Commun. 2021, 51, 786.

[27] D. Gül , S. Arkan‐Ozdemir , O. Yücel , E. Yıldırım , G. Kalyon , E. Ilhan‐Sungur , S. Emik , A. Erol , N. T. Kara , Microsc. Res. Tech. 2025, 88, 1019. 39648286 10.1002/jemt.24768

[28] A. C. Muñoz‐Estrada , C. E. Tovar‐Roman , C. D. García‐Mejía , R. García‐Contreras , E. Hernández Vázquez , ChemMedChem 2025, 20 *(9* *)*, e202400879. 39833117 10.1002/cmdc.202400879 PMC12058234

[29] R. A. Harper , G. H. Carpenter , G. B. Proctor , R. D. Harvey , R. J. Gambogi , A. R. Geonnotti , R. Hider , S. A. Jones , Colloids Surf., B 2019, 173, 392. 10.1016/j.colsurfb.2018.09.018 30317126

[30] A. Mannschreck , R. Kiesswetter , E. von Angerer , J. Chem. Educ. 2007, 84, 2012.

[31] A. Daina , O. Michielin , V. Zoete , Sci. Rep. 2017, 7, 42717. 28256516 10.1038/srep42717 PMC5335600

[32] S. M. Soto , Virulence 2013, 4, 223. 23380871 10.4161/viru.23724 PMC3711980

[33] I. Wiegand , K. Hilpert , R. E. W. Hancock , Nat. Protoc. 2008, 3, 163. 18274517 10.1038/nprot.2007.521

[34] E. F. Haney , M. J. Trimble , R. E. W. Hancock , Nat. Protoc. 2021, 16, 2615. 33911258 10.1038/s41596-021-00515-3

[35] S. Grimme , J. G. Brandenburg , C. Bannwarth , A. Hansen , J. Chem. Phys. 2015, 143, 054107. 26254642 10.1063/1.4927476

[36] F. Neese , WIREs Comput. Mol. Sci. 2025, 15, e70019.

