# Supplementary material for: Synthesis of Urea‐Containing Derivatives and their Application as Potential Anti‐Methicillin‐Resistant Staphylococcus Aureus Agents
Source: ChemMedChem. 2025 Sep 5;20(19):e202500521. doi: 10.1002/cmdc.202500521 (PMC12503907; doi:10.1002/cmdc.202500521)
Supplement: Supplementary file 1 — Supplementary Material [file CMDC-20-e202500521-s001.pdf]

–*SUPPORTING INFORMATION*–

Synthesis of urea-containing derivatives and their application as anti-MRSA agents

Jorge A. González-Cruz[a], Gerardo González-Gallardo[a], J. Ricardo Pérez-Velázquez[b], Carlos D. García-Mejía[a], José Manuel Guevara-Vela[c], Jesús A. Oria-Hernández[b], Tomás Rinza-Rocha[d], **Eduardo Hernández-Vázquez*[a]**

a. Departamento de Química Orgánica, Instituto de Química, Universidad Nacional Autónoma de México (UNAM), CDMX, México.

b. Laboratorio de Bioquímica-Genética, Instituto Nacional de Pediatría, Secretaría de Salud Ciudad de México, México.

c. School of Engineering and Physical Sciences, Heriot-Watt University, Edinburgh EH14 4AS Scotland, U.K.

d. Departamento de Fisicoquímica, Instituto de Química, Universidad Nacional Autónoma de México (UNAM), CDMX, México.

CONTENT:

Characterization of aminoamides **6a–h**.......... S2

Characterization of ureas (Series **1–3**).......... S16

X-Ray data.......... S53

SwissADME predictions.......... S56

CHARACTERIZATION OF AMINOAMIDES 6a–h

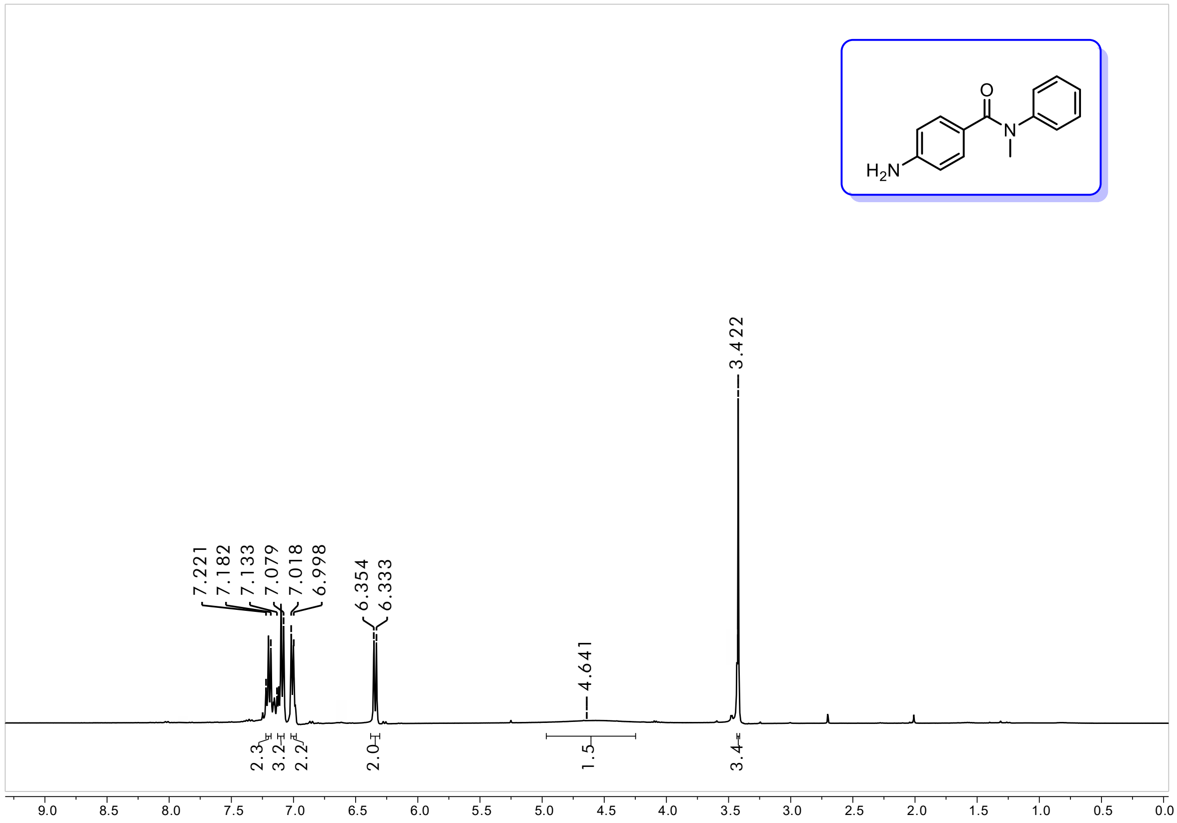

Spectrum 1. ^{1}H-NMR of 4-amino-*N*-methyl-N-phenylbenzamide (6a).

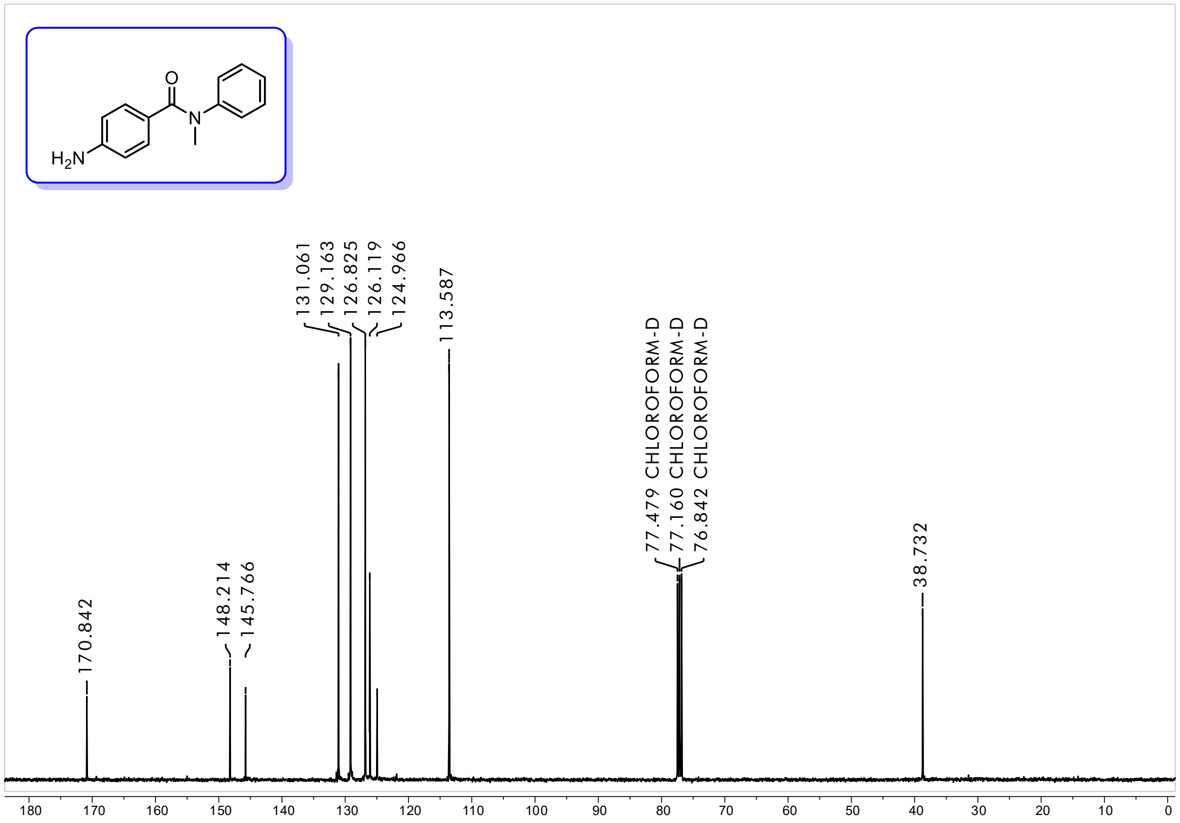

Spectrum 2. ^{13}C-NMR of 4-amino-*N*-methyl-N-phenylbenzamide (6a).

Description:
Ionization Mode:ESI+
History:Determine m/z[Peak Detect[Centroid,30,Area];Correct Base[5.0%]];Correct Base[5.0%];Average(MS[1] 1..1)

Mass Calibration data:CAL_PEG_600
Created:1/15/2025 11:45:18 AM
Created by:AccuTOF

Charge number:1
Element:^{12}C:0 .. 14, ^{1}H:0 .. 16, ^{14}N:2 .. 2, ^{16}O:1 .. 1

Tolerance:5.00(ppm), 5.00 .. 15.00(mmu)

Unsaturation Number:-1.0 .. 100.0 (Fraction:Both)

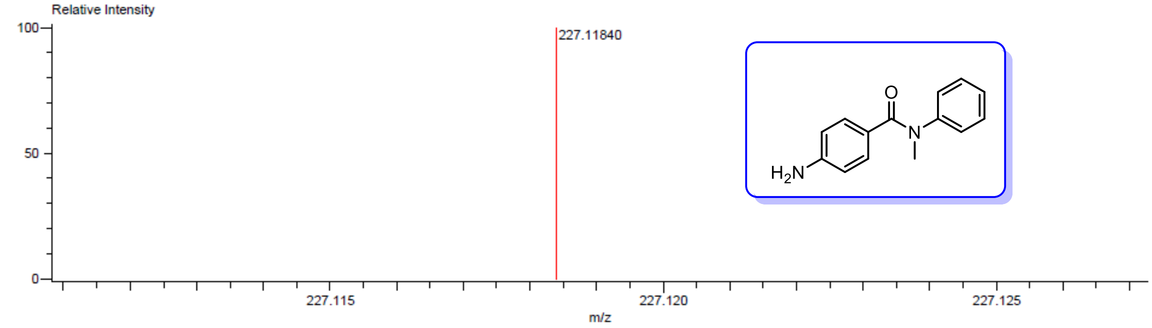

Mass	Intensity	Calc. Mass	Mass Difference (mmu)	Mass Difference (ppm)	Possible Formula	Unsaturation Number
227.11840	71540.25	227.11844	-0.04	-0.18	$^{12}C_{14}{}^{1}H_{15}{}^{14}N_{2}{}^{16}O_{1}$	8.5

Spectrum 3. HRMS of 4-amino-*N*-methyl-N-phenylbenzamide (6a).

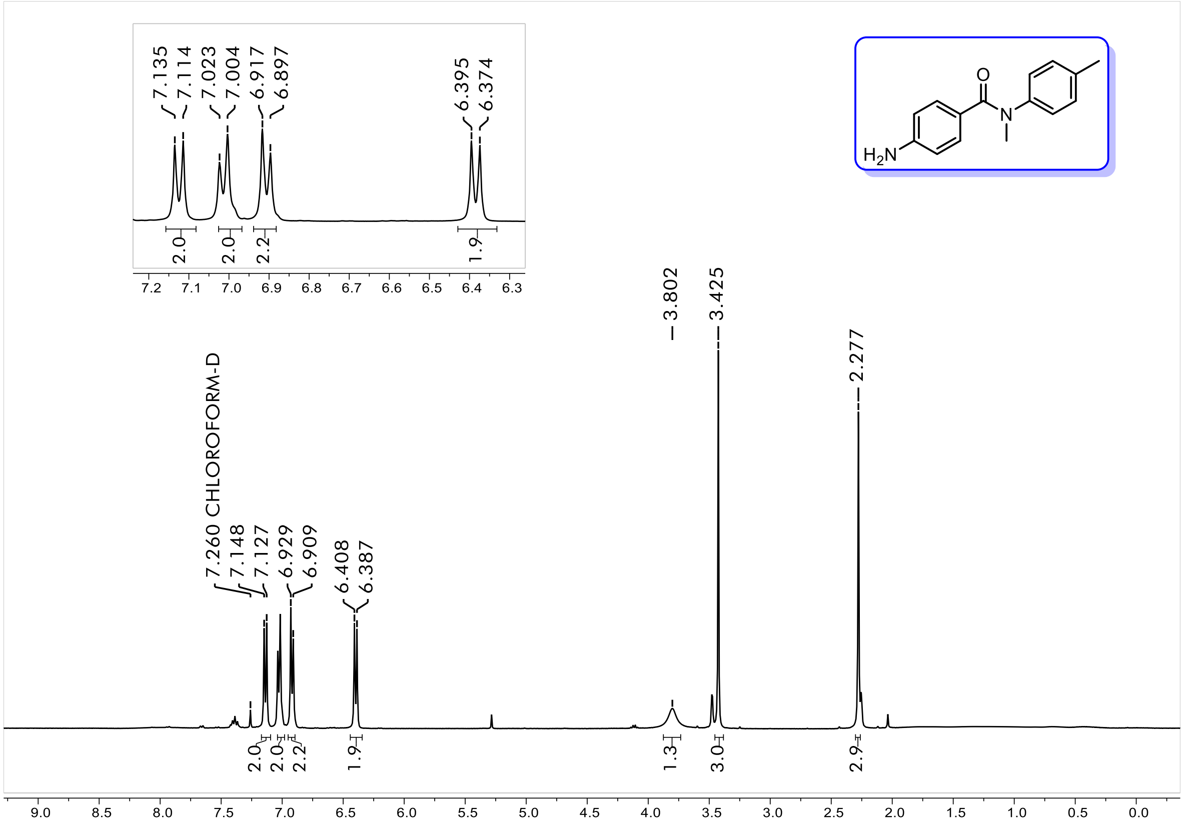

Spectrum 4. ^{1}H-NMR of 4-amino-*N*-methyl-*N*-(p-tolyl)benzamide (6b).

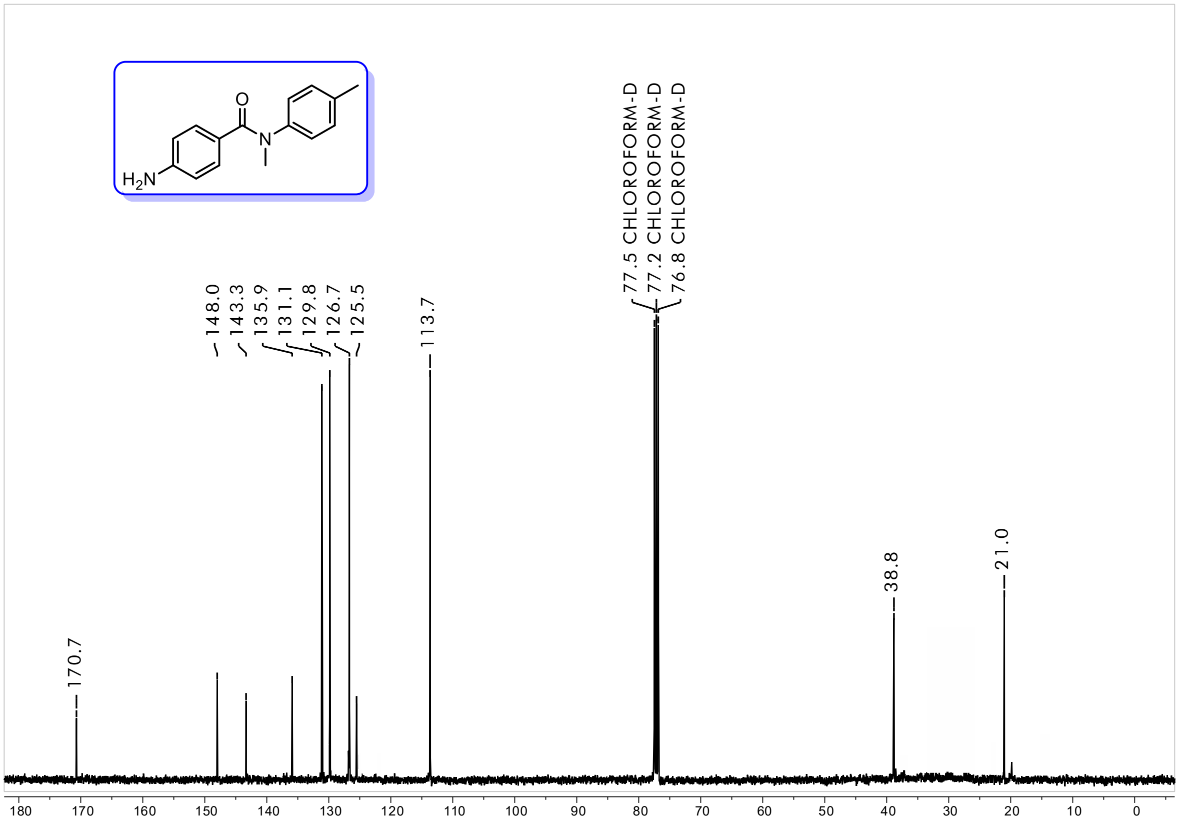

Spectrum 5. ^{13}C-NMR of 4-amino-*N*-methyl-*N*-(p-tolyl)benzamide (6b).

Description:
Ionization Mode:ESI+
History:Determine m/z[Peak Detect[Centroid,30,Area];Correct Base[5.0%]];Correct Base[5.0%];Average(MS[1] 1..1)

Mass Calibration data:CAL_PEG_600
Created:1/21/2025 11:51:58 AM
Created by:AccuTOF

Charge number:1
Tolerance:5.00(ppm), 5.00 .. 15.00(mmu)
Unsaturation Number:-2.5 .. 200.0 (Fraction:Both)
Element:^{12}C:0 .. 15, ^{1}H:0 .. 22, ^{35}Cl:0 .. 1, ^{14}N:2 .. 2, ^{16}O:1 .. 1

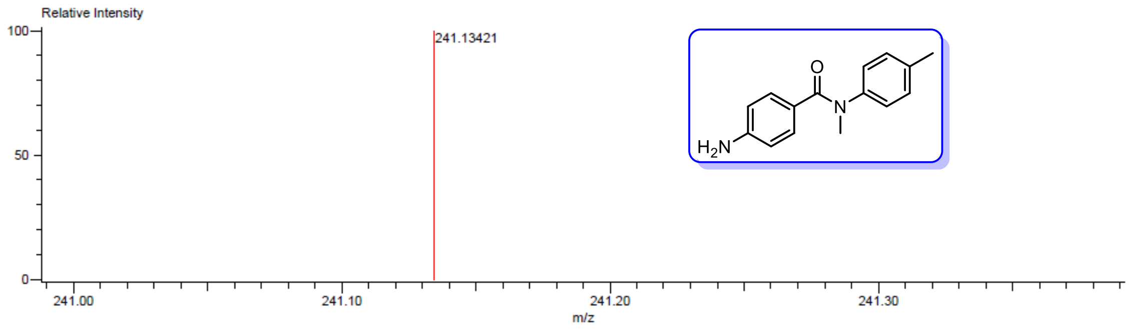

Mass	Intensity	Calc. Mass	Mass Difference (mmu)	Mass Difference (ppm)	Possible Formula	Unsaturation Number
241.13421	33647.02	241.13409	0.12	0.50	$^{12}C_{15}{}^{1}H_{17}{}^{14}N_2{}^{16}O_1$	8.5

Spectrum 6. HRMS of 4-amino-*N*-methyl-*N*-(p-tolyl)benzamide (6b).

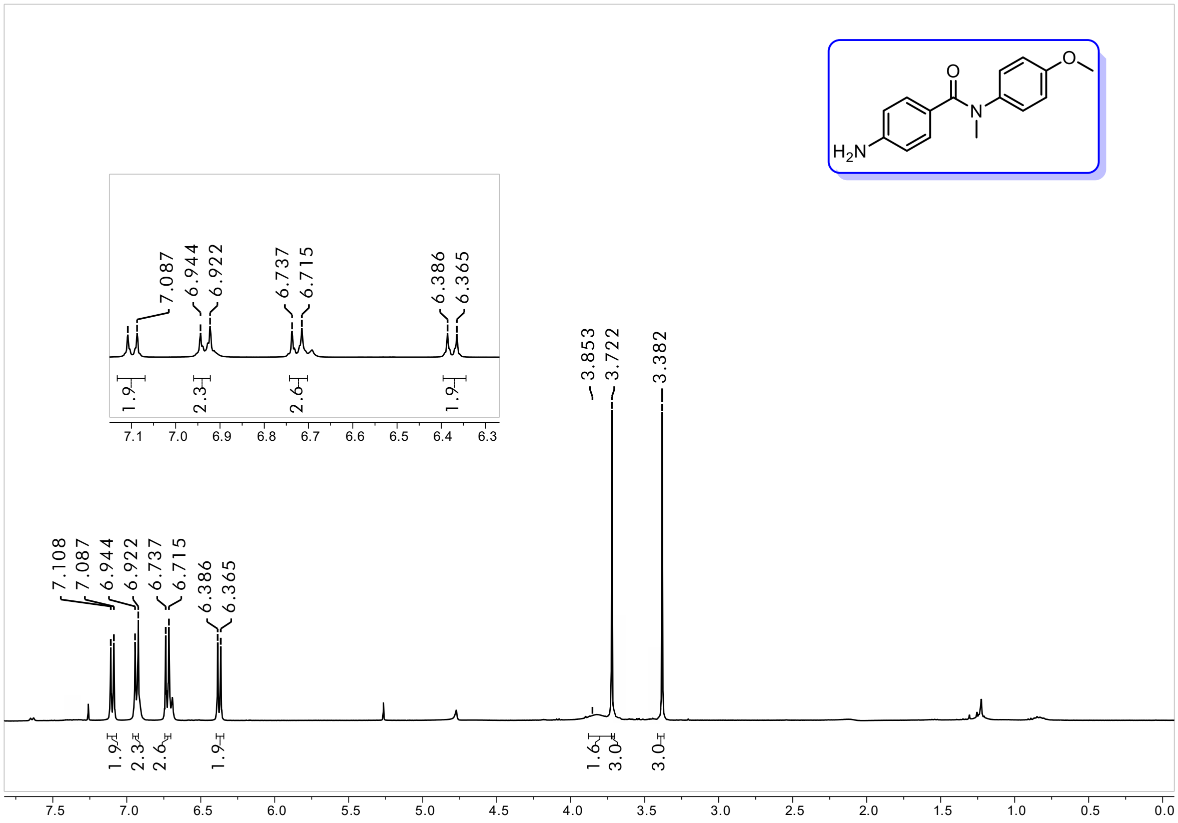

Spectrum 7. **^{1}H-NMR of 4-amino-*N*-(4-methoxyphenyl)-*N*-methylbenzamide (6c).**

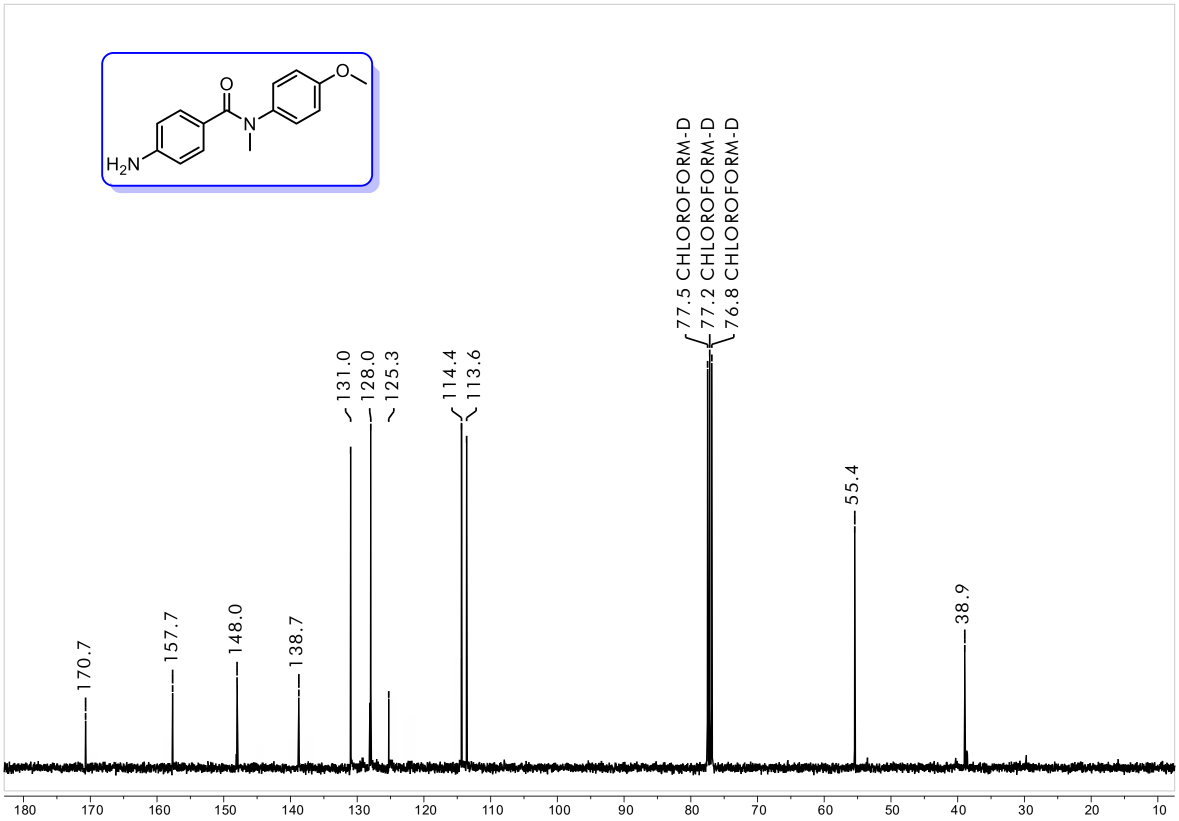

Spectrum 8. **^{13}C-NMR of 4-amino-*N*-(4-methoxyphenyl)-*N*-methylbenzamide (6c).**

Description:
Ionization Mode:ESI+
History:Determine m/z[Peak Detect[Centroid,30,Area];Correct Base[5.0%]];Correct Base[5.0%];Average(MS[1] 1..1)

Mass Calibration data:CAL_PEG_600
Created:2/5/2025 10:56:43 AM
Created by:AccuTOF

Charge number:1
Element:^{12}C:0 .. 15, ^{1}H:0 .. 30, ^{14}N:2 .. 2, ^{16}O:2 .. 2

Tolerance:200.00(ppm), 5.00 .. 15.00(mmu)

Unsaturation Number:-2.0 .. 100.0 (Fraction:Both)

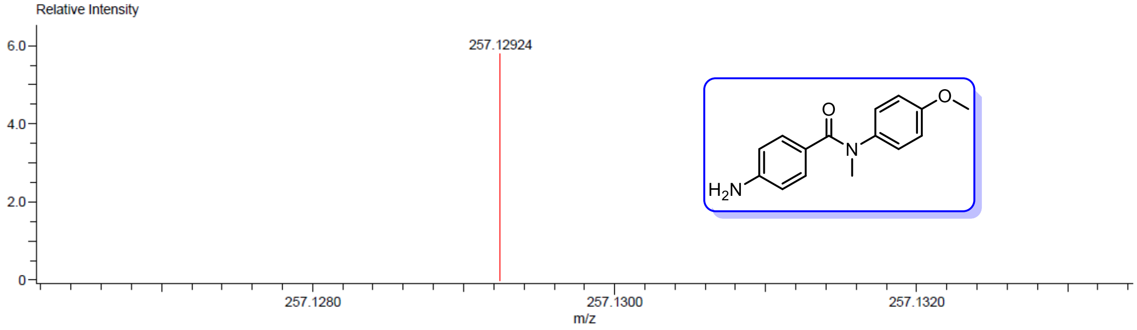

Mass	Intensity	Calc. Mass	Mass Difference (mmu)	Mass Difference (ppm)	Possible Formula	Unsaturation Number
257.12924	2678.08	257.12900	0.24	0.94	$^{12}C_{15}{}^{1}H_{17}{}^{14}N_{2}{}^{16}O_{2}$	8.5

Spectrum 9. HRMS of 4-amino-*N*-(4-methoxyphenyl)-*N*-methylbenzamide (6c).

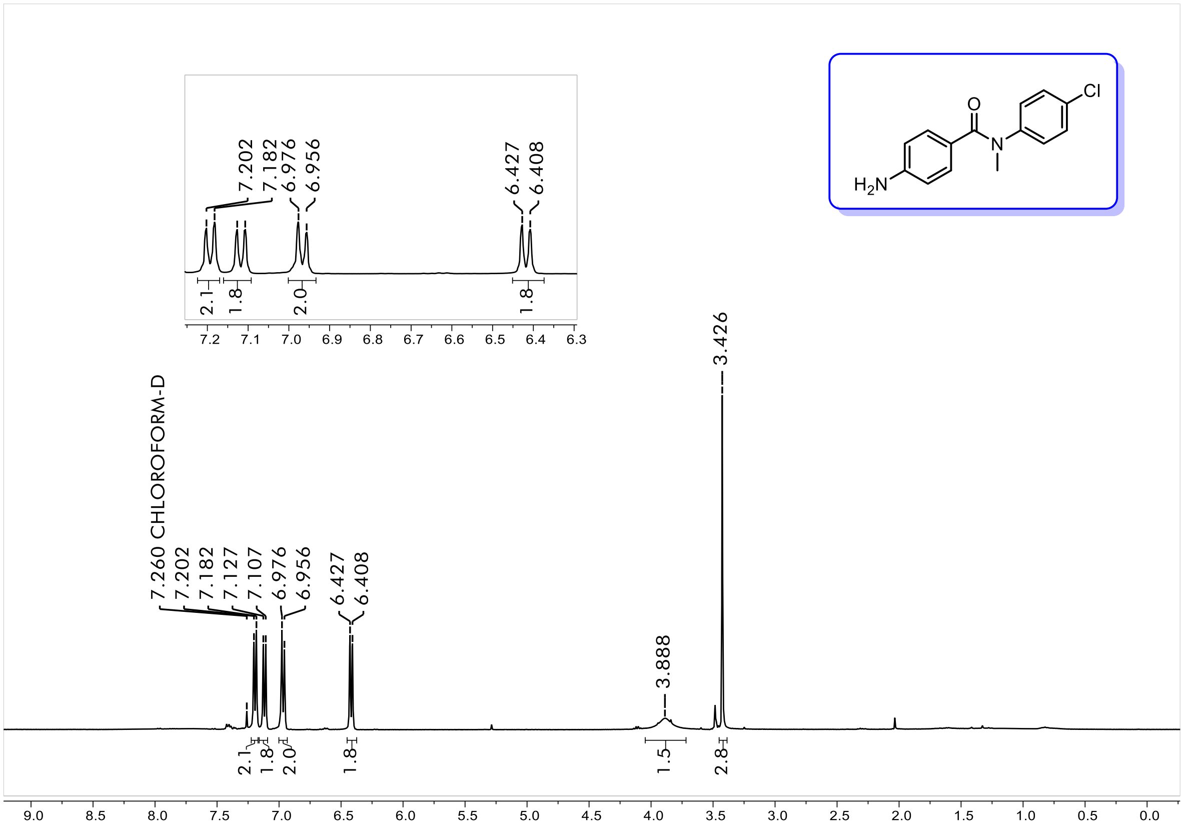

Spectrum 10. ^{1}H-NMR of 4-amino-*N*-(4-chlorophenyl)-*N*-methylbenzamide (6d).

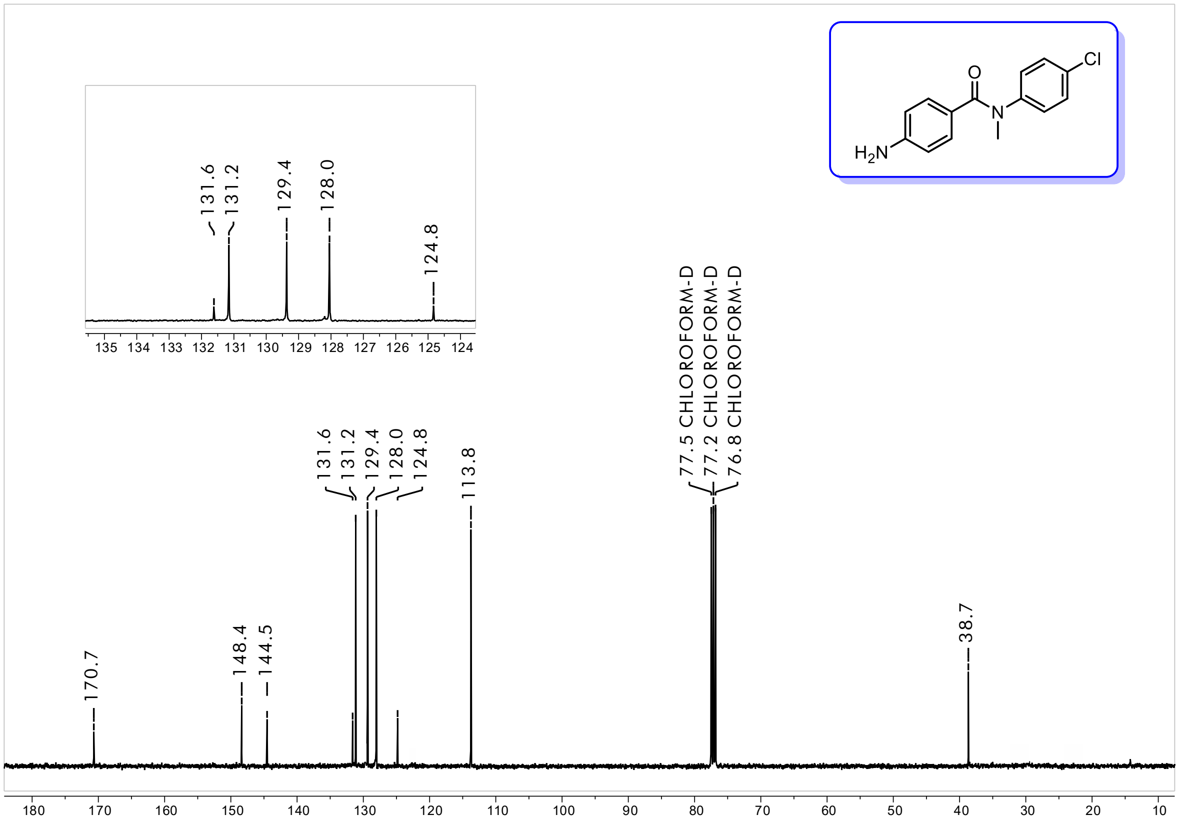

Spectrum 11. ^{13}C-NMR of 4-amino-*N*-(4-chlorophenyl)-*N*-methylbenzamide (6d).

Description:
Ionization Mode:ESI+
History:Determine m/z[Peak Detect[Centroid,30,Area];Correct Base[5.0%]];Correct Base[5.0%];Average(MS[1] 2..2)

Mass Calibration data:CAL_PEG_600
Created:1/21/2025 11:46:53 AM
Created by:AccuTOF

Charge number:1
Tolerance:5.00(ppm), 5.00 .. 15.00(mmu)
Unsaturation Number:-2.5 .. 200.0 (Fraction:Both)
Element:^{12}C:0 .. 14, ^{1}H:0 .. 22, ^{35}Cl:0 .. 1, ^{14}N:2 .. 2, ^{16}O:1 .. 1

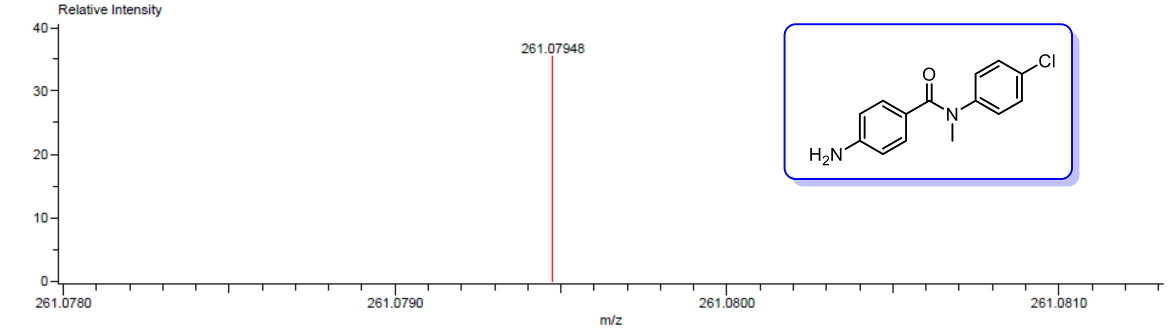

Mass	Intensity	Calc. Mass	Mass Difference (mmu)	Mass Difference (ppm)	Possible Formula	Unsaturation Number
261.07948	655.50	261.07947	0.01	0.04	$^{12}C_{14}{}^{1}H_{14}{}^{35}Cl_{1}{}^{14}N_{2}{}^{16}O_{1}$	8.5

Spectrum 12. HRMS of 4-amino-*N*-(4-chlorophenyl)-*N*-methylbenzamide (6d).

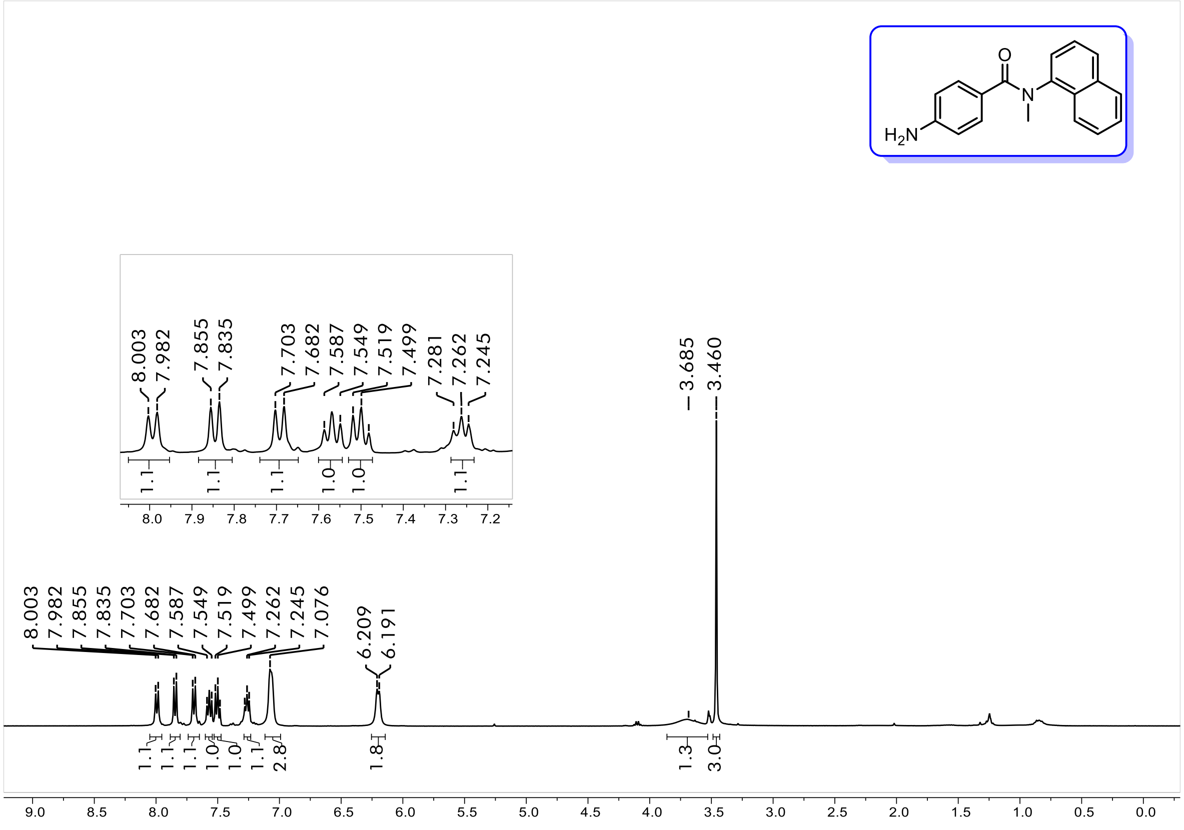

Spectrum 13. [1]**H-NMR of 4-amino-*N*-methyl-*N*-(naphthalen-1-yl)benzamide (6e).**

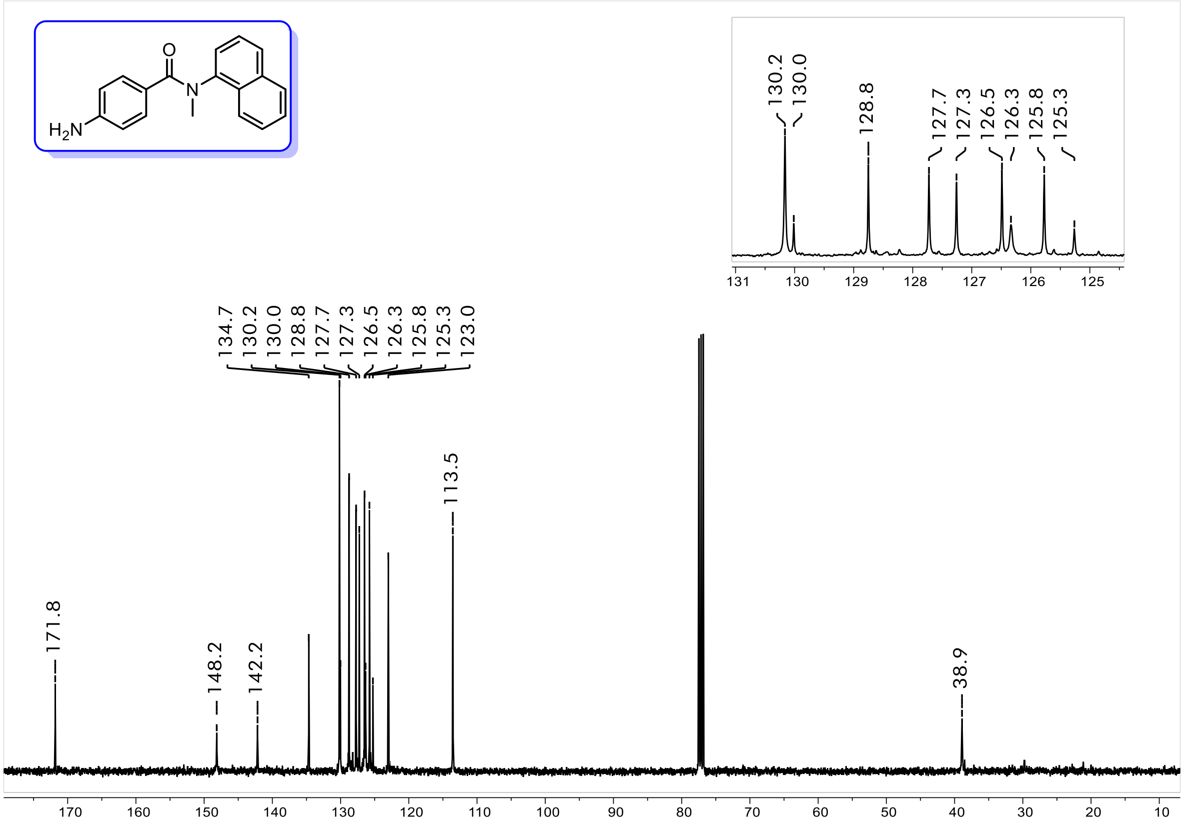

Spectrum 14. [13]**C-NMR of 4-amino-*N*-methyl-*N*-(naphthalen-1-yl)benzamide (6e).**

Description:
Ionization Mode:ESI+
History:Determine m/z[Peak Detect[Centroid,30,Area];Correct Base[5.0%]];Correct Base[5.0%];Average(MS[1] 1..1)

Mass Calibration data:CAL_PEG_600
Created:2/7/2025 1:20:43 PM
Created by:AccuTOF

Charge number:1
Element:^{12}C:0 .. 18, ^{1}H:0 .. 30, ^{14}N:2 .. 2, ^{16}O:0 .. 1

Tolerance:2.00(ppm), 5.00 .. 15.00(mmu)

Unsaturation Number:-2.0 .. 200.0 (Fraction:Both)

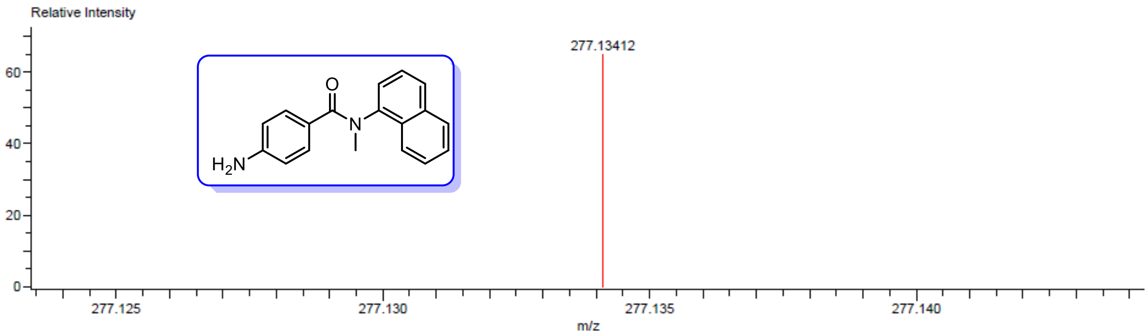

Mass	Intensity	Calc. Mass	Mass Difference (mmu)	Mass Difference (ppm)	Possible Formula	Unsaturation Number
277.13412	3329.50	277.13409	0.03	0.11	$^{12}C_{18}{}^{1}H_{17}{}^{14}N_{2}{}^{16}O_{1}$	11.5

Spectrum 15. HRMS of 4-amino-*N*-methyl-*N*-(naphthalen-1-yl)benzamide (6e).

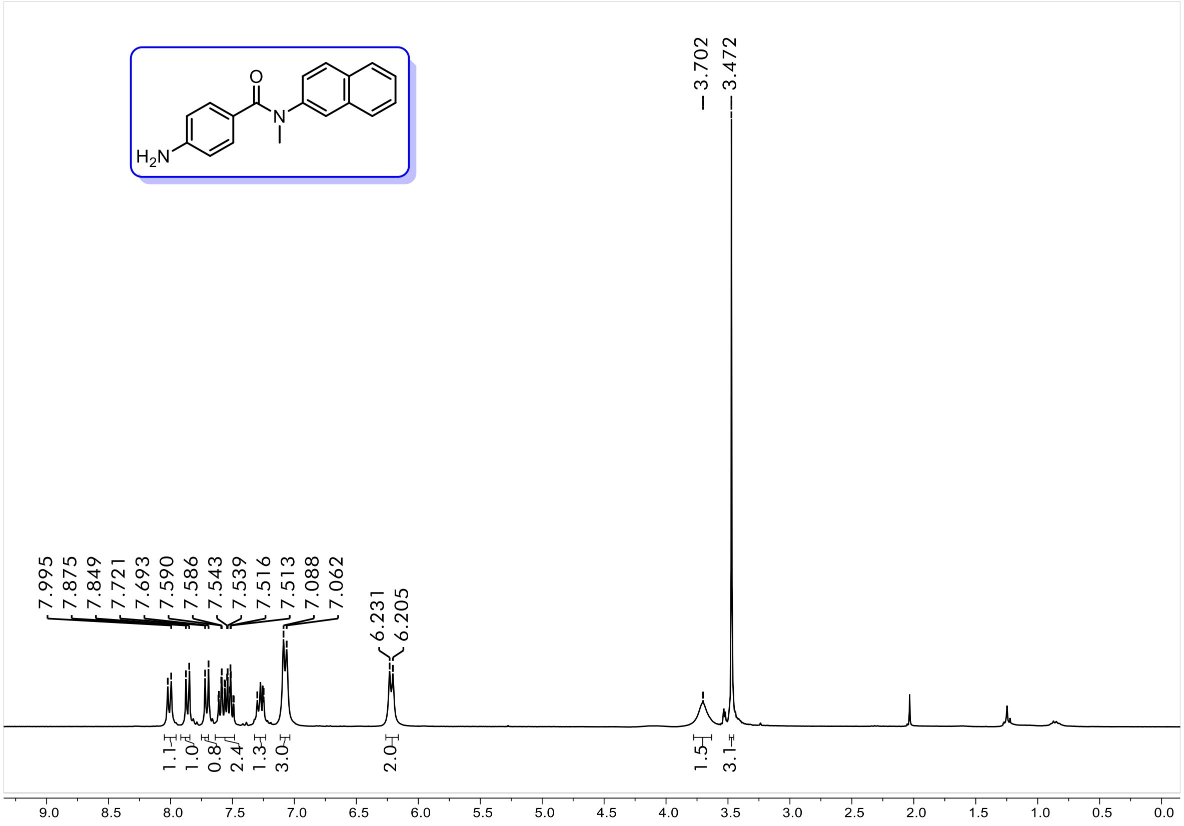

Spectrum 16. ^{1}H-NMR of 4-amino-N-methyl-N-(naphthalen-2-yl)benzamide (6f).

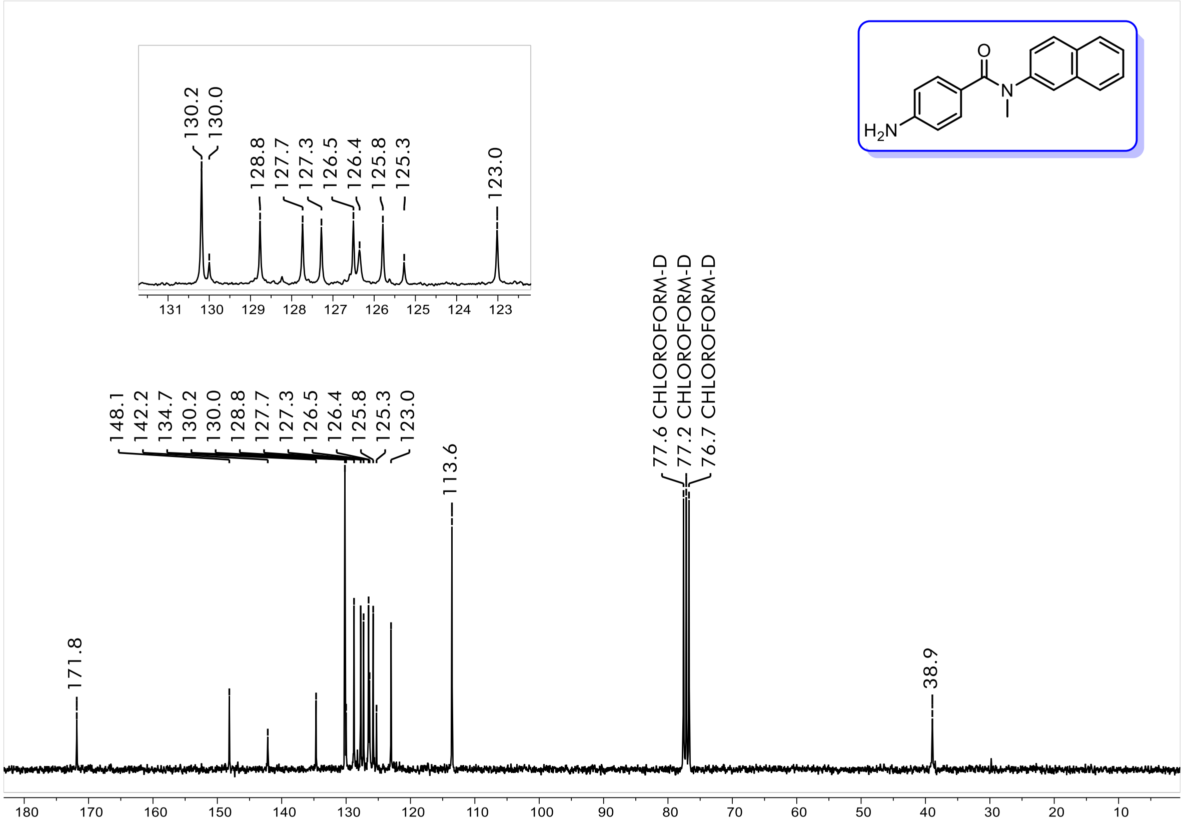

Spectrum 17. ^{13}C-NMR of 4-amino-N-methyl-N-(naphthalen-2-yl)benzamide (6f).

Description:
Ionization Mode:ESI+
History:Determine m/z[Peak Detect[Centroid,30,Area];Correct Base[5.0%]];Correct Base[5.0%];Average(MS[1] 0..0)

Mass Calibration data:CAL_PEG_600
Created:1/30/2025 1:54:24 PM
Created by:AccuTOF

Charge number:1
Tolerance:100.00(ppm), 5.00 .. 15.00(mmu)
Unsaturation Number:0.0 .. 100.0 (Fraction:Both)
Element:^{12}C:2 .. 18, ^{1}H:16 .. 17, ^{14}N:2 .. 2, ^{16}O:1 .. 1, ^{32}S:0 .. 0

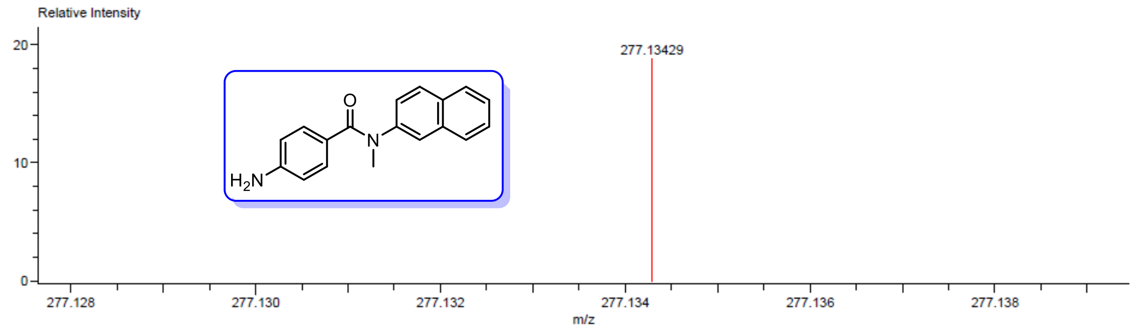

Mass	Intensity	Calc. Mass	Mass Difference (mmu)	Mass Difference (ppm)	Possible Formula	Unsaturation Number
277.13429	3025.31	277.13409	0.20	0.72	$^{12}C_{18}{}^{1}H_{17}{}^{14}N_{2}{}^{16}O_{1}$	11.5

Spectrum 18. HRMS of 4-amino-N-methyl-N-(naphthalen-2-yl)benzamide (6f).

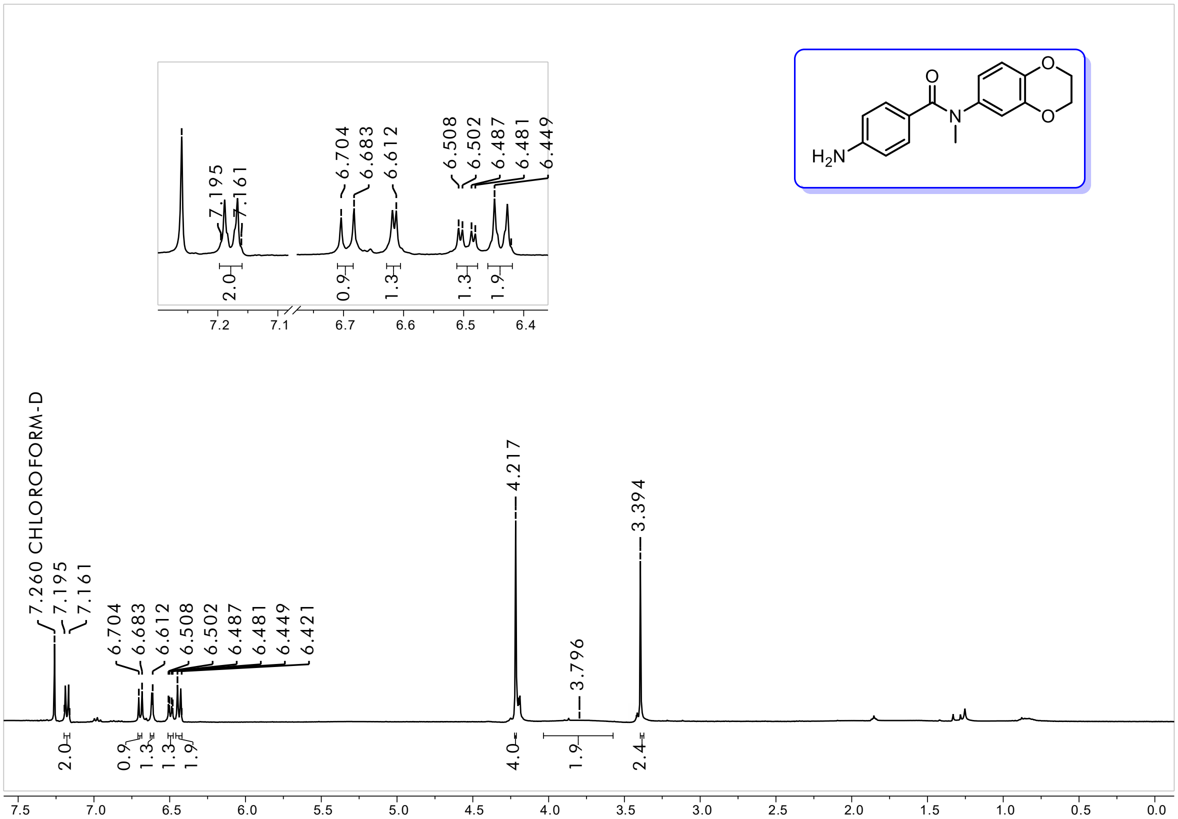

Spectrum 19. ^{1}H-NMR of 4-amino-*N*-(2,3-dihydrobenzo[*b*][1,4]dioxin-6-yl)-*N*-methylbenzamide (6g).

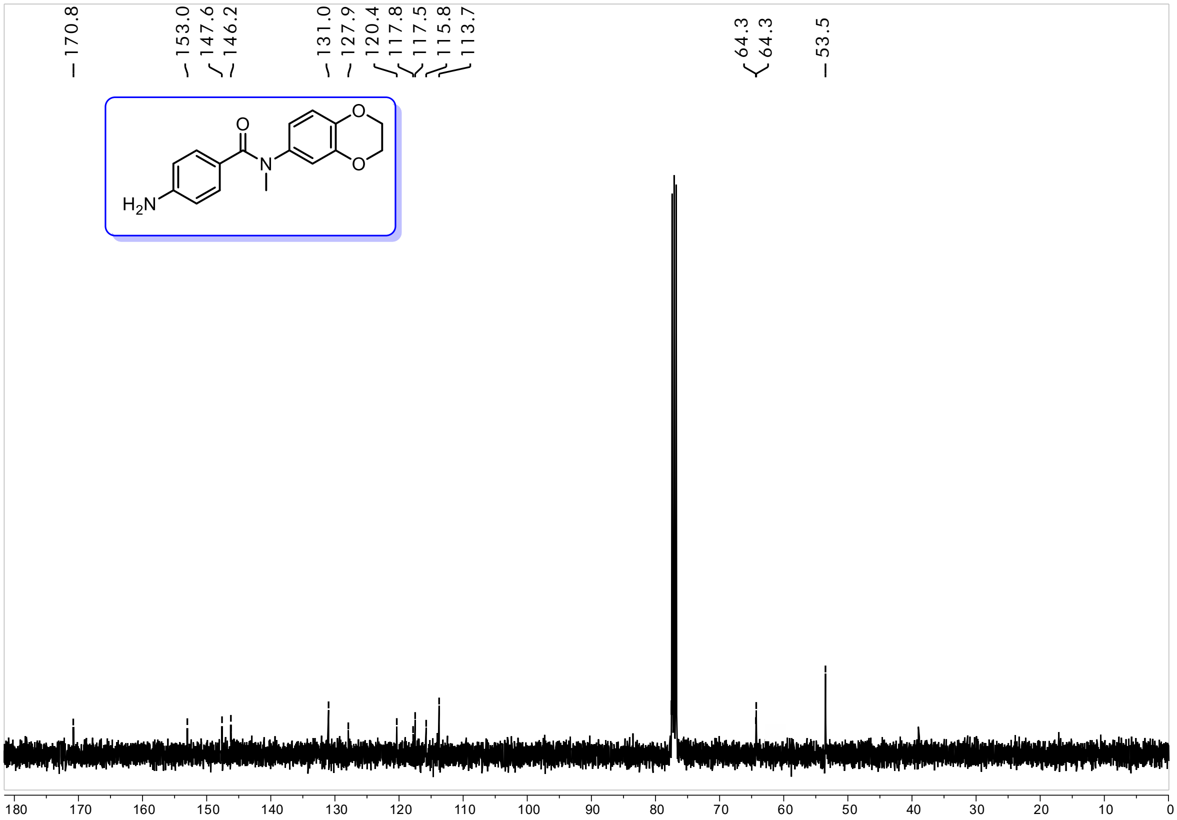

Spectrum 20. ^{13}C-NMR of 4-amino-*N*-(2,3-dihydrobenzo[*b*][1,4]dioxin-6-yl)-*N*-methylbenzamide (6g).

CHARACTERIZATION OF UREAS (SERIES 1–3)

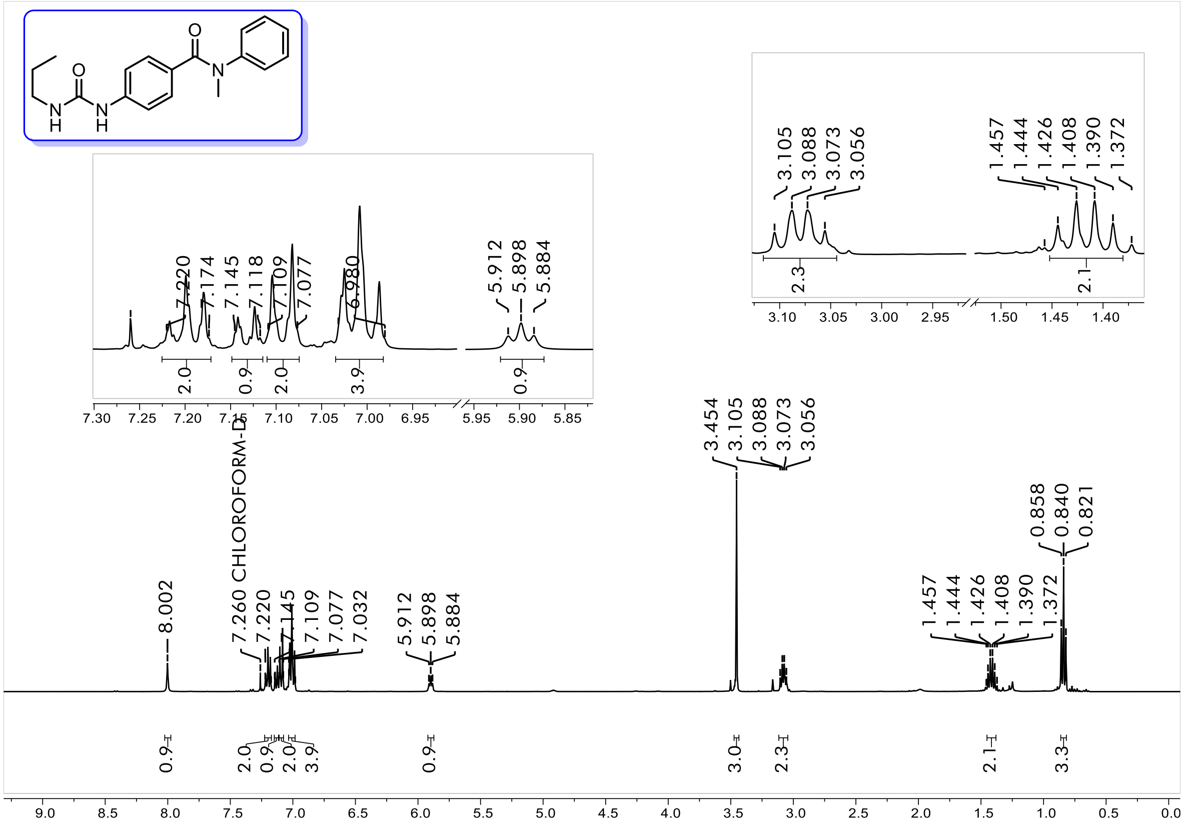

Spectrum 21. ^{1}H-NMR of *N*-methyl-*N*-phenyl-4-(3-propylureido)benzamide (1a).

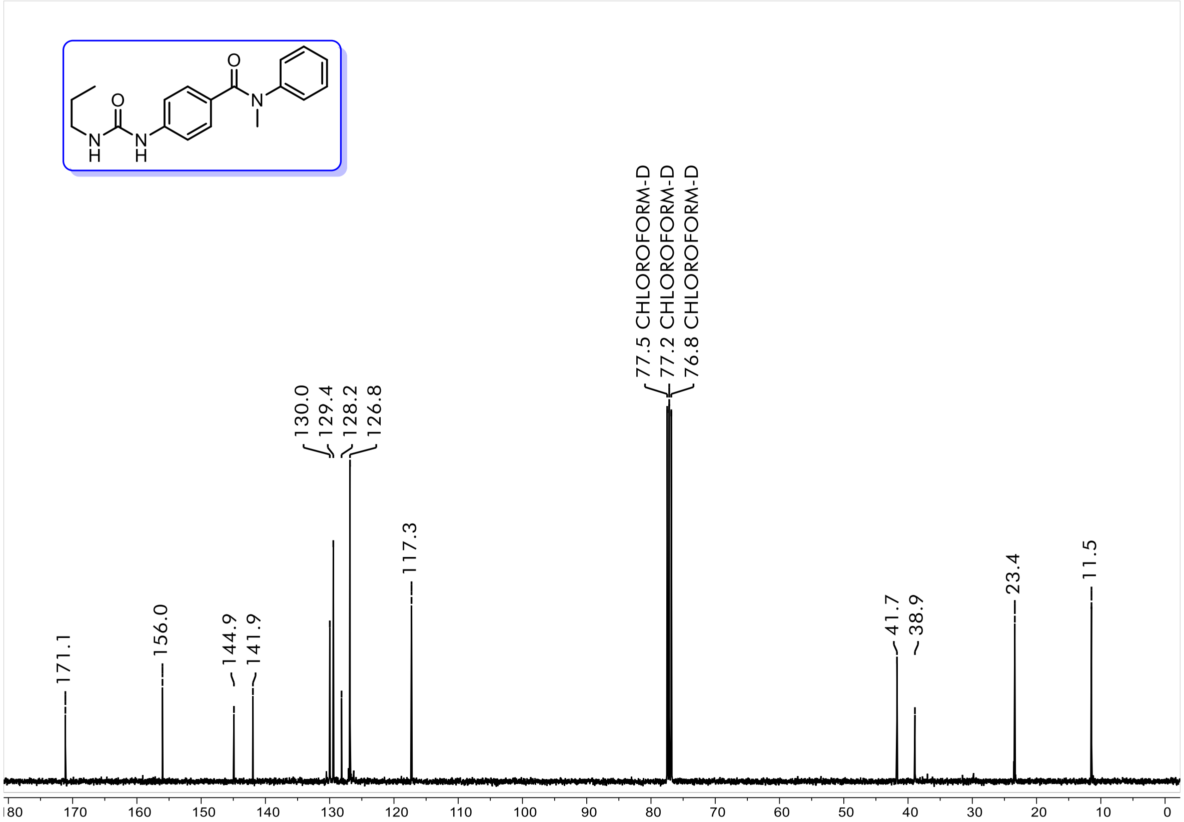

Spectrum 22. ^{13}C-NMR of *N*-methyl-*N*-phenyl-4-(3-propylureido)benzamide (1a).

Description:
Ionization Mode:ESI+
History:Determine m/z[Peak Detect[Centroid,30,Area];Correct Base[5.0%]];Correct Base[5.0%];Average(MS[1] 1..1)

Mass Calibration data:CAL_PEG_600
Created:1/21/2025 11:42:02 AM
Created by:AccuTOF

Charge number:1
Tolerance:5.00(ppm), 5.00 .. 15.00(mmu)
Unsaturation Number:-2.5 .. 200.0 (Fraction:Both)
Element:^{12}C:0 .. 18, ^{1}H:0 .. 22, ^{35}Cl:0 .. 1, ^{14}N:3 .. 3, ^{16}O:2 .. 2

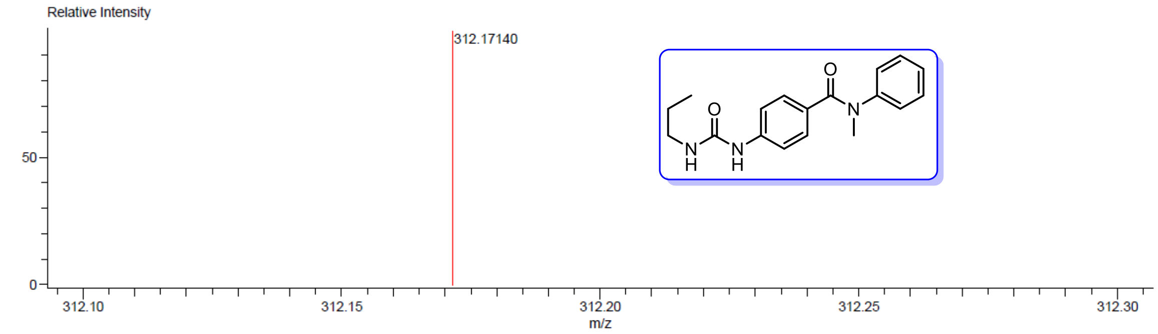

Mass	Intensity	Calc. Mass	Mass Difference (mmu)	Mass Difference (ppm)	Possible Formula	Unsaturation Number
312.17140	38103.75	312.17120	0.20	0.63	$^{12}C_{18}{}^{1}H_{22}{}^{14}N_3{}^{16}O_2$	9.5

Spectrum 23. HRMS of *N*-methyl-*N*-phenyl-4-(3-propylureido)benzamide (1a).

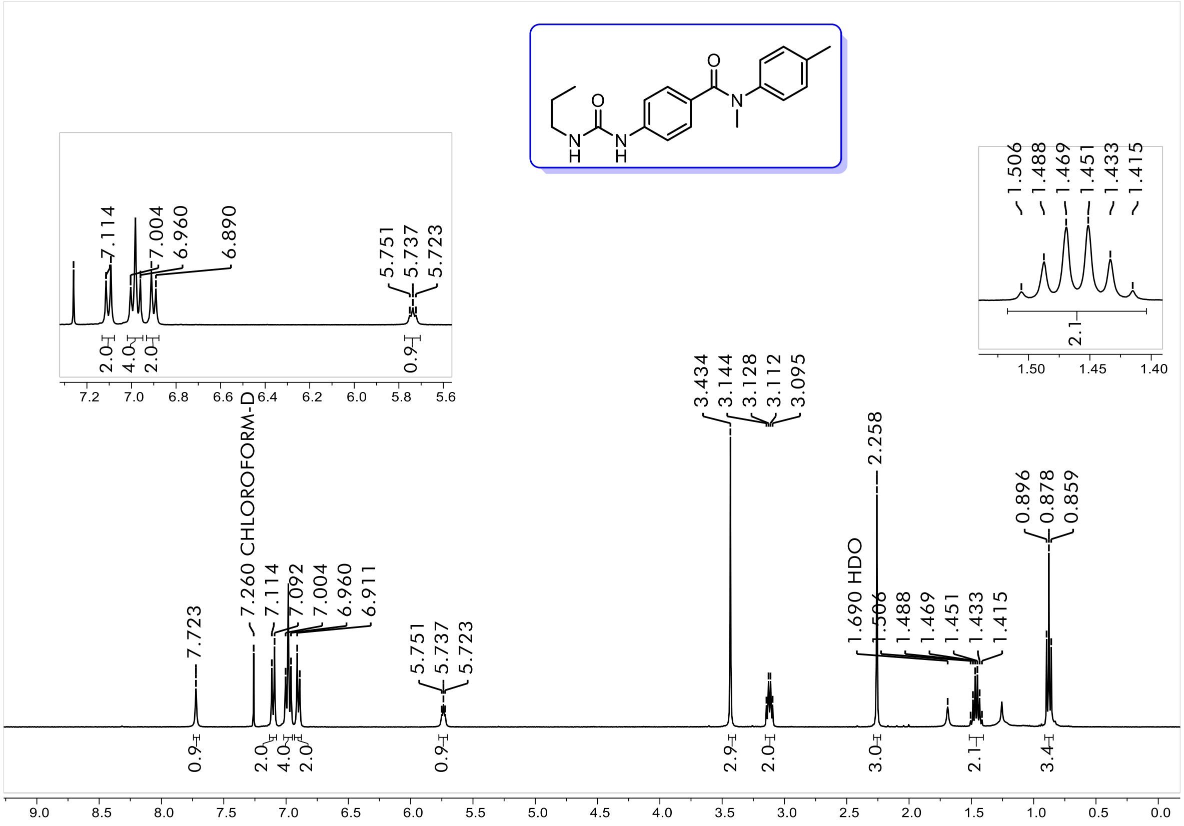

Spectrum 24. ^{1}H-NMR of *N*-methyl-4-(3-propylureido)-*N*-(*p*-tolyl)benzamide (1b).

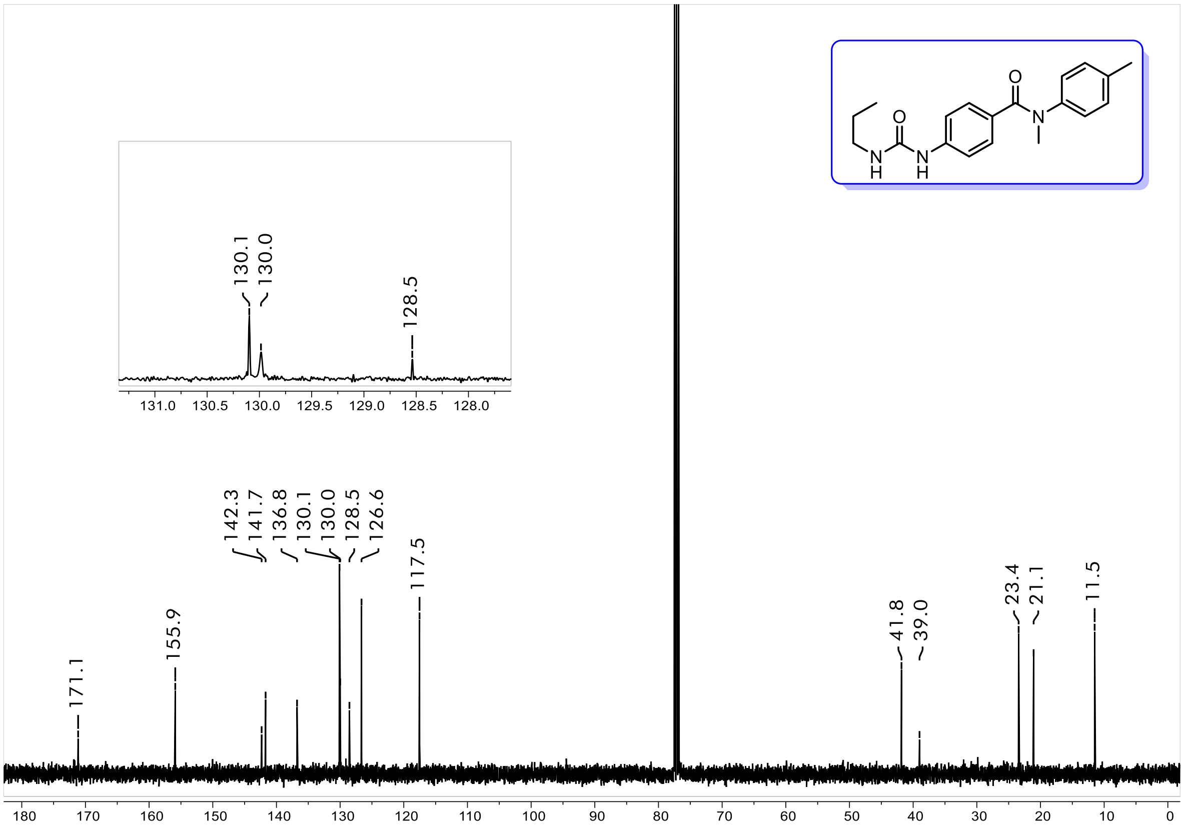

Spectrum 25. ^{13}C-NMR of *N*-methyl-4-(3-propylureido)-*N*-(*p*-tolyl)benzamide (1b).

Description:
Ionization Mode:ESI+
History:Determine m/z[Peak Detect[Centroid,30,Area];Correct Base[5.0%]];Correct Base[5.0%];Average(MS[1] 1..1)

Mass Calibration data:CAL_PEG_600
Created:1/27/2025 1:10:15 PM
Created by:AccuTOF

Charge number:1
Tolerance:1000.00(ppm), 5.00 .. 15.00(mmu)
Unsaturation Number:-2.5 .. 200.0 (Fraction:Both)
Element:^{12}C:0 .. 20, ^{1}H:0 .. 30, ^{35}Cl:0 .. 0, ^{14}N:3 .. 3, ^{16}O:2 .. 2

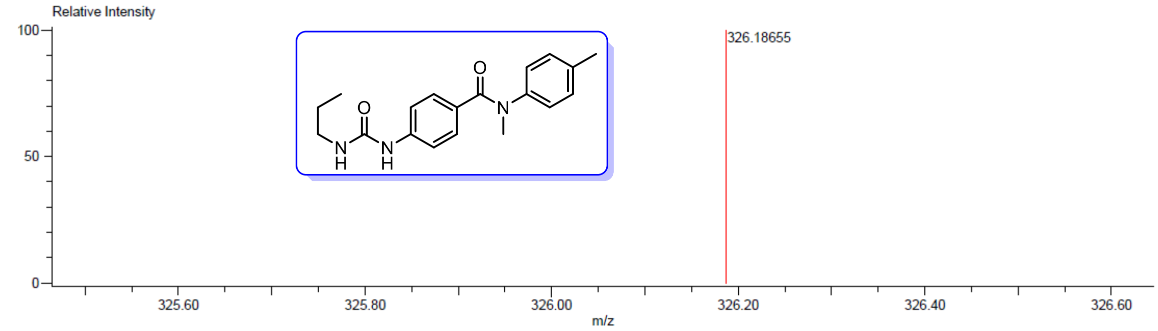

Mass	Intensity	Calc. Mass	Mass Difference (mmu)	Mass Difference (ppm)	Possible Formula	Unsaturation Number
326.18655	43137.25	326.18685	-0.30	-0.91	$^{12}C_{19}{}^{1}H_{24}{}^{14}N_{3}{}^{16}O_{2}$	9.5

Spectrum 26. HRMS of *N*-methyl-4-(3-propylureido)-*N*-(*p*-tolyl)benzamide (1b).

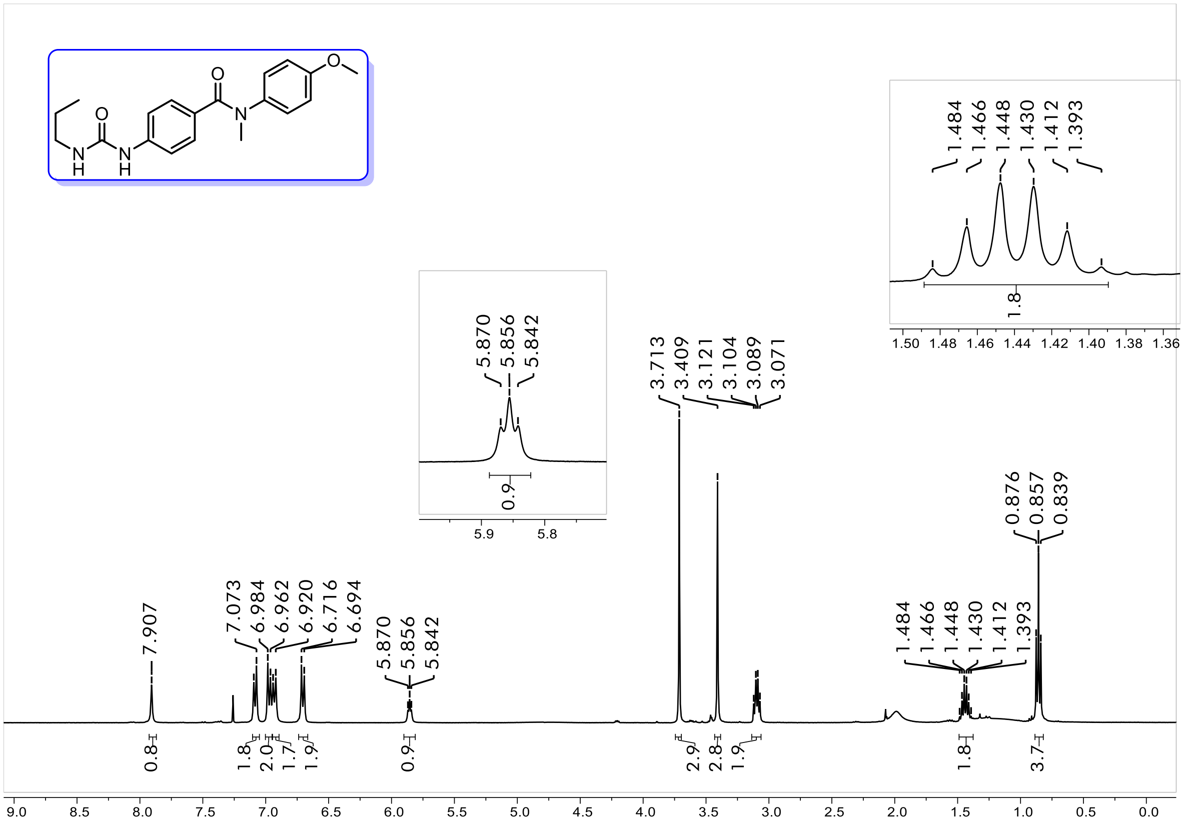

Spectrum 27. [1]**H-NMR of** ***N*****-(4-methoxyphenyl)-*****N*****-methyl-4-(3-propylureido)benzamide (1c).**

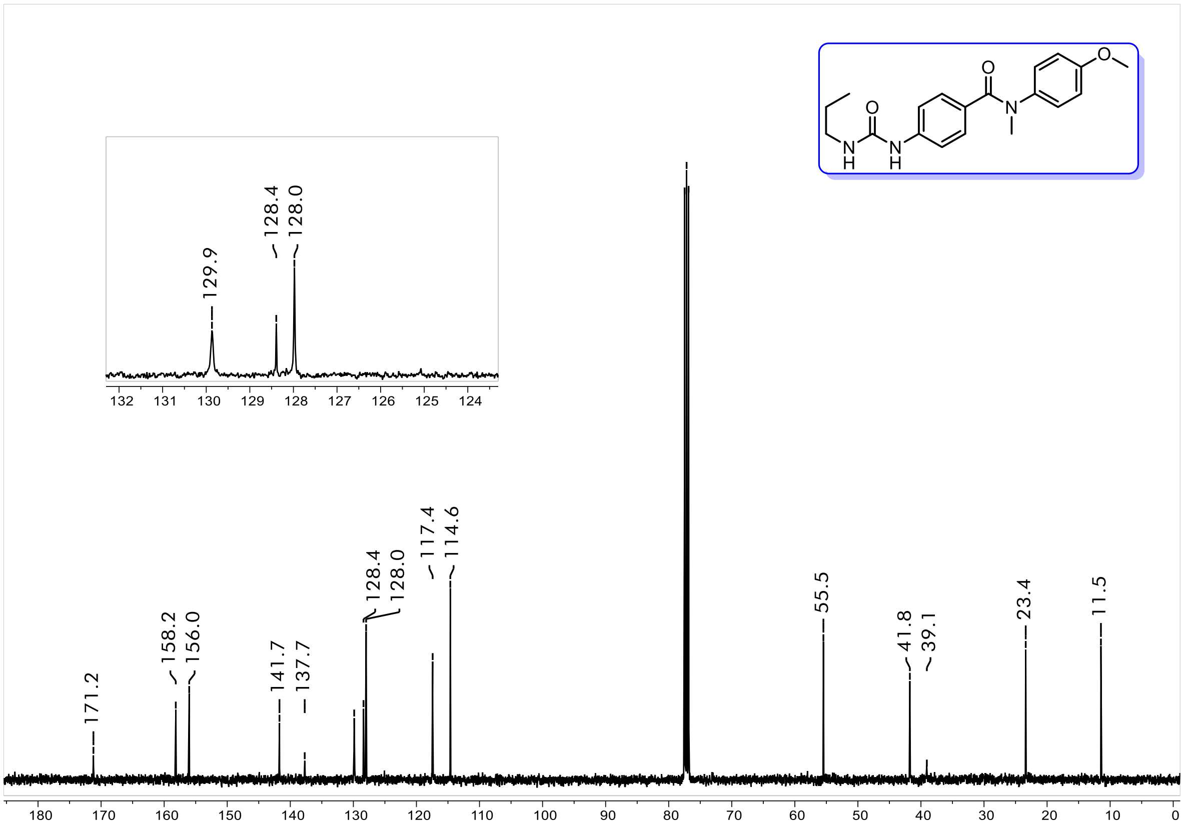

Spectrum 28. [13]**C-NMR of** ***N*****-(4-methoxyphenyl)-*****N*****-methyl-4-(3-propylureido)benzamide (1c).**

Description:
Ionization Mode:ESI+
History:Determine m/z[Peak Detect[Centroid,30,Area];Correct Base[5.0%]];Correct Base[5.0%];Average(MS[1] 1..1)

Mass Calibration data:CAL_PEG_600
Created:2/5/2025 10:49:06 AM
Created by:AccuTOF

Charge number:1
Element:^{12}C:0 .. 19, ^{1}H:0 .. 30, ^{14}N:0 .. 3, ^{16}O:3 .. 3

Tolerance:200.00(ppm), 5.00 .. 15.00(mmu)

Unsaturation Number:-2.0 .. 100.0 (Fraction:Both)

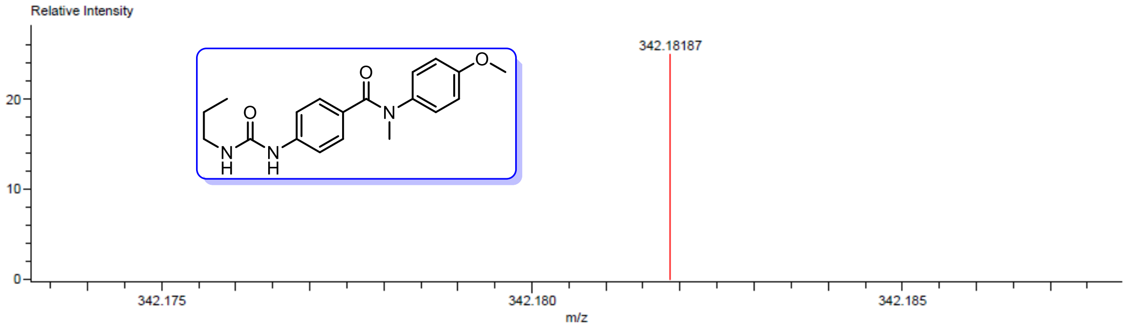

Mass	Intensity	Calc. Mass	Mass Difference (mmu)	Mass Difference (ppm)	Possible Formula	Unsaturation Number
342.18187	8107.68	342.18177	0.10	0.30	$^{12}C_{19}{}^{1}H_{24}{}^{14}N_{3}{}^{16}O_{3}$	9.5

Spectrum 29. HRMS of *N*-(4-methoxyphenyl)-*N*-methyl-4-(3-propylureido)benzamide (1c).

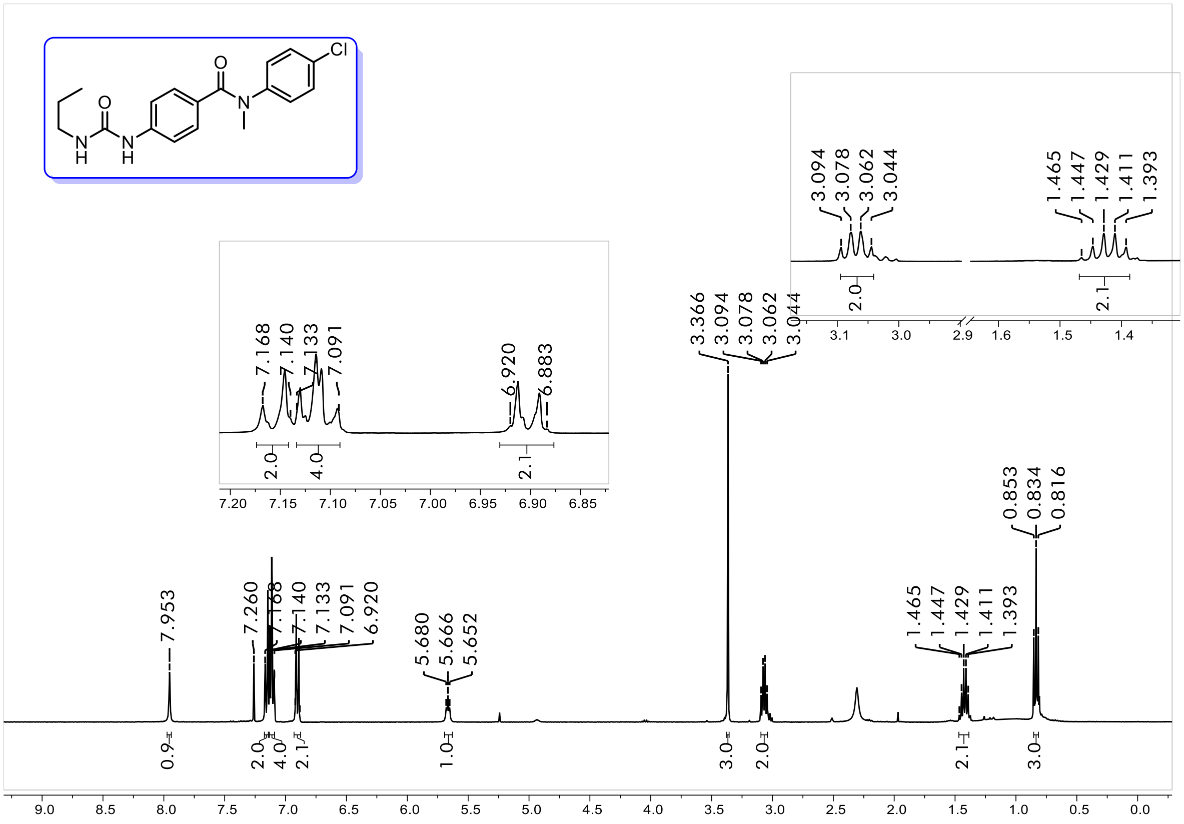

Spectrum 30. ^{1}H-NMR of *N*-(4-chlorophenyl)-*N*-methyl-4-(3-propylureido)benzamide (1d).

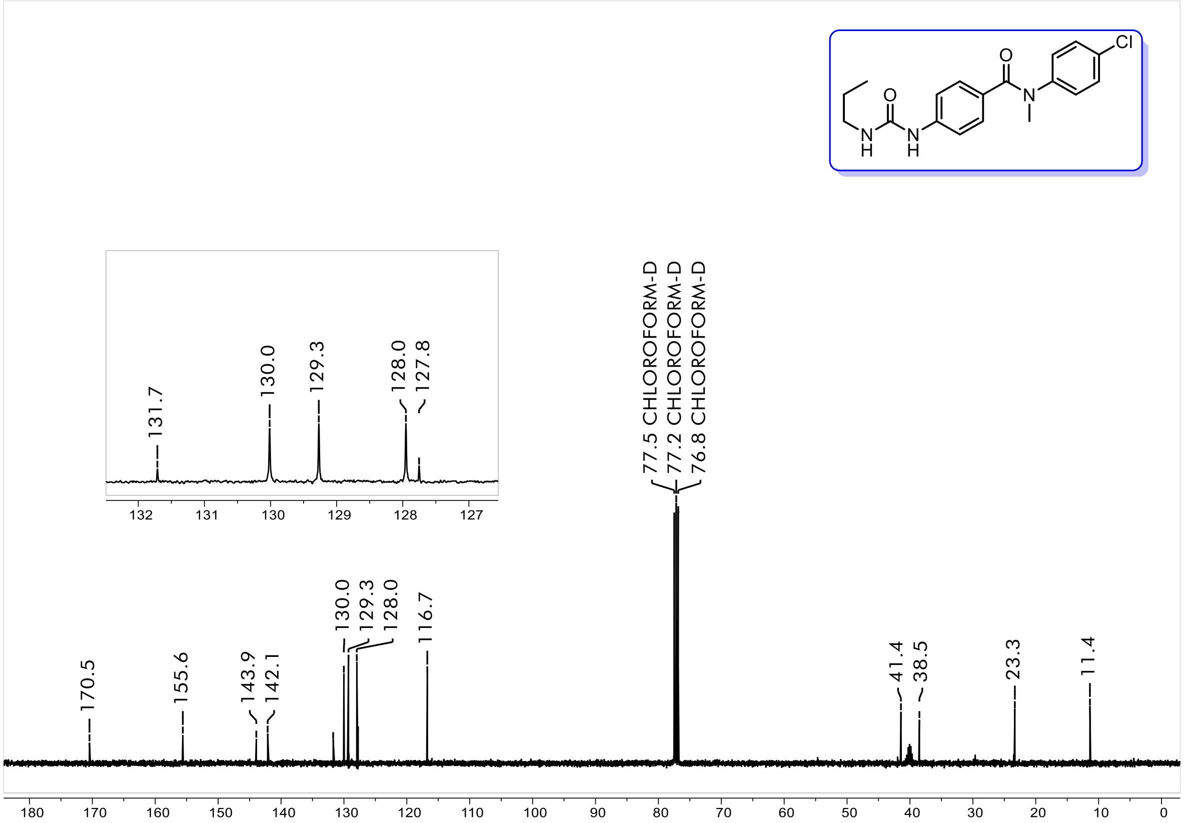

Spectrum 31. ^{13}C-NMR of *N*-(4-chlorophenyl)-*N*-methyl-4-(3-propylureido)benzamide (1d).

Description:
Ionization Mode:ESI+
History:Determine m/z[Peak Detect[Centroid,30,Area];Correct Base[5.0%]];Correct Base[5.0%];Average(MS[1] 2..2)

Mass Calibration data:CAL_PEG_600
Created:1/23/2025 12:34:39 PM
Created by:AccuTOF

Charge number:1
Tolerance:3.00(ppm), 5.00 .. 15.00(mmu)
Unsaturation Number:-2.5 .. 200.0 (Fraction:Both)
Element:^{12}C:0 .. 18, ^{1}H:0 .. 24, ^{35}Cl:0 .. 1, ^{14}N:3 .. 3, ^{16}O:2 .. 2

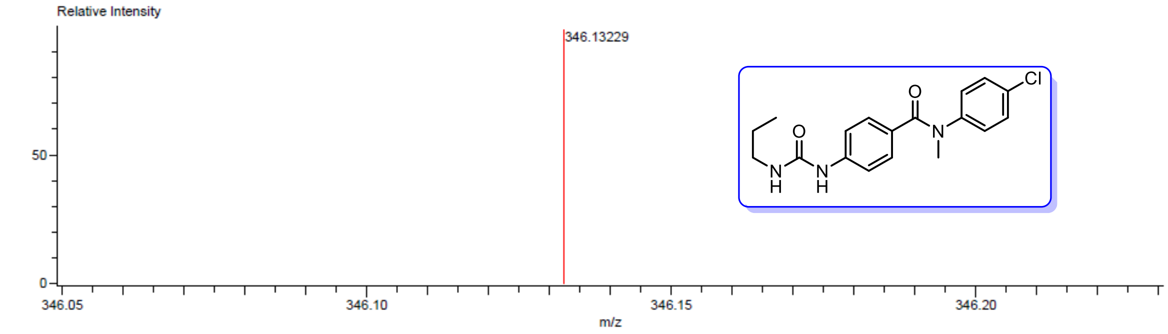

Mass	Intensity	Calc. Mass	Mass Difference (mmu)	Mass Difference (ppm)	Possible Formula	Unsaturation Number
346.13229	60613.25	346.13223	0.06	0.18	$^{12}C_{18}{}^{1}H_{21}{}^{35}Cl_{1}{}^{14}N_{3}{}^{16}O_{2}$	9.5

Spectrum 32. HRMS of *N*-(4-chlorophenyl)-*N*-methyl-4-(3-propylureido)benzamide (1d).

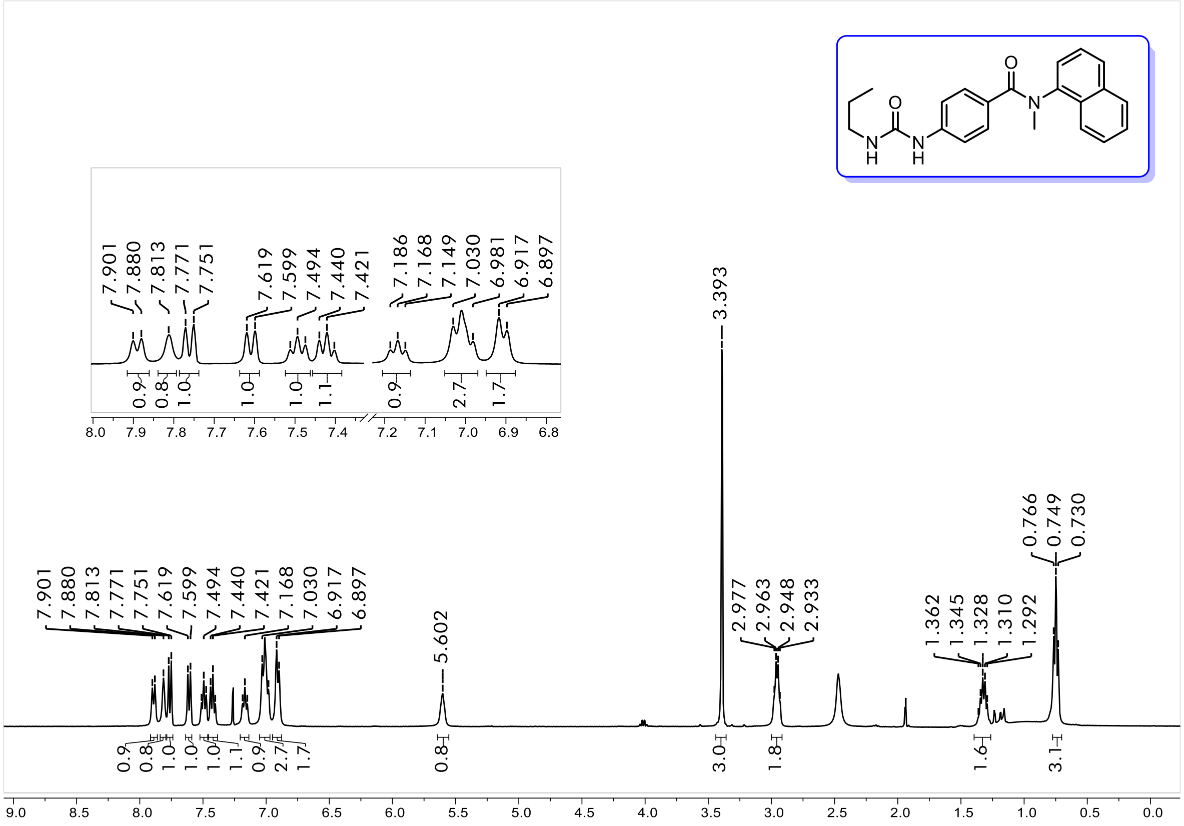

Spectrum 33. ^{1}H-NMR of -methyl-*N*-(naphthalen-1-yl)-4-(3-propylureido)benzamide (1e).

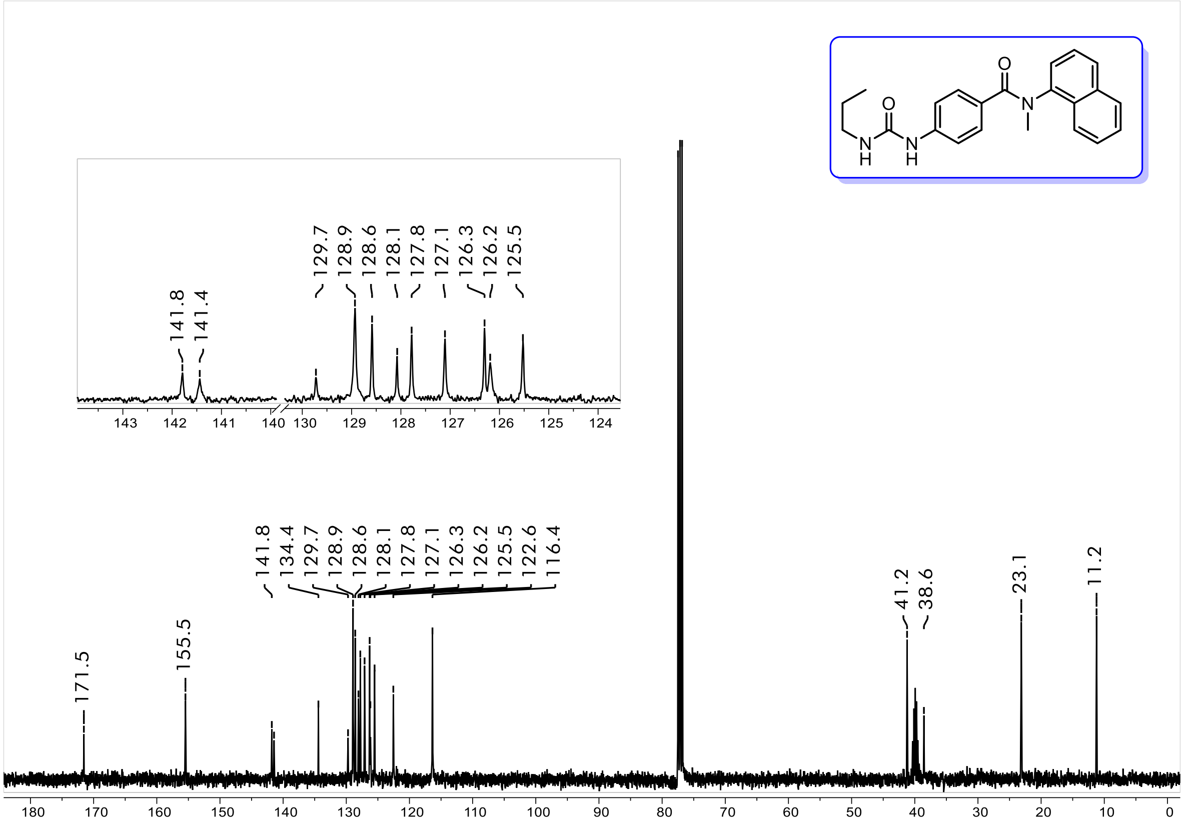

Spectrum 34. ^{13}C-NMR of -methyl-*N*-(naphthalen-1-yl)-4-(3-propylureido)benzamide (1e).

Description:
Ionization Mode:ESI+
History:Determine m/z[Peak Detect[Centroid,30,Area];Correct Base[5.0%]];Correct Base[5.0%];Average(MS[1] 3..3)

Mass Calibration data:CAL_PEG_600
Created:2/12/2025 11:36:13 AM
Created by:AccuTOF

Charge number:1
Element:^{12}C:0 .. 22, ^{1}H:0 .. 25, ^{14}N:3 .. 3, ^{16}O:0 .. 2

Tolerance:2.00(ppm), 5.00 .. 15.00(mmu)

Unsaturation Number:-2.0 .. 200.0 (Fraction:Both)

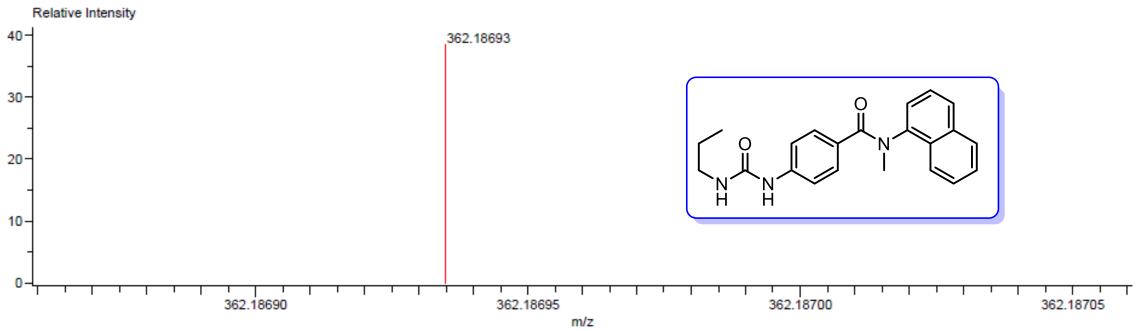

Mass	Intensity	Calc. Mass	Mass Difference (mmu)	Mass Difference (ppm)	Possible Formula	Unsaturation Number
362.18693	2348.11	362.18685	0.08	0.23	$^{12}C_{22}{}^{1}H_{24}{}^{14}N_{3}{}^{16}O_{2}$	12.5

Spectrum 35. HRMS of -methyl-*N*-(naphthalen-1-yl)-4-(3-propylureido)benzamide (1e).

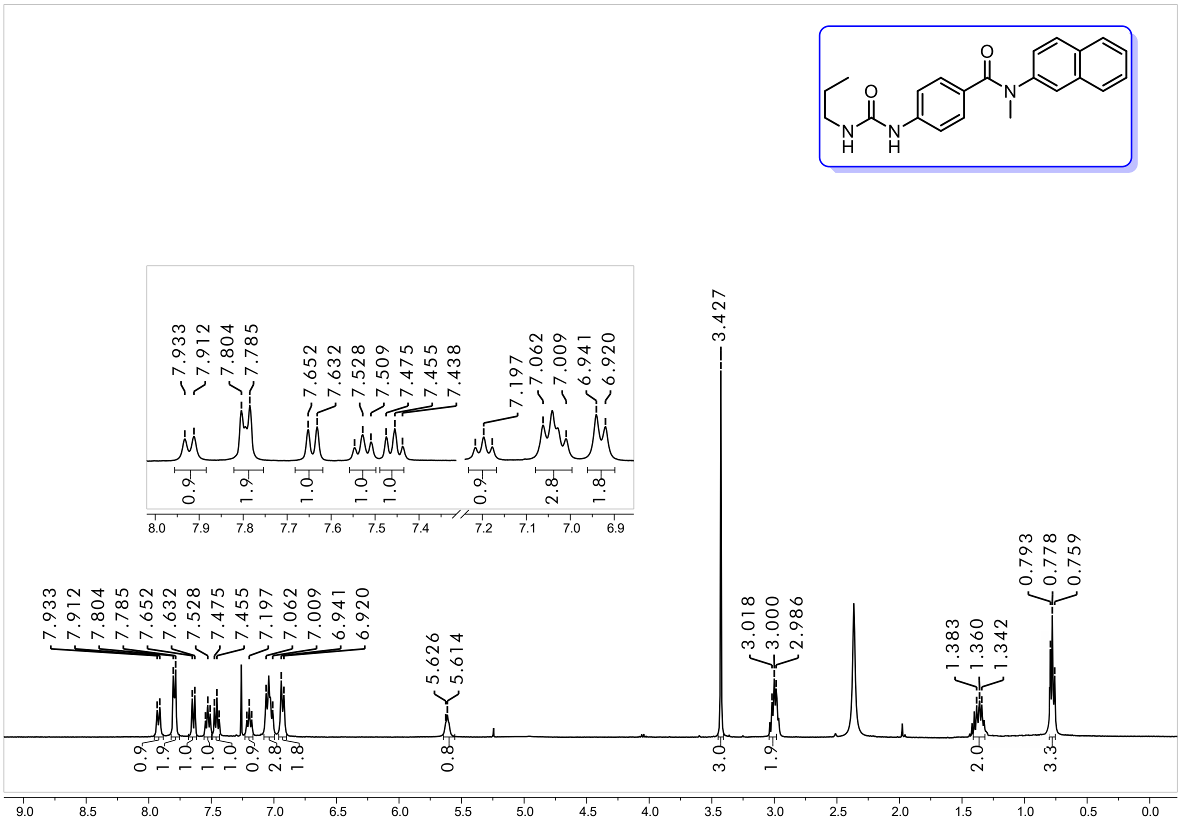

Spectrum 36. ^{1}H-NMR of *N*-methyl-*N*-(naphthalen-2-yl)-4-(3-propylureido)benzamide (1f).

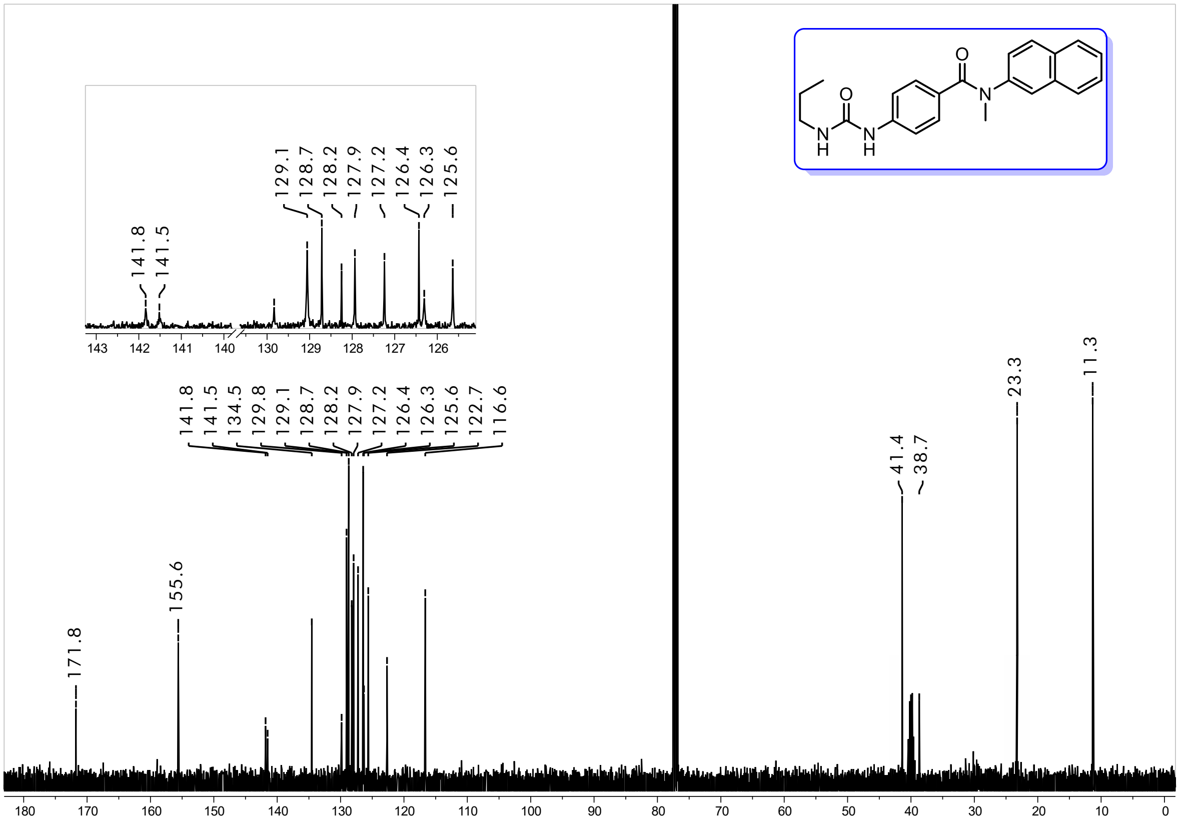

Spectrum 37. ^{13}C-NMR of *N*-methyl-*N*-(naphthalen-2-yl)-4-(3-propylureido)benzamide (1f).

Description:
Ionization Mode:ESI+
History:Determine m/z[Peak Detect[Centroid,30,Area];Correct Base[5.0%]];Correct Base[5.0%];Average(MS[1] 2..2)

Mass Calibration data:CAL_PEG_600
Created:2/5/2025 11:07:49 AM
Created by:AccuTOF

Charge number:1
Element:^{12}C:0 .. 22, ^{1}H:0 .. 24, ^{14}N:3 .. 3, ^{16}O:2 .. 2

Tolerance:200.00(ppm), 5.00 .. 15.00(mmu)

Unsaturation Number:-2.0 .. 100.0 (Fraction:Both)

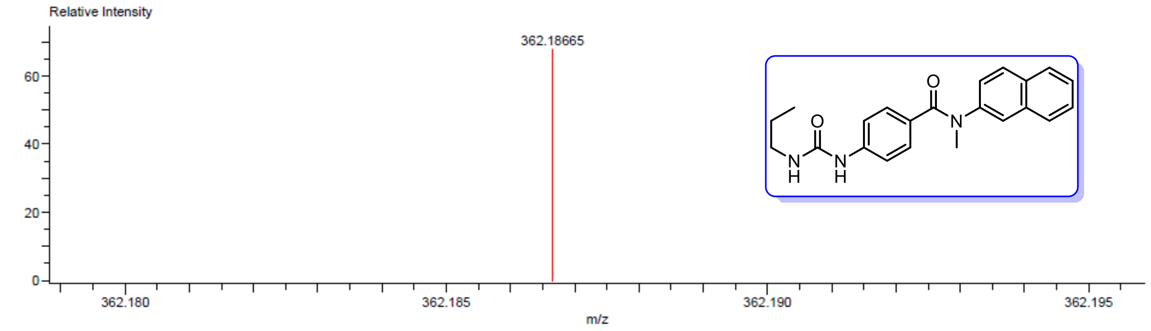

Mass	Intensity	Calc. Mass	Mass Difference (mmu)	Mass Difference (ppm)	Possible Formula	Unsaturation Number
362.18665	22686.79	362.18685	-0.20	-0.54	$^{12}C_{22}{}^{1}H_{24}{}^{14}N_{3}{}^{16}O_{2}$	12.5

Spectrum 38. HRMS of *N*-methyl-*N*-(naphthalen-2-yl)-4-(3-propylureido)benzamide (1f).

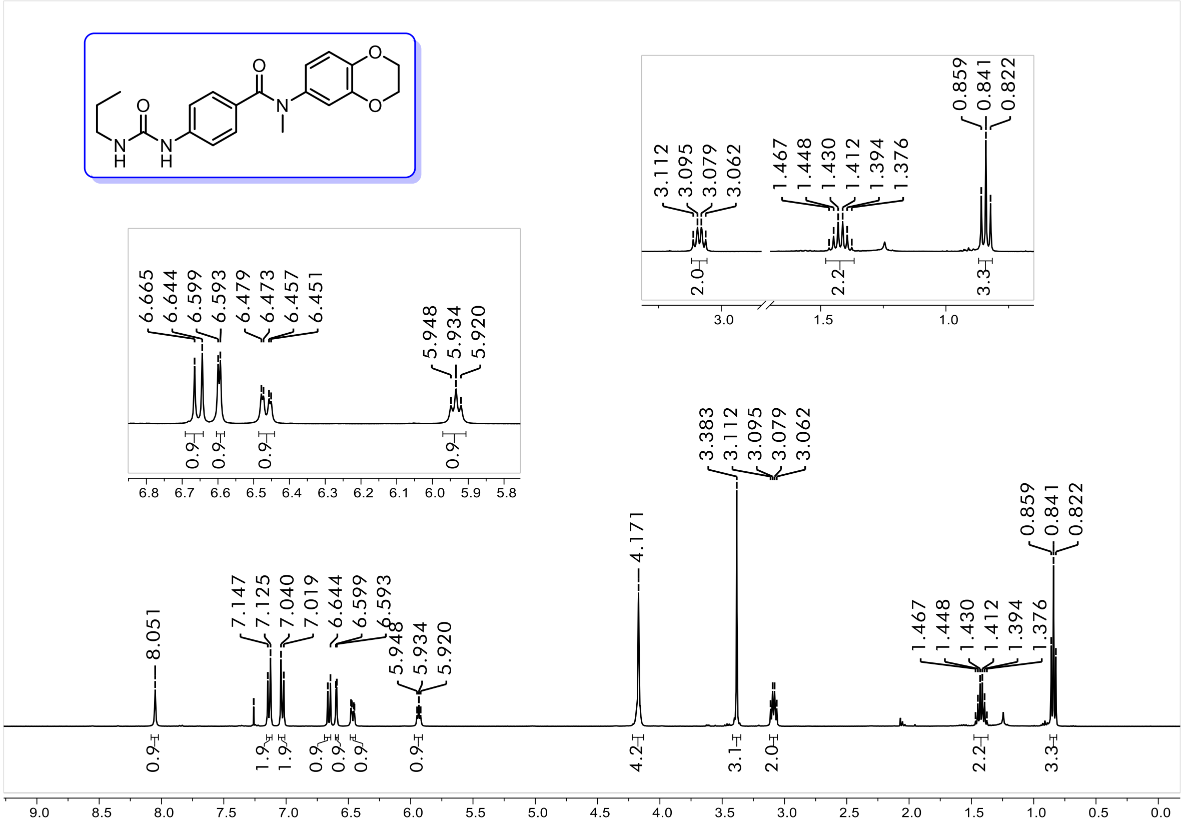

Spectrum 39. **^{1}H-NMR of *N*-(2,3-dihydrobenzo[b][1,4]dioxin-6-yl)-*N*-methyl-4-(3-propylureido)benzamide (1g).**

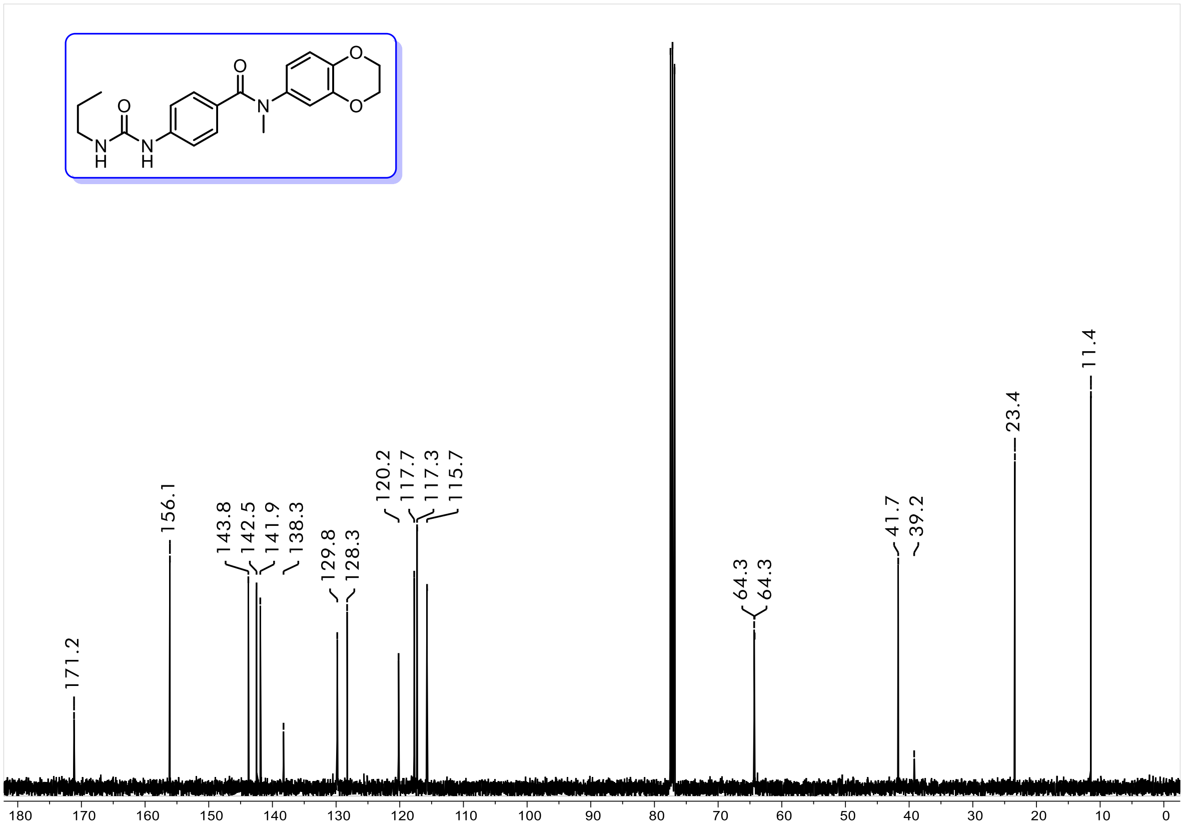

Spectrum 40. **^{13}C-NMR of *N*-(2,3-dihydrobenzo[b][1,4]dioxin-6-yl)-*N*-methyl-4-(3-propylureido)benzamide (1g).**

Instrument : MStation
Sample : 810 GGGI-38
Note : -
Inlet : Direct Ion Mode : EI+
RT : 1.40 min Scan# : (45,53)+(96,103)+(92,98)
Elements : C 24/0, H 49/0, N 4/1, O 5/2
Mass Tolerance : 1000ppm, 5mmu if m/z > 5
Unsaturation (U.S.) : -0.5 - 16.0

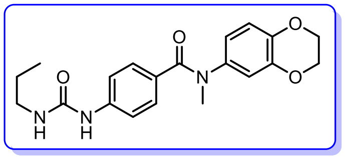

Observed m/z	Int%						
369.1697	100.00						

	Estimated m/z	Err[ppm / mmu]	U.S.	C	H	N	O
1	369.1689	+2.3 / +0.8	11.0	20	23	3	4

Spectrum 41. HRMS of *N*-(2,3-dihydrobenzo[b][1,4]dioxin-6-yl)-*N*-methyl-4-(3-propylureido)benzamide (1g).

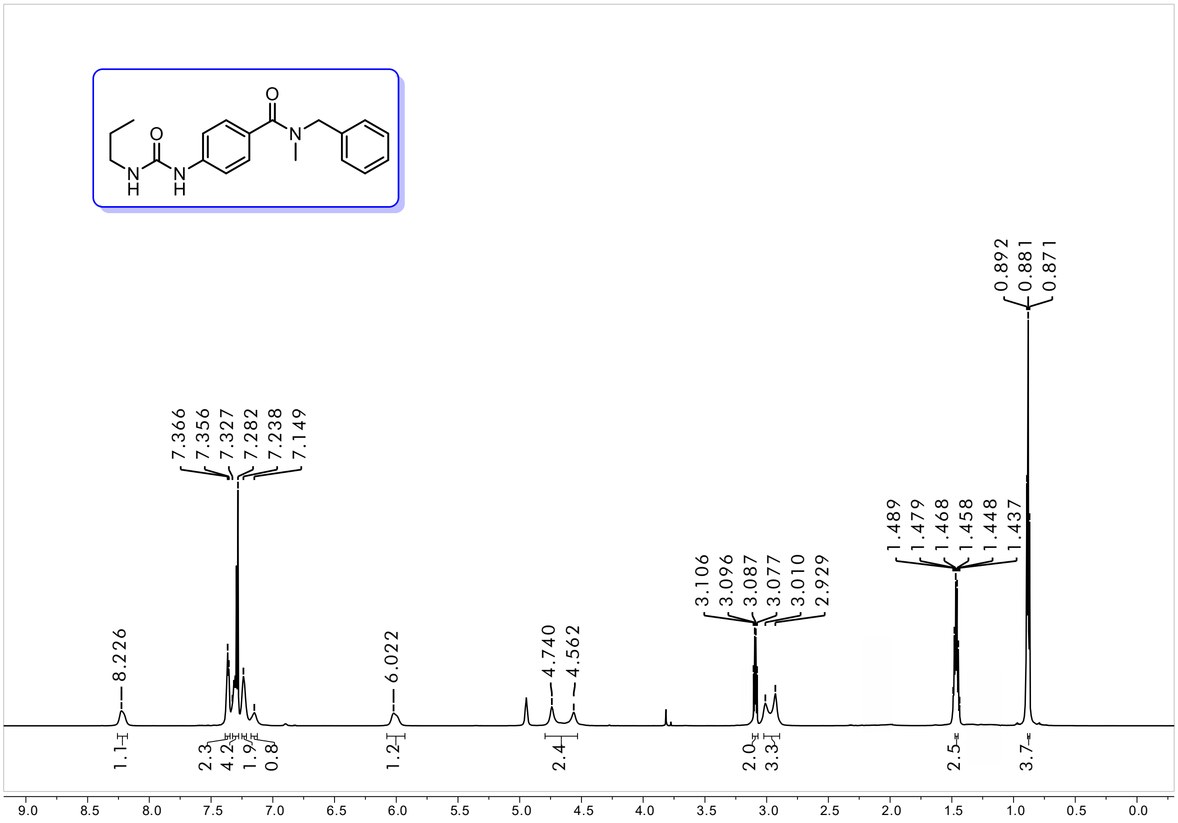

Spectrum 42. ^{1}**H-NMR of** ***N*****-benzyl-*****N*****-methyl-4-(3-propylureido)benzamide (2a, mixture of rotamers).**

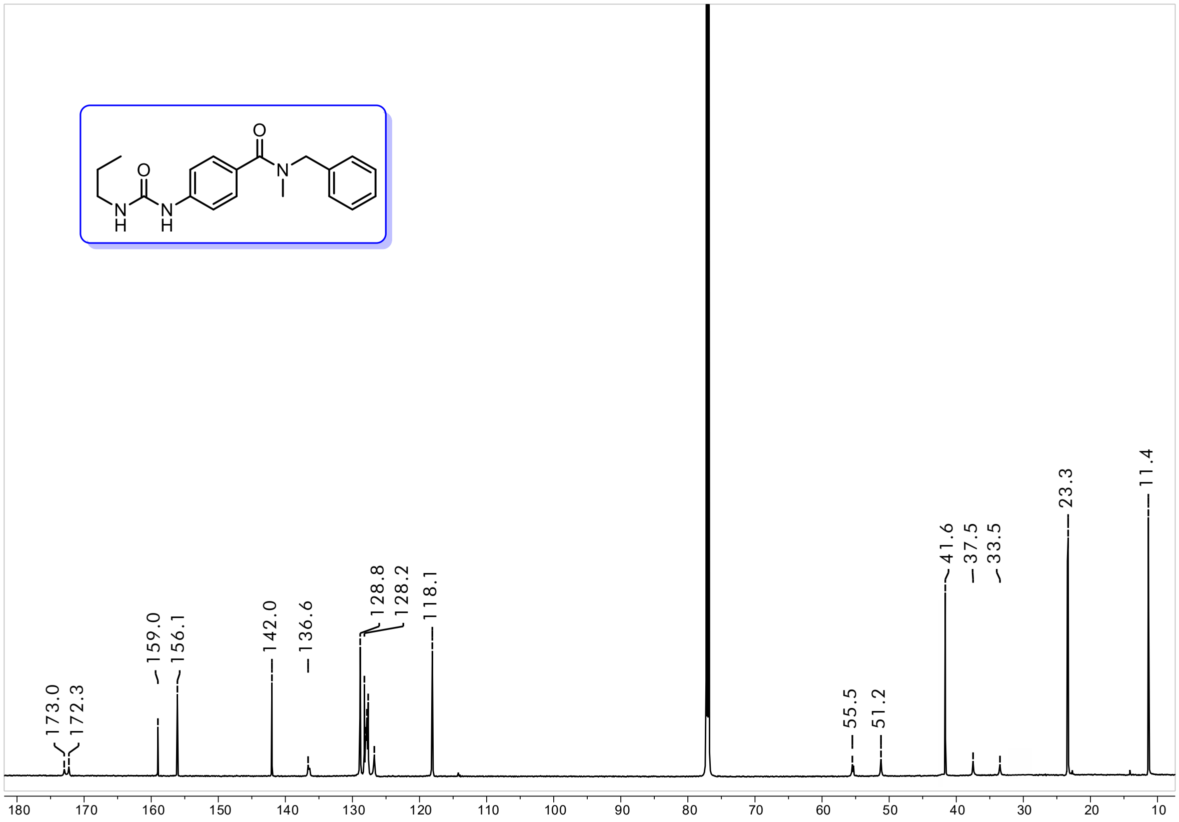

Spectrum 43. ^{13}**C-NMR of** ***N*****-benzyl-*****N*****-methyl-4-(3-propylureido)benzamide (2a, mixture of rotamers).**

Description:
Ionization Mode:ESI+
History:Determine m/z[Peak Detect[Centroid,30,Area];Correct Base[5.0%]];Correct Base[5.0%];Average(MS[1] 3..3)

Mass Calibration data:CAL_PEG_600
Created:4/24/2024 9:03:05 AM
Created by:

Charge number:1
Tolerance:100.00(ppm), 5.00 .. 15.00(mmu)
Unsaturation Number:-1.5 .. 100.0 (Fraction:Both)
Element:^{12}C:0 .. 19, ^{1}H:0 .. 60, ^{35}Cl:0 .. 0, ^{14}N:3 .. 3, ^{16}O:2 .. 2

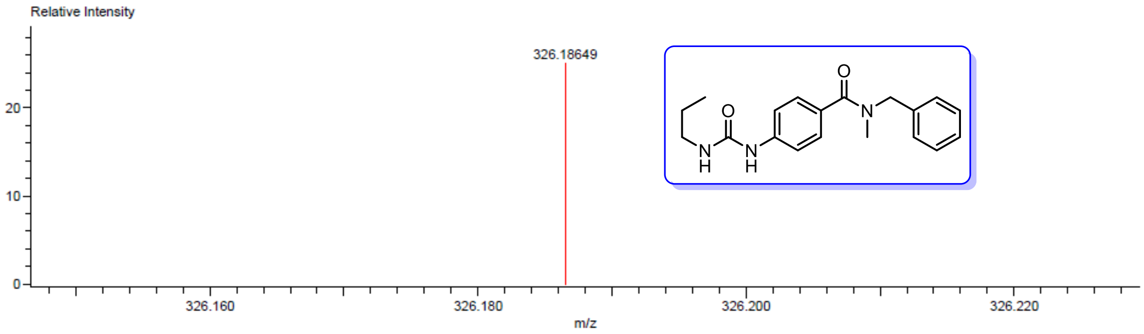

Mass	Intensity	Calc. Mass	Mass Difference (mmu)	Mass Difference (ppm)	Possible Formula	Unsaturation Number
326.18649	3900.14	326.18685	-0.36	-1.11	$^{12}C_{19}{}^{1}H_{24}{}^{14}N_3{}^{16}O_2$	9.5

Spectrum 44. HRMS of *N*-benzyl-*N*-methyl-4-(3-propylureido)benzamide (2a, mixture of rotamers).

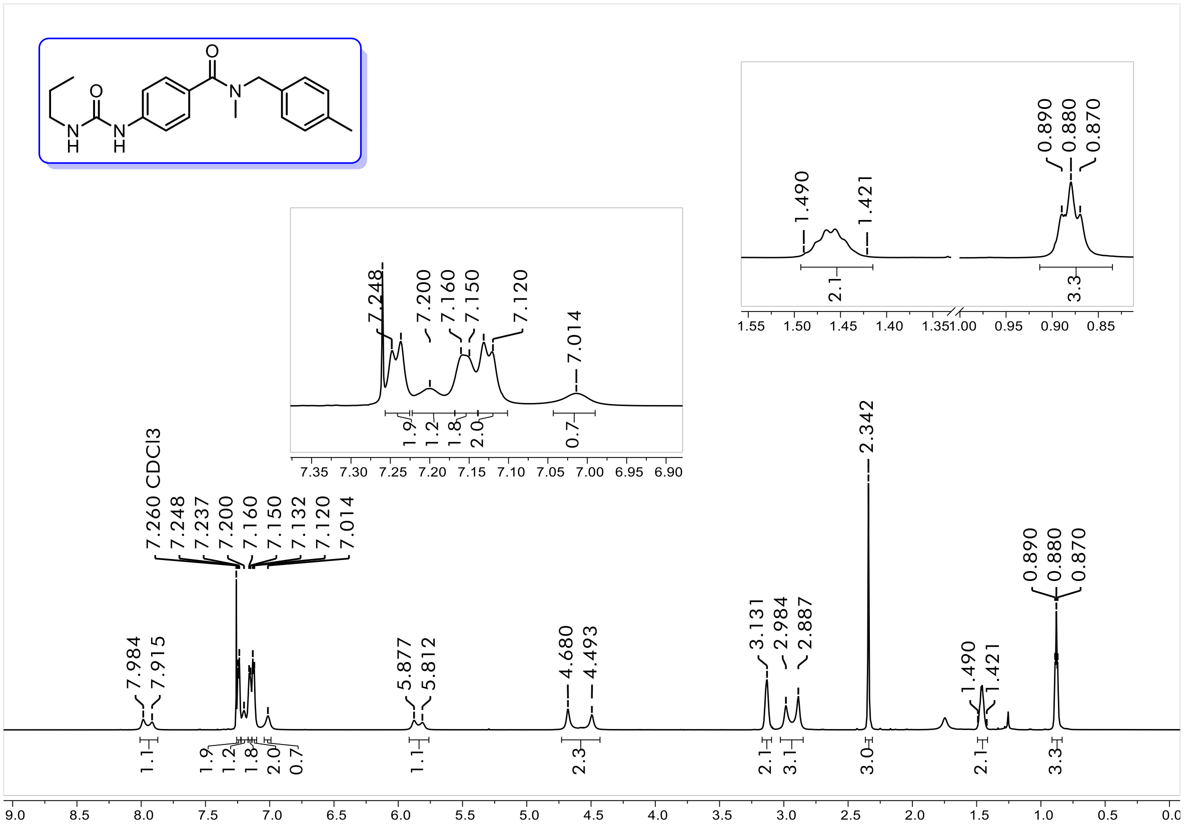

Spectrum 45. ^{1}H-NMR of *N*-methyl-*N*-(4-methylbenzyl)-4-(3-propylureido)benzamide (2b, mixture of rotamers).

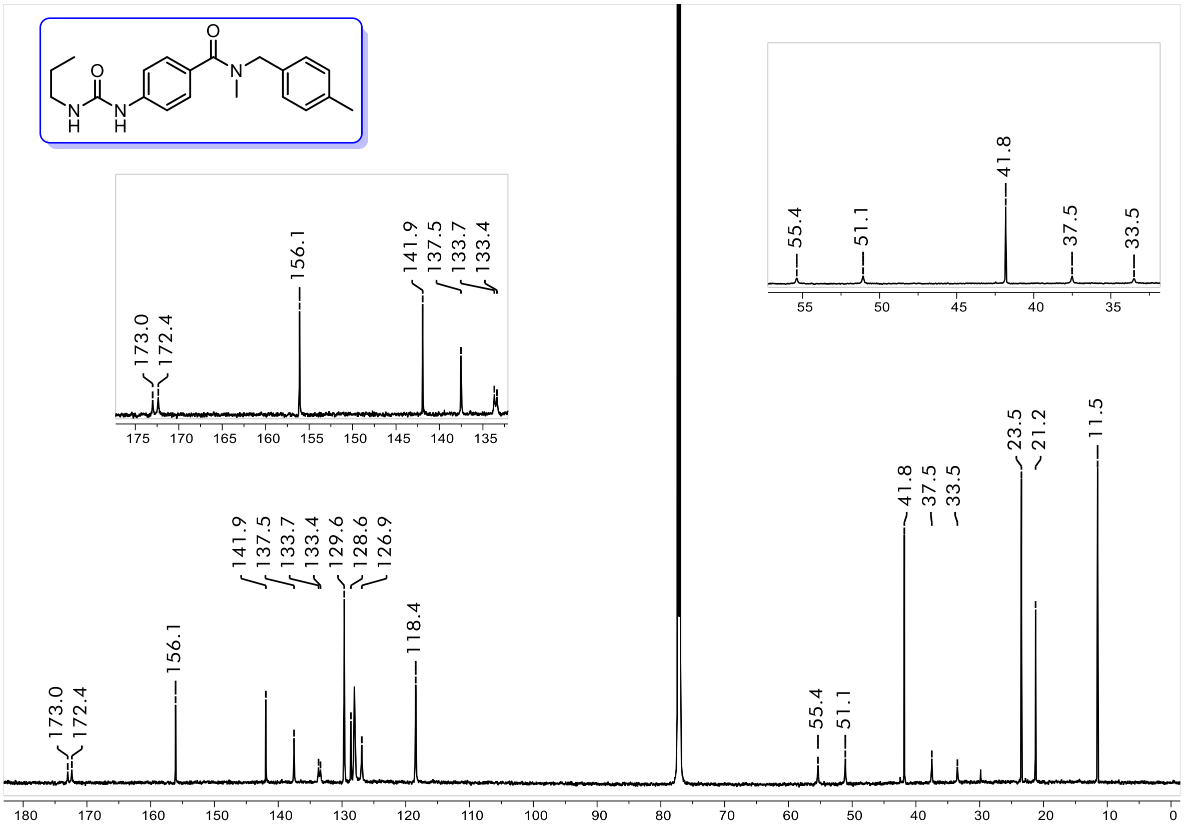

Spectrum 46. ^{13}C-NMR of *N*-methyl-*N*-(4-methylbenzyl)-4-(3-propylureido)benzamide (2b, mixture of rotamers).

Description:
Ionization Mode:ESI+
History:Determine m/z[Peak Detect[Centroid,30,Area];Correct Base[5.0%]];Correct Base[5.0%];Average(MS[1] 1..2)

Mass Calibration data:CAL_PEG_600_ok
Created:5/8/2024 10:58:12 AM
Created by:AccuTOF

Charge number:1 Tolerance:3.00(mmu)
Element:^{12}C:0 .. 28, ^{1}H:0 .. 26, ^{14}N:3 .. 3, ^{16}O:2 .. 2, ^{32}S:0 .. 0

Unsaturation Number:-1.5 .. 100.0 (Fraction:Both)

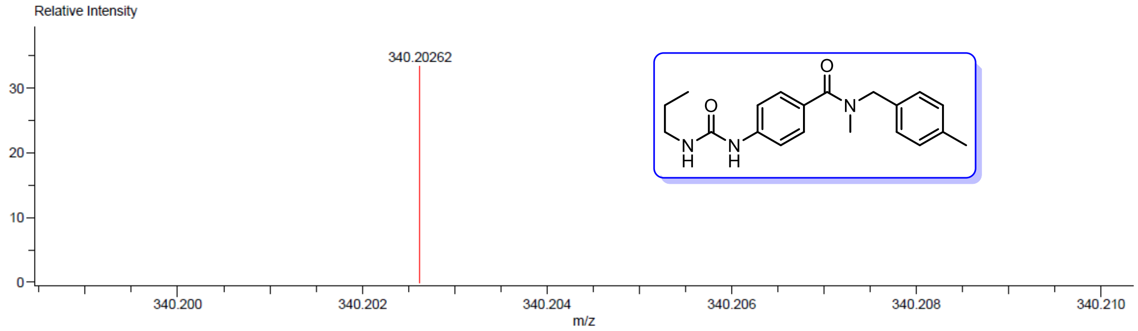

Mass	Intensity	Calc. Mass	Mass Difference (mmu)	Mass Difference (ppm)	Possible Formula	Unsaturation Number
340.20262	6513.68	340.20250	0.12	0.36	$^{12}C_{20}{}^{1}H_{26}{}^{14}N_3{}^{16}O_2$	9.5

Spectrum 47. HRMS of *N*-methyl-*N*-(4-methylbenzyl)-4-(3-propylureido)benzamide (2b, mixture of rotamers).

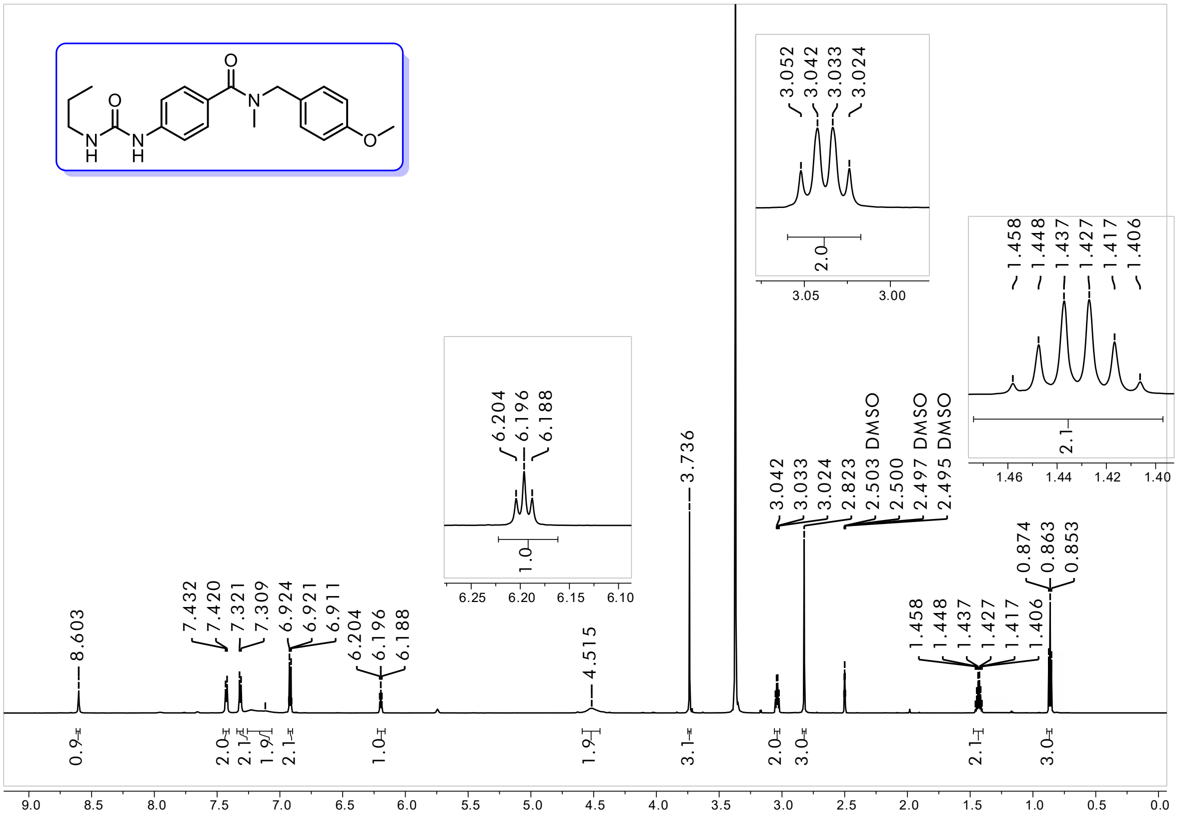

Spectrum 48. ^{1}H-NMR of *N*-(4-methoxybenzyl)-*N*-methyl-4-(3-propylureido)benzamide. (2c, mixture of rotamers).

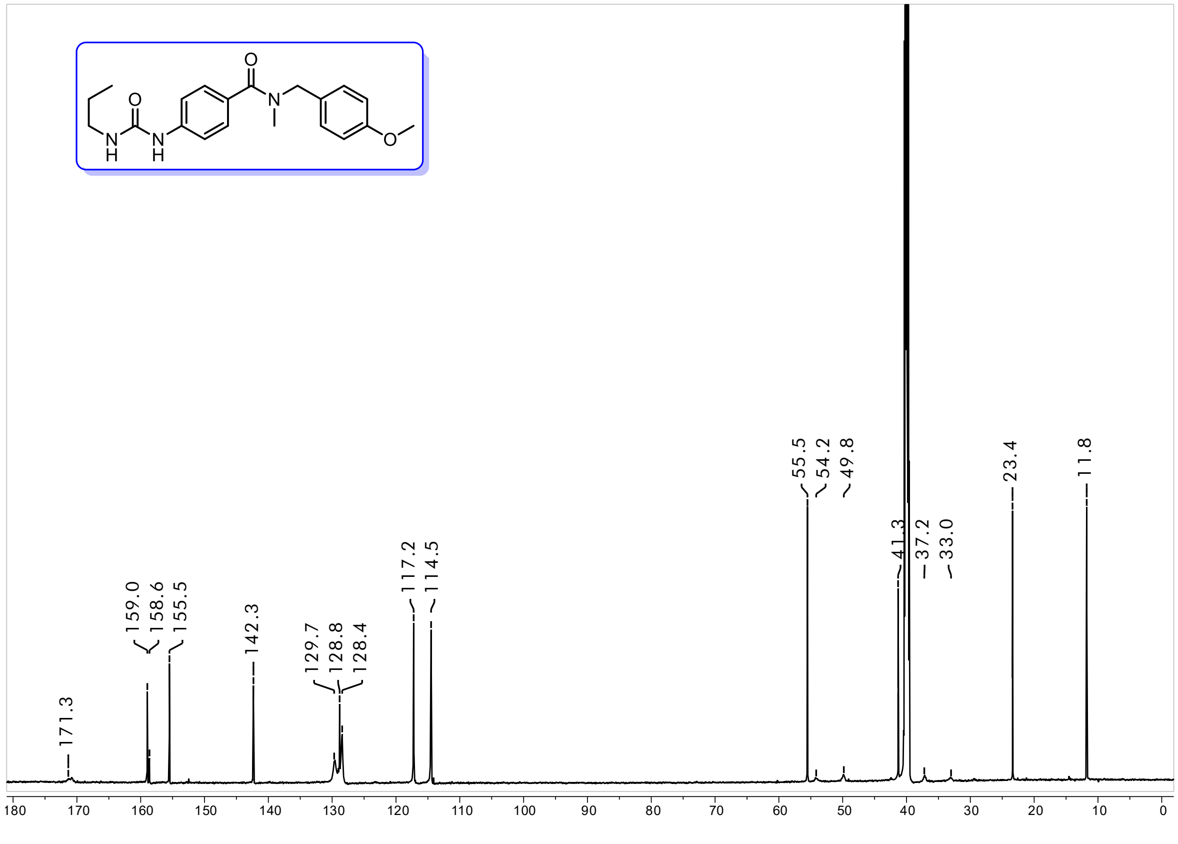

Spectrum 49. ^{13}C-NMR of *N*-(4-methoxybenzyl)-*N*-methyl-4-(3-propylureido)benzamide. (2c, mixture of rotamers).

Description:
Ionization Mode:ESI+
History:Determine m/z[Peak Detect[Centroid,30,Area];Correct Base[5.0%]];Correct Base[5.0%];Average(MS[1] 2..2)

Mass Calibration data:CAL_PEG_600
Created:6/18/2024 11:53:52 AM
Created by:AccuTOF

Charge number:1
Element:^{12}C:20 .. 40, ^{1}H:26 .. 26, ^{14}N:3 .. 3, ^{16}O:3 .. 3

Tolerance:100.00(mmu)

Unsaturation Number:-1.5 .. 1000.0 (Fraction:Both)

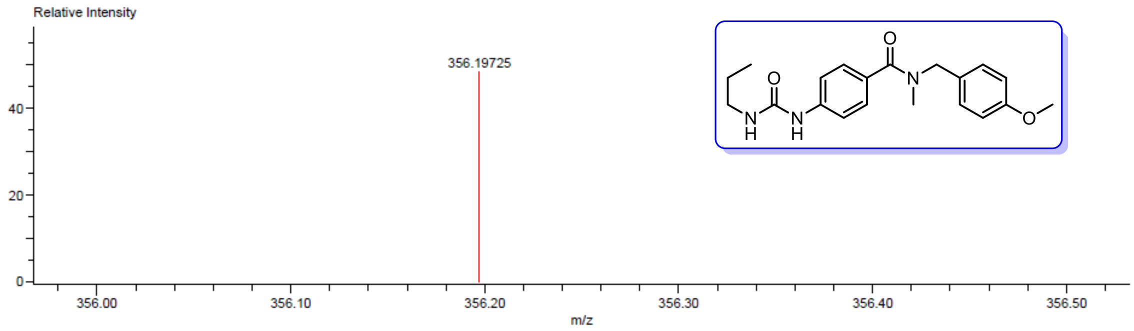

Mass	Intensity	Calc. Mass	Mass Difference (mmu)	Mass Difference (ppm)	Possible Formula	Unsaturation Number
356.19725	18156.07	356.19742	-0.17	-0.47	$^{12}C_{20}{}^{1}H_{26}{}^{14}N_{3}{}^{16}O_{3}$	9.5

Spectrum 50. HRMS of *N*-(4-methoxybenzyl)-*N*-methyl-4-(3-propylureido)benzamide. (2c, mixture of rotamers).

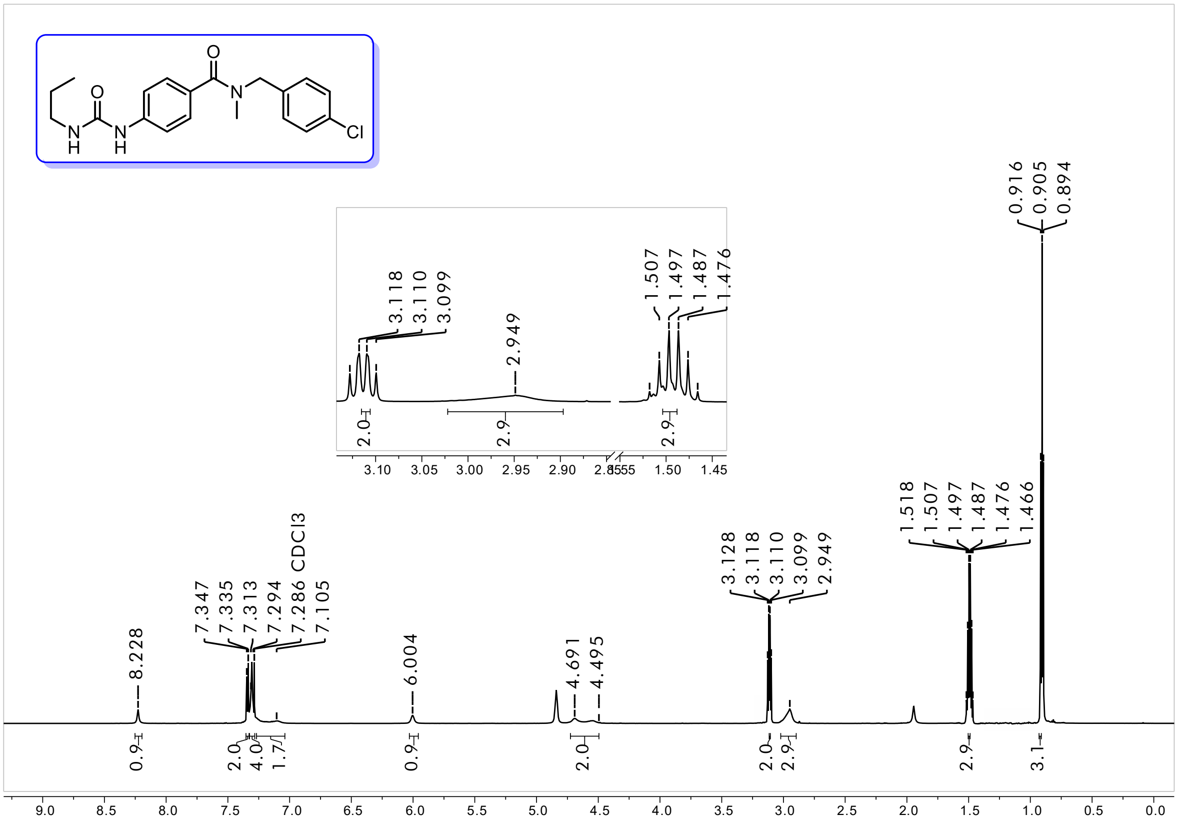

Spectrum 51. ^{1}**H-NMR of *N*-(4-chlorobenzyl)-*N*-methyl-4-(3-propylureido)benzamide (2d, mixture of rotamers).**

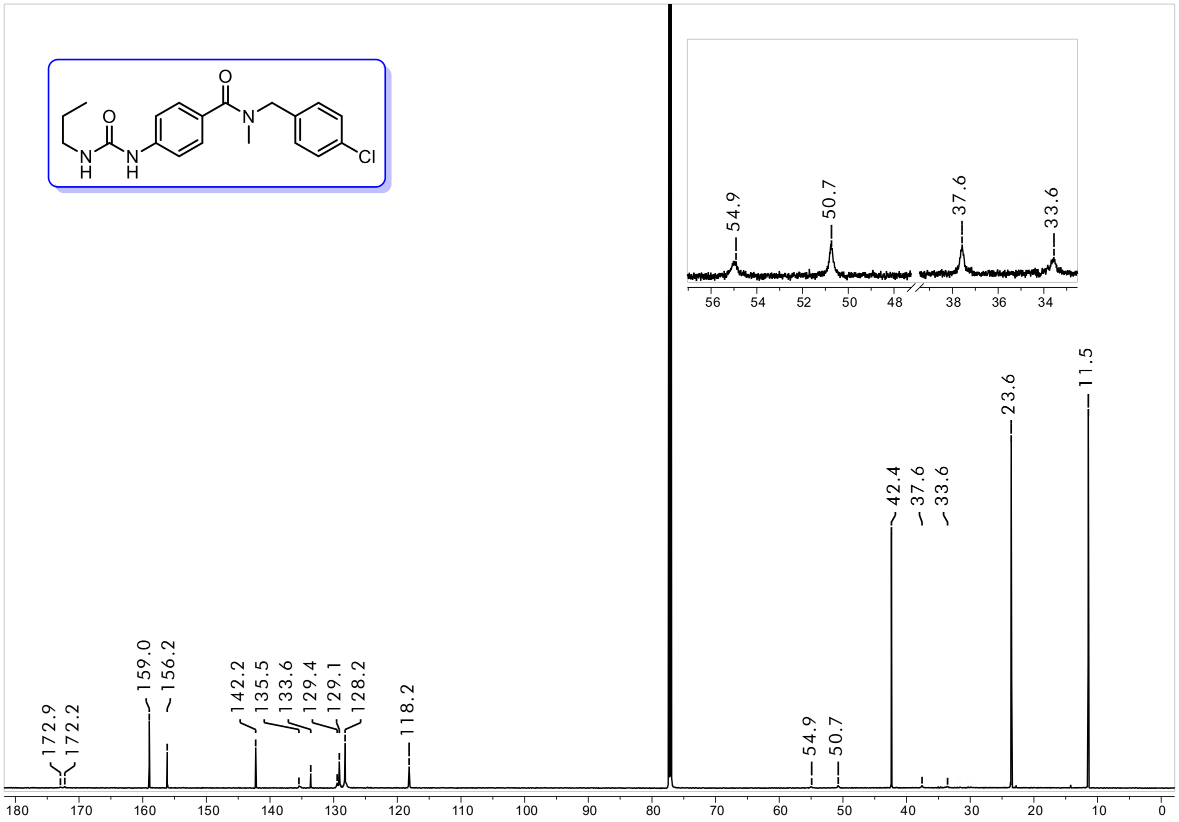

Spectrum 52. ^{13}**C-NMR of *N*-(4-chlorobenzyl)-*N*-methyl-4-(3-propylureido)benzamide (2d, mixture of rotamers).**

Description:
Ionization Mode:ESI+
History:Determine m/z[Peak Detect[Centroid,30,Area];Correct Base[5.0%]];Correct Base[5.0%];Average(MS[1] 2.0...

Mass Calibration data:CAL_PEG_600
Created:4/16/2024 10:03:20 AM
Created by:

Charge number:1
Tolerance:5.00(ppm), 5.00 .. 15.00(mmu)
Unsaturation Number:-1.5 .. 100.0 (Fraction:Both)
Element:^{12}C:0 .. 31, ^{1}H:0 .. 40, ^{35}Cl:0 .. 1, ^{14}N:3 .. 3, ^{16}O:2 .. 2

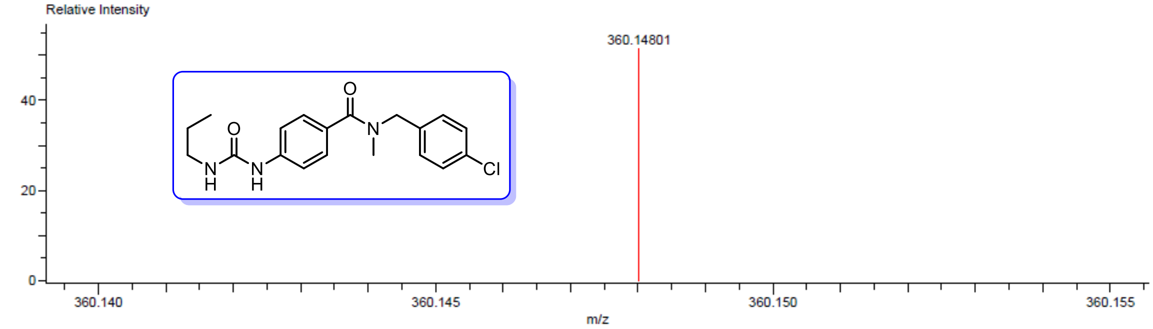

Mass	Intensity	Calc. Mass	Mass Difference (mmu)	Mass Difference (ppm)	Possible Formula	Unsaturation Number
360.14801	41910.00	360.14788	0.13	0.37	$^{12}C_{19}{}^{1}H_{23}{}^{35}Cl_{1}{}^{14}N_{3}{}^{16}O_{2}$	9.5

Spectrum 53. HRMS of *N*-(4-chlorobenzyl)-*N*-methyl-4-(3-propylureido)benzamide (2d, mixture of rotamers).

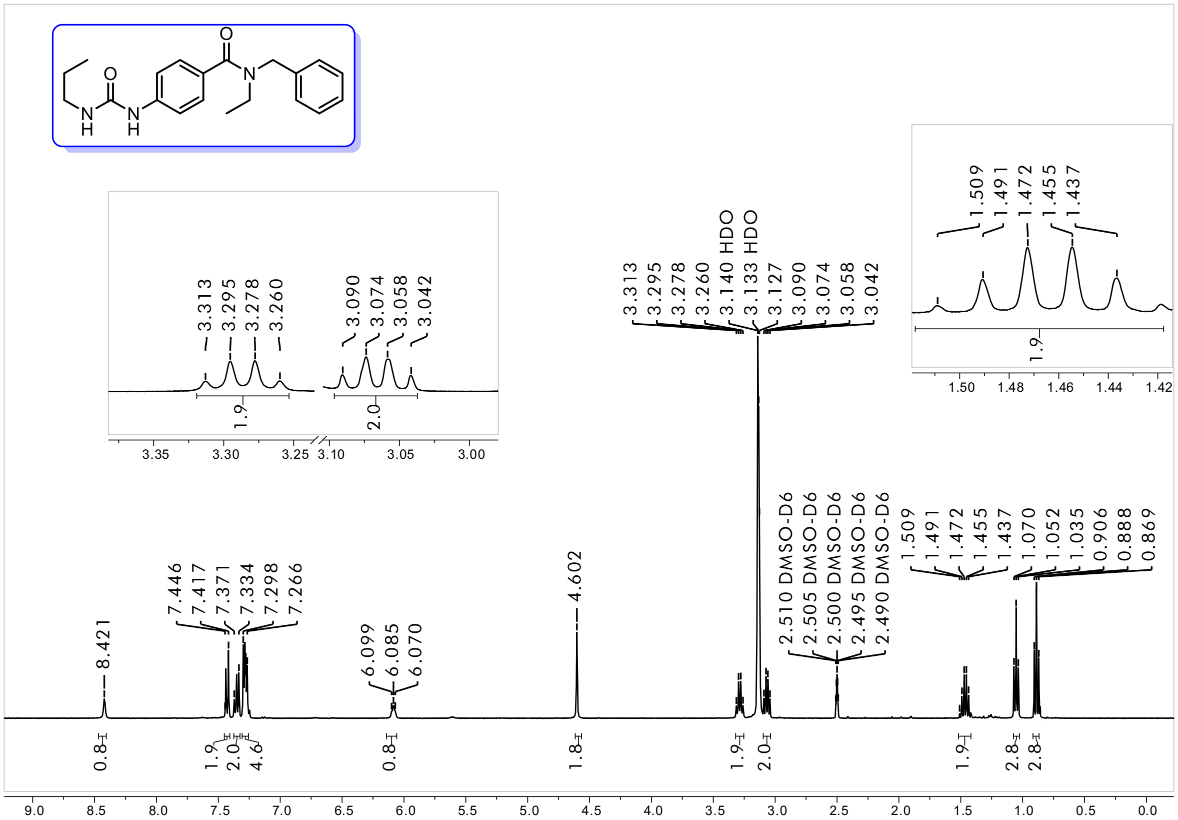

Spectrum 54. **^{1}H-NMR (80 °C) of *N*-benzyl-*N*-ethyl-4-(3-propylureido)benzamide (2e, mixture of rotamers).**

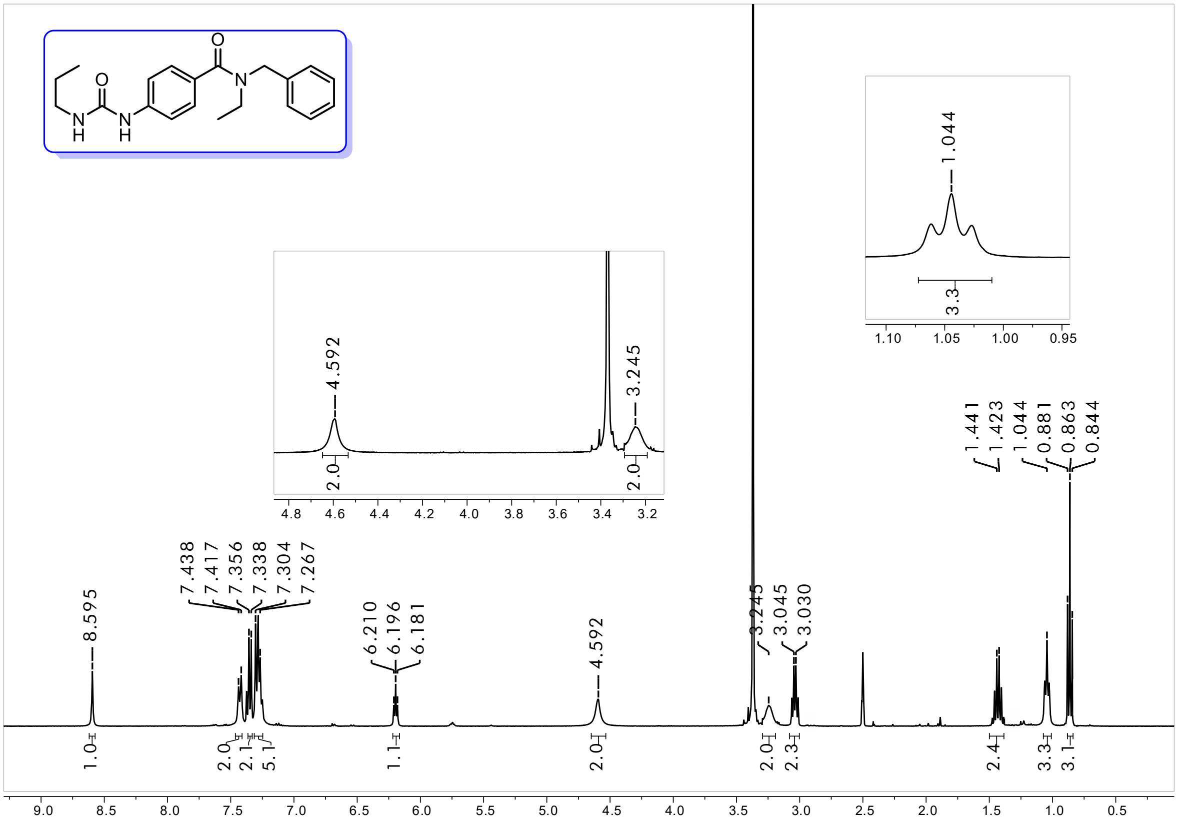

Spectrum 55. **^{1}H-NMR (25 °C) of *N*-benzyl-*N*-ethyl-4-(3-propylureido)benzamide (2e, mixture of rotamers).**

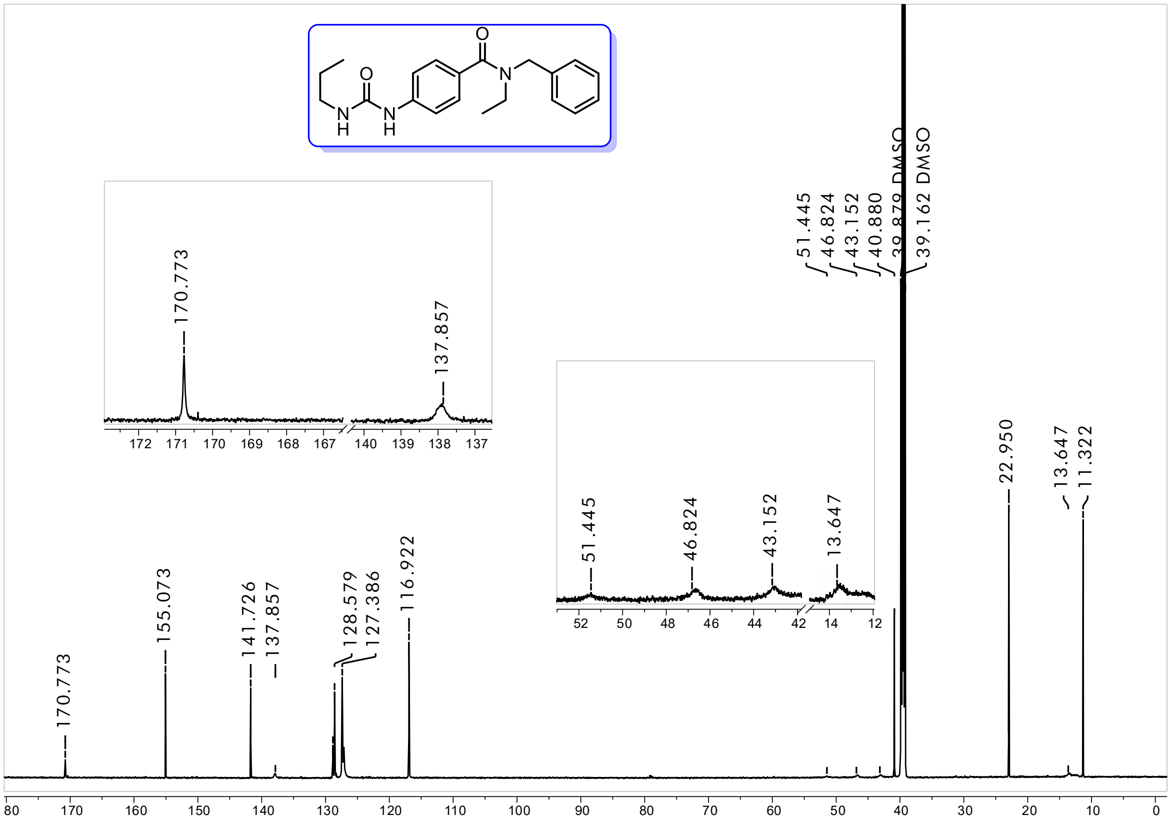

Spectrum 56. ¹³C-NMR (r.t.) of *N*-benzyl-*N*-ethyl-4-(3-propylureido)benzamide (2e, mixture of rotamers).

Description:
Ionization Mode:ESI+
History:Determine m/z[Peak Detect[Centroid,30,Area];Correct Base[5.0%]];Correct Base[5.0%];Average(MS[1] 0..0)

Mass Calibration data:CAL_PEG_600
Created:1/27/2025 1:06:49 PM
Created by:AccuTOF

Charge number:1
Tolerance:1000.00(ppm), 5.00 .. 15.00(mmu)
Unsaturation Number:-2.5 .. 200.0 (Fraction:Both)
Element:^{12}C:0 .. 20, ^{1}H:0 .. 30, ^{35}Cl:0 .. 0, ^{14}N:3 .. 3, ^{16}O:2 .. 2

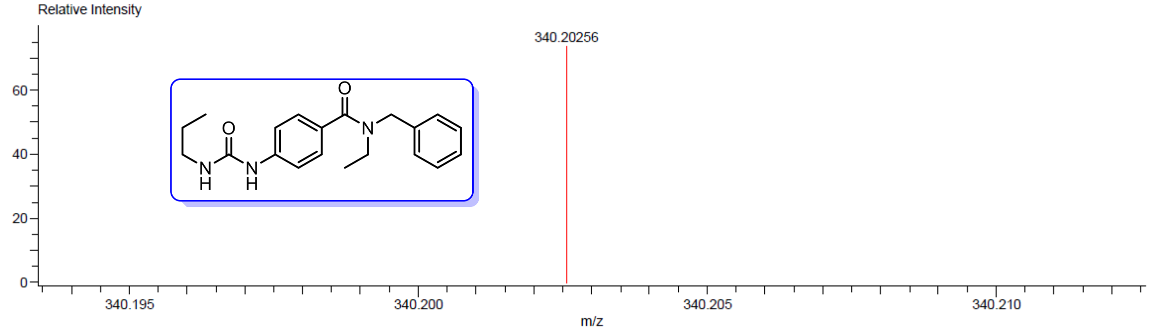

Mass	Intensity	Calc. Mass	Mass Difference (mmu)	Mass Difference (ppm)	Possible Formula	Unsaturation Number
340.20256	20030.27	340.20250	0.06	0.18	$^{12}C_{20}{}^{1}H_{26}{}^{14}N_{3}{}^{16}O_{2}$	9.5

Spectrum 57. HRMS of *N*-benzyl-*N*-ethyl-4-(3-propylureido)benzamide (2e, mixture of rotamers).

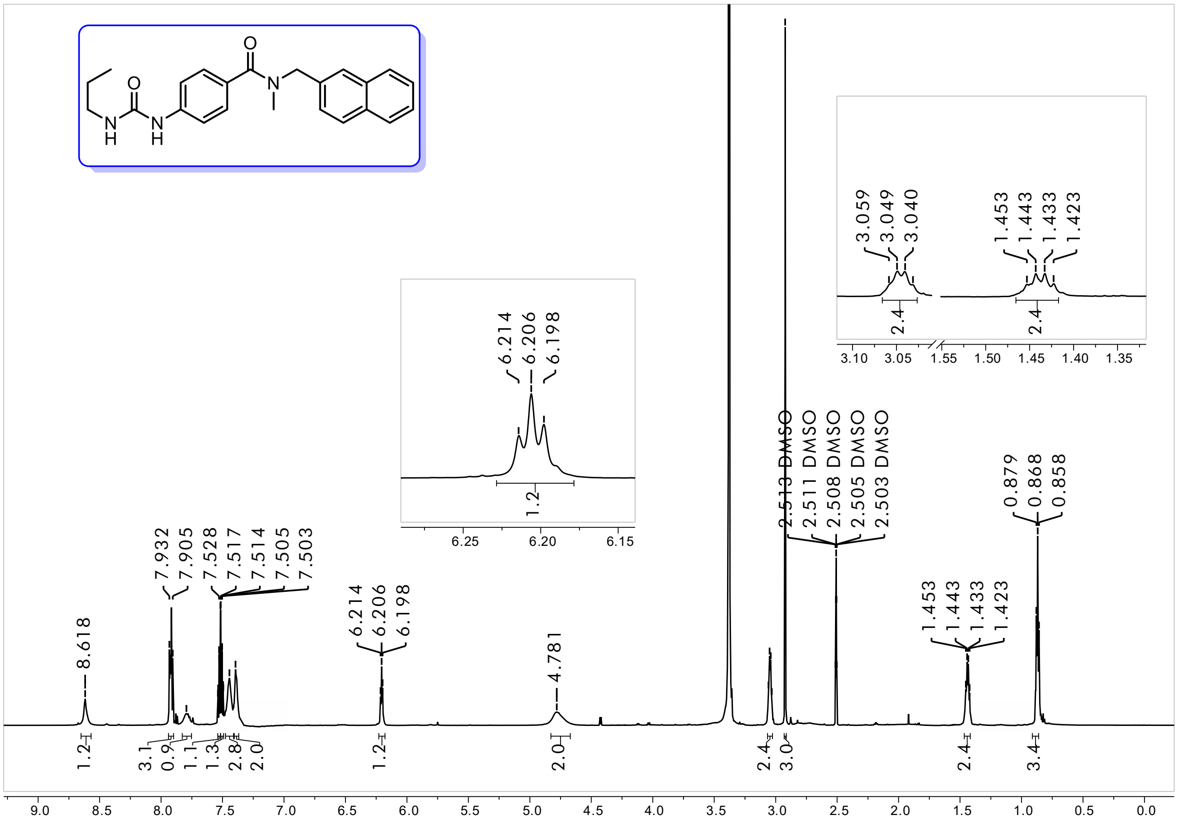

Spectrum 58. ^{1}**H-NMR of** ***N*****-methyl-*****N*****-(naphthalen-2-ylmethyl)-4-(3-propylureido)benzamide (2f, mixture of rotamers).**

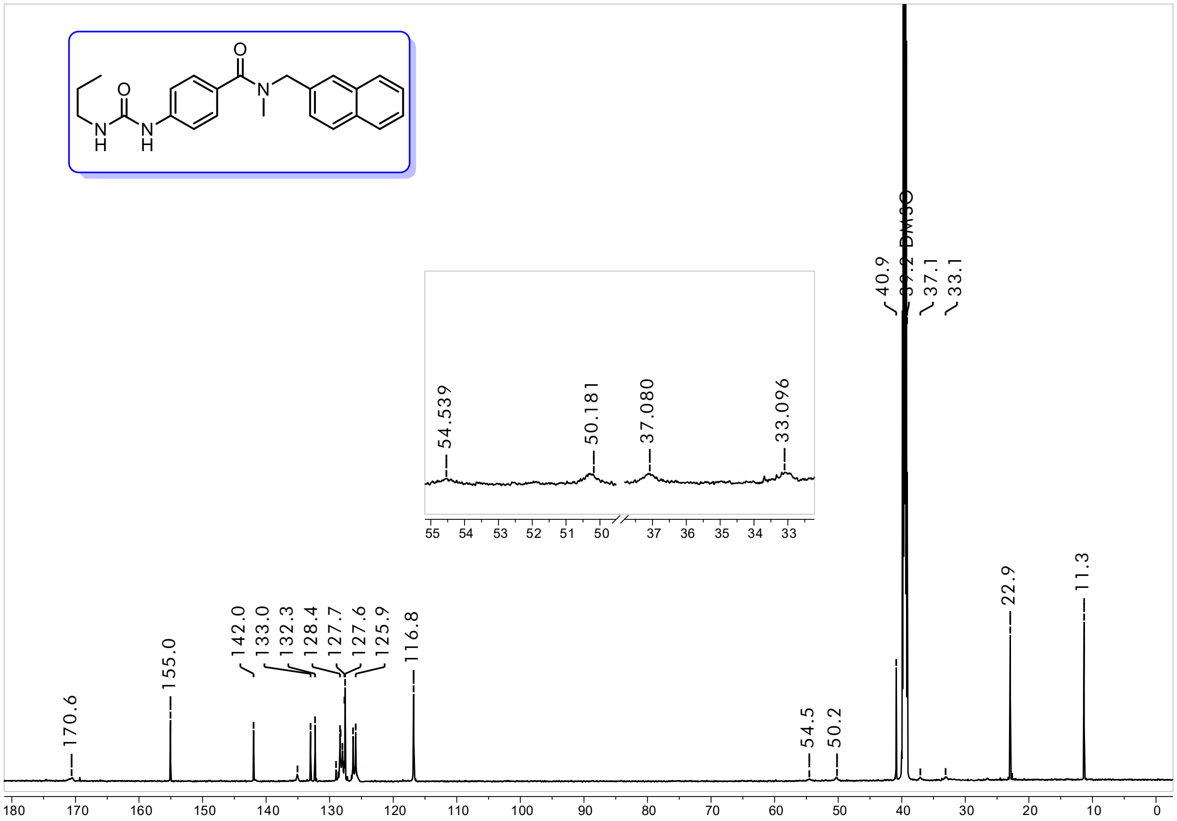

Spectrum 59. ^{13}**C-NMR of** ***N*****-methyl-*****N*****-(naphthalen-2-ylmethyl)-4-(3-propylureido)benzamide (2f, mixture of rotamers).**

Description:
Ionization Mode:ESI+
History:Determine m/z[Peak Detect[Centroid,30,Area];Correct Base[5.0%]];Correct Base[5.0%];Average(MS[1] 1..1)

Mass Calibration data:CAL_PEG_600
Created:3/6/2025 12:23:37 PM
Created by:AccuTOF

Charge number:1
Element:^{12}C:0 .. 23, ^{1}H:0 .. 26, ^{14}N:3 .. 3, ^{16}O:2 .. 2

Tolerance:20.00(mmu)

Unsaturation Number:-2.0 .. 200.0 (Fraction:Both)

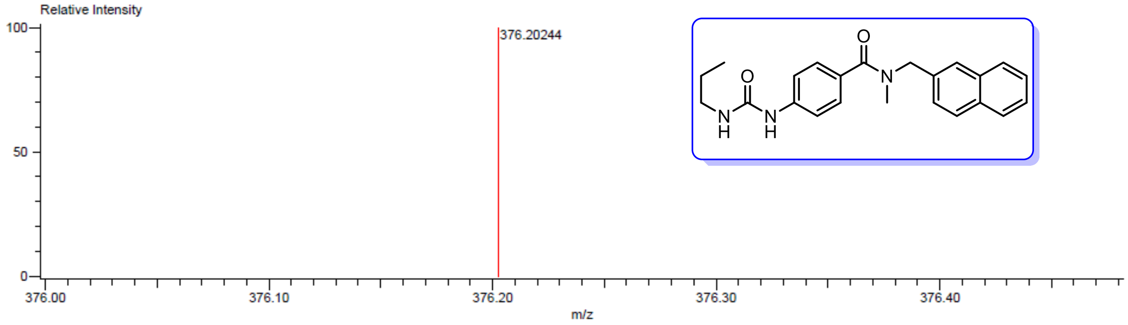

Mass	Intensity	Calc. Mass	Mass Difference (mmu)	Mass Difference (ppm)	Possible Formula	Unsaturation Number
376.20244	5452.00	376.20250	-0.06	-0.15	$^{12}C_{23}{}^{1}H_{26}{}^{14}N_{3}{}^{16}O_{2}$	12.5

Spectrum 60. HRMS of *N*-methyl-*N*-(naphthalen-2-ylmethyl)-4-(3-propylureido)benzamide (2f, mixture of rotamers).

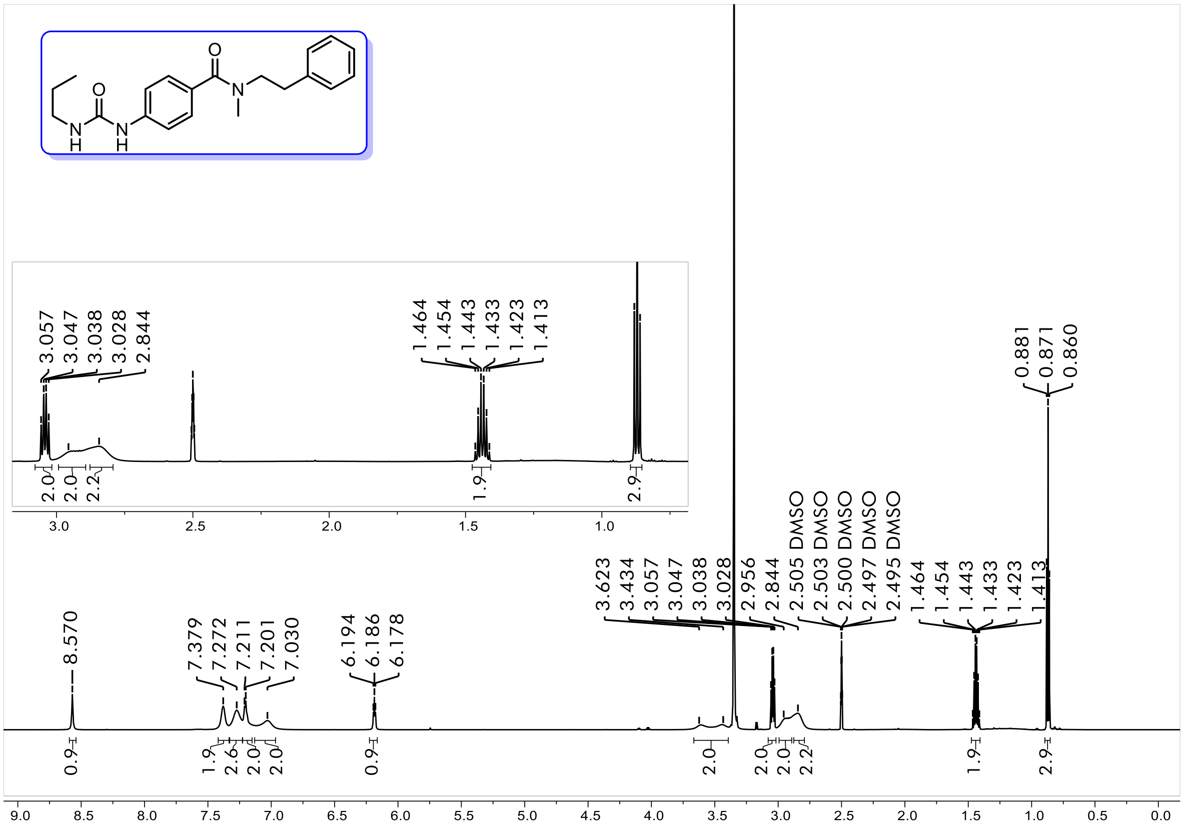

Spectrum 61. **^{1}H-NMR of *N*-methyl-*N*-phenethyl-4-(3-propylureido)benzamide(2g, mixture of rotamers).**

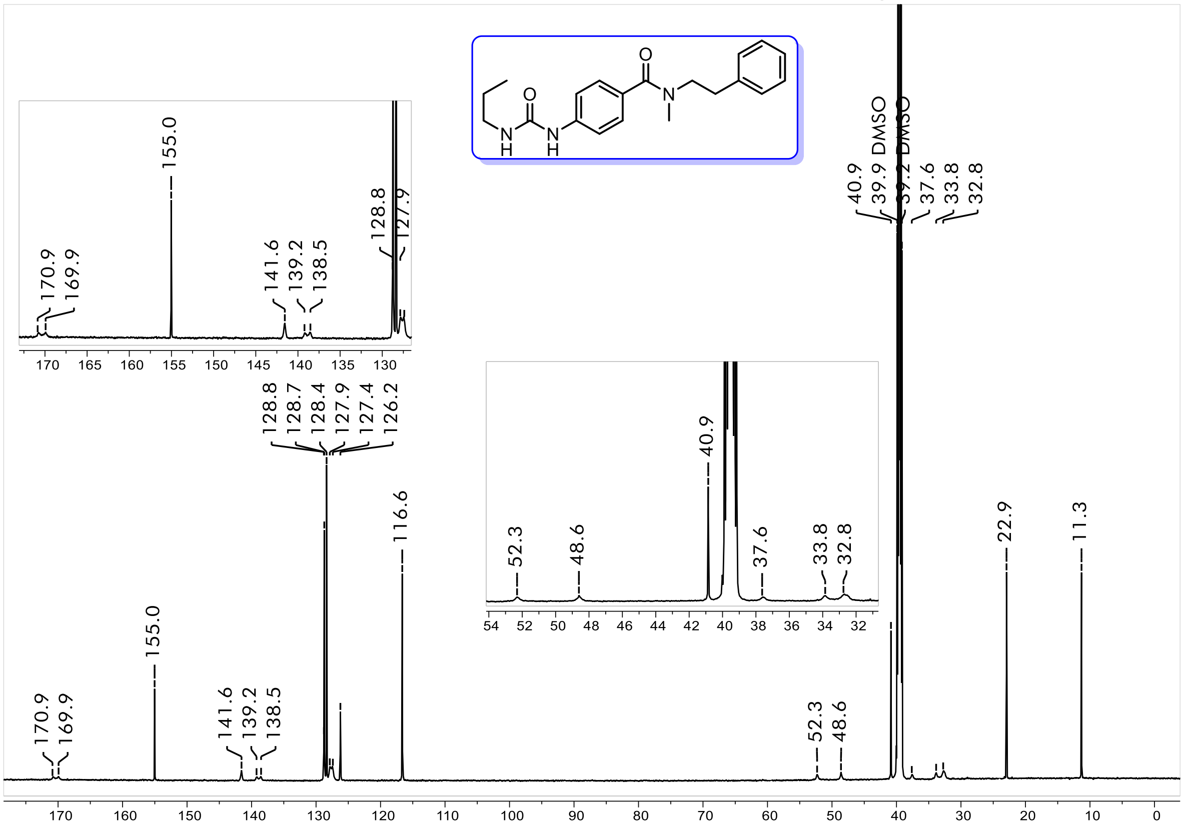

Spectrum 62. **^{13}C-NMR of *N*-methyl-*N*-phenethyl-4-(3-propylureido)benzamide(2g, mixture of rotamers).**

Description:
Ionization Mode:ESI+
History:Determine m/z[Peak Detect[Centroid,30,Area];Correct Base[5.0%]];Correct Base[5.0%];Average(MS[1] 1..1)

Mass Calibration data:CAL_PEG_600
Created:2/7/2025 1:11:47 PM
Created by:AccuTOF

Charge number:1
Element:^{12}C:0 .. 20, ^{1}H:0 .. 30, ^{14}N:3 .. 3, ^{16}O:0 .. 2

Tolerance:2.00(ppm), 5.00 .. 15.00(mmu)

Unsaturation Number:-2.0 .. 200.0 (Fraction:Both)

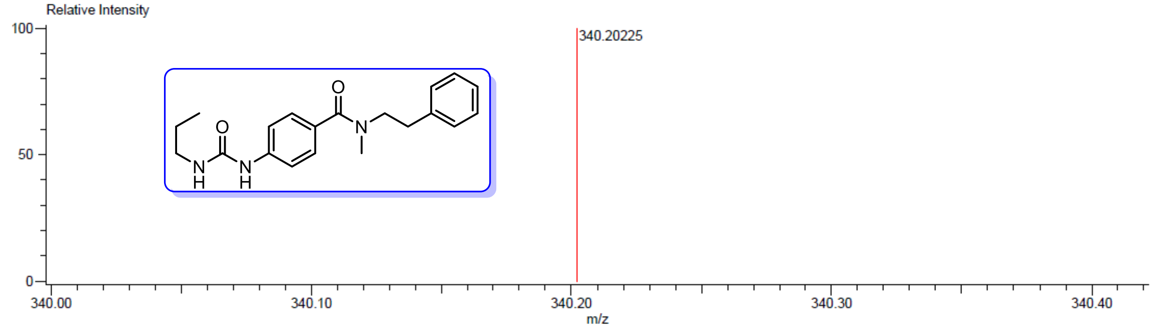

Mass	Intensity	Calc. Mass	Mass Difference (mmu)	Mass Difference (ppm)	Possible Formula	Unsaturation Number
340.20225	26321.50	340.20250	-0.26	-0.75	$^{12}C_{20}{}^{1}H_{26}{}^{14}N_{3}{}^{16}O_{2}$	9.5

Spectrum 63. HRMS of *N*-methyl-*N*-phenethyl-4-(3-propylureido)benzamide(2g, mixture of rotamers).

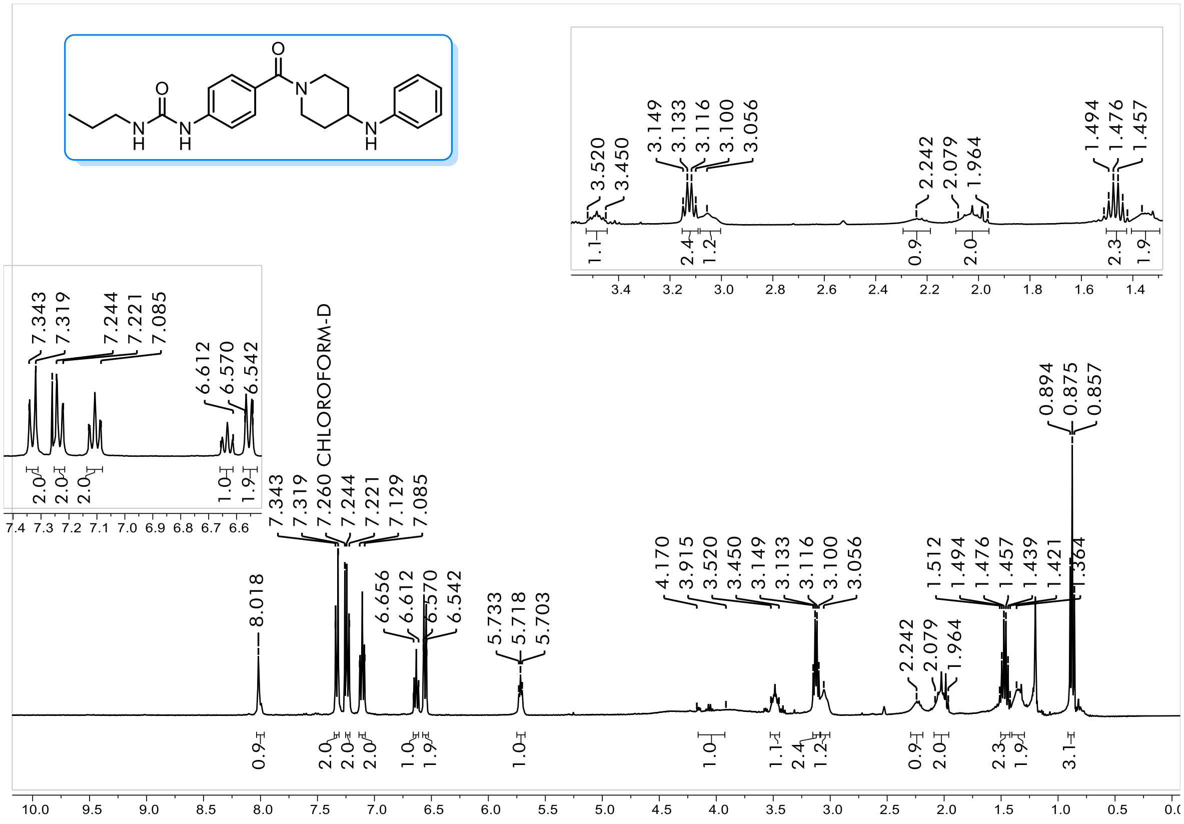

Spectrum 64. ^{1}H-NMR of 1-(4-(4-(phenylamino)piperidine-1-carbonyl)phenyl)-3-propylurea (3a).

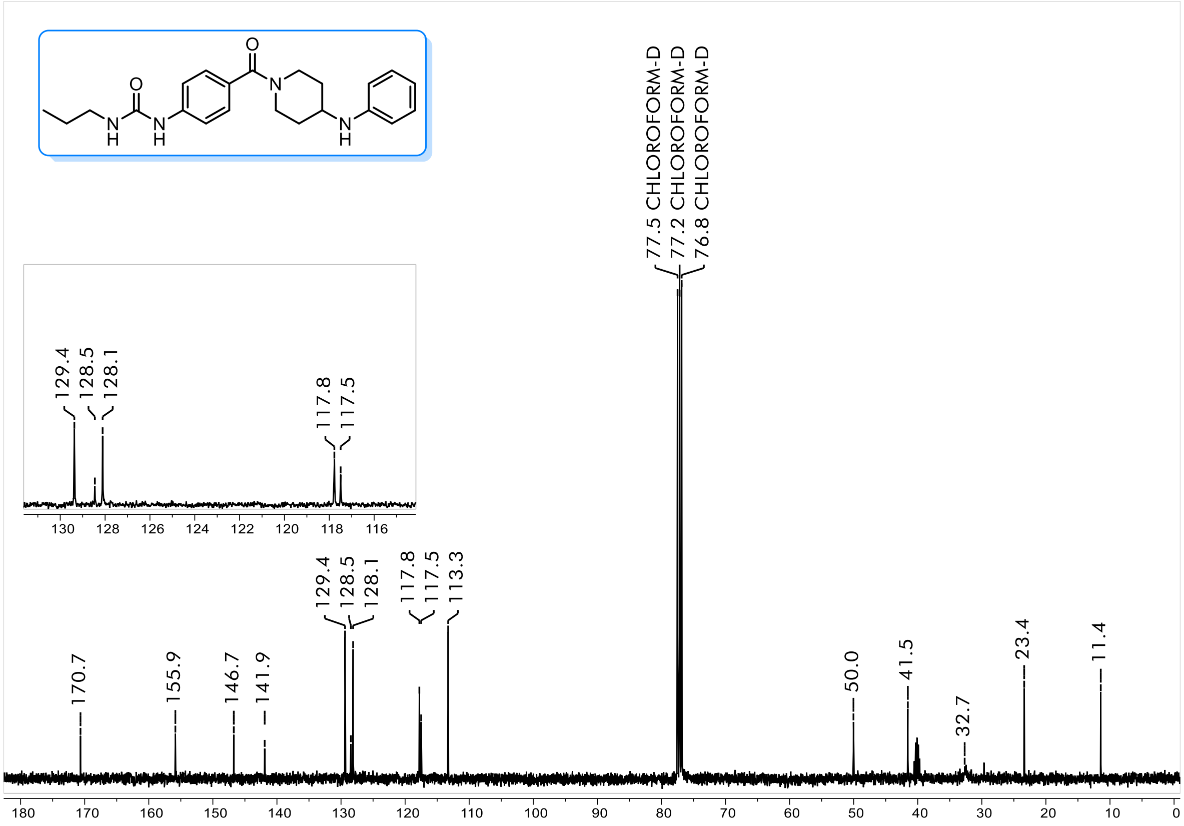

Spectrum 65. ^{13}C-NMR of 1-(4-(4-(phenylamino)piperidine-1-carbonyl)phenyl)-3-propylurea (3a).

Description:
Ionization Mode:ESI+
History:Determine m/z[Peak Detect[Centroid,30,Area];Correct Base[];Smooth[5]];Correct Base[5.0%];Average(MS[...

Mass Calibration data:Cal_PEG_600
Created:2/6/2024 2:53:04 PM
Created by:AccuTOF

Charge number:1
Tolerance:3.00(ppm), 5.00 .. 15.00(mmu)
Unsaturation Number:-1.0 .. 30.0 (Fraction:Both)
Element:^{12}C:0 .. 50, ^{1}H:0 .. 40, ^{79}Br:0 .. 1, ^{14}N:4 .. 4, ^{16}O:2 .. 2

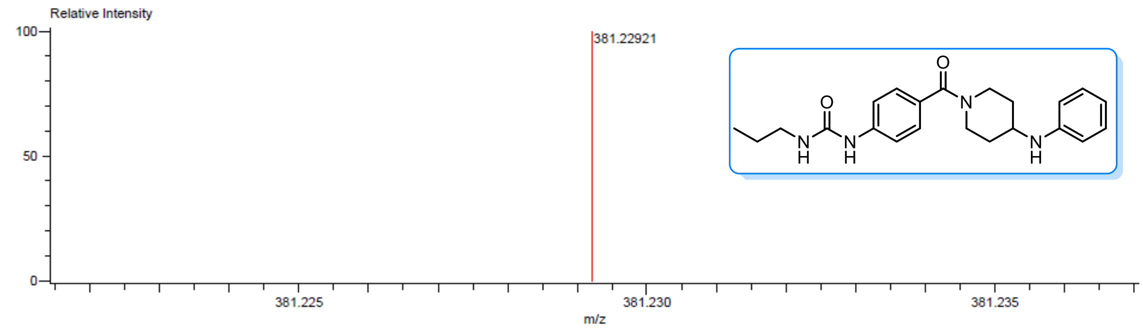

Mass	Intensity	Calc. Mass	Mass Difference (mmu)	Mass Difference (ppm)	Possible Formula	Unsaturation Number
381.22921	41060.16	381.22905	0.16	0.43	$^{12}C_{22}{}^{1}H_{29}{}^{14}N_4{}^{16}O_2$	10.5

Spectrum 66. HRMS of 1-(4-(4-(phenylamino)piperidine-1-carbonyl)phenyl)-3-propylurea (3a).

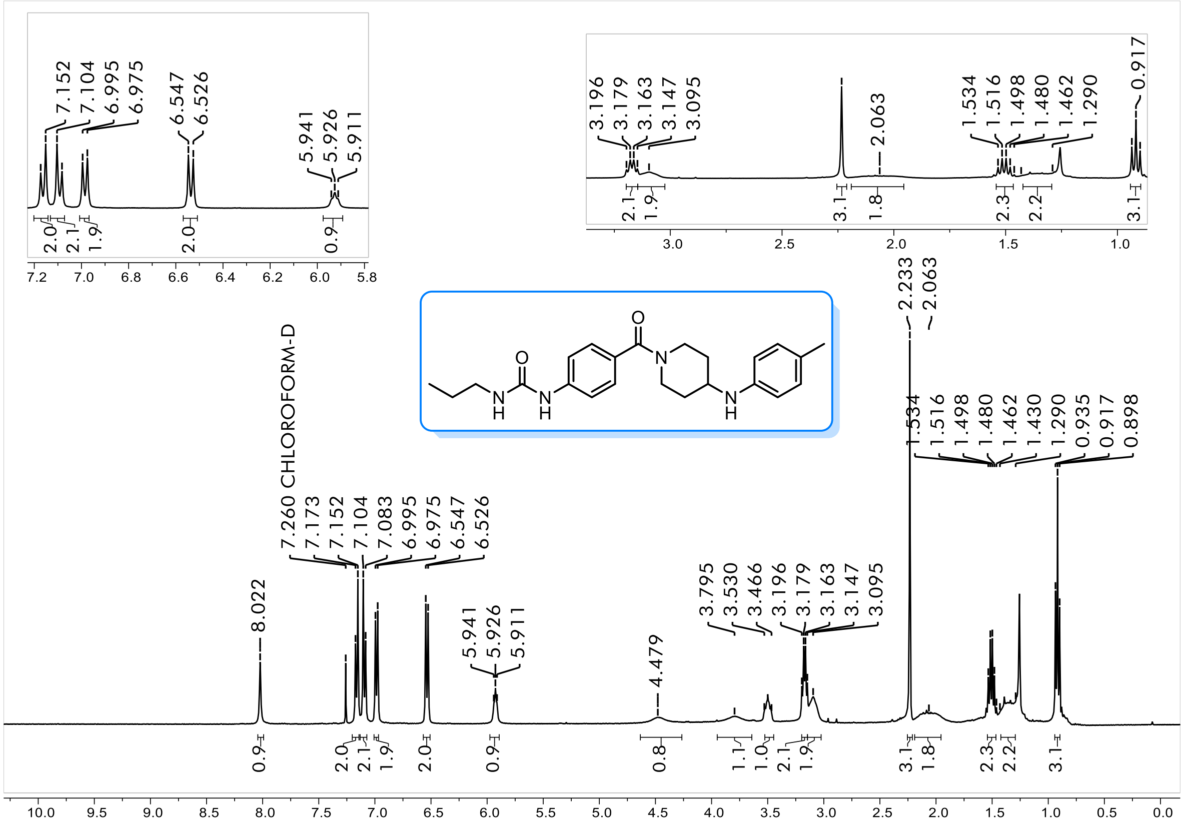

Spectrum 67. ^{1}H-NMR of 1-propyl-3-(4-(4-(*p*-tolylamino)piperidine-1-carbonyl)phenyl)urea (3b).

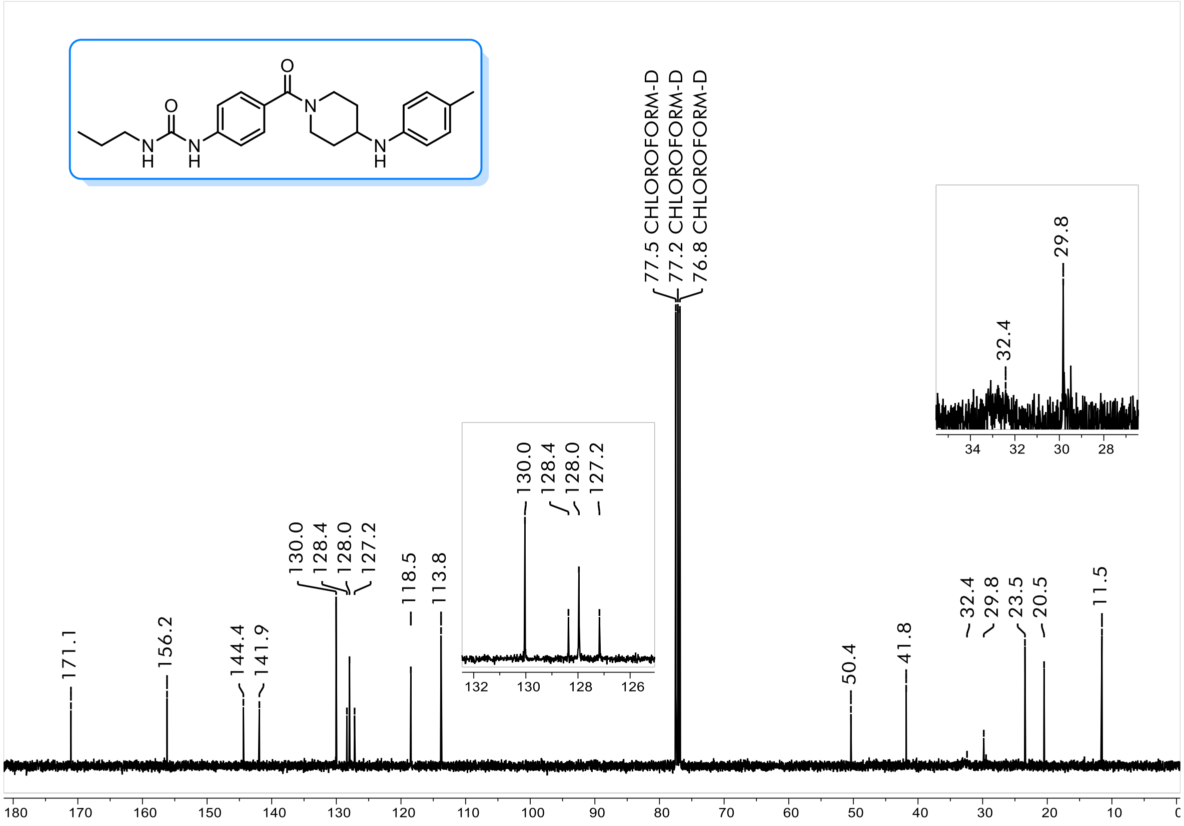

Spectrum 68. ^{13}C-NMR of 1-propyl-3-(4-(4-(*p*-tolylamino)piperidine-1-carbonyl)phenyl)urea (3b).

[illegible]
Description:
Ionization Mode:ESI+
History:Determine m/z[Peak Detect[Centroid,30,Area];Correct Base[];Smooth[5]];Correct Base[5.0%];Average(MS[...

[illegible]
Mass Calibration data:Cal_PEG_600
Created:2/28/2024 10:50:37 AM
Created by:AccuTOF

Charge number:1
Element:^{12}C:0 .. 40, ^{1}H:0 .. 50, ^{14}N:4 .. 4, ^{16}O:2 .. 2

Tolerance:3.00(mmu)

Unsaturation Number:-1.0 .. 20.0 (Fraction:Both)

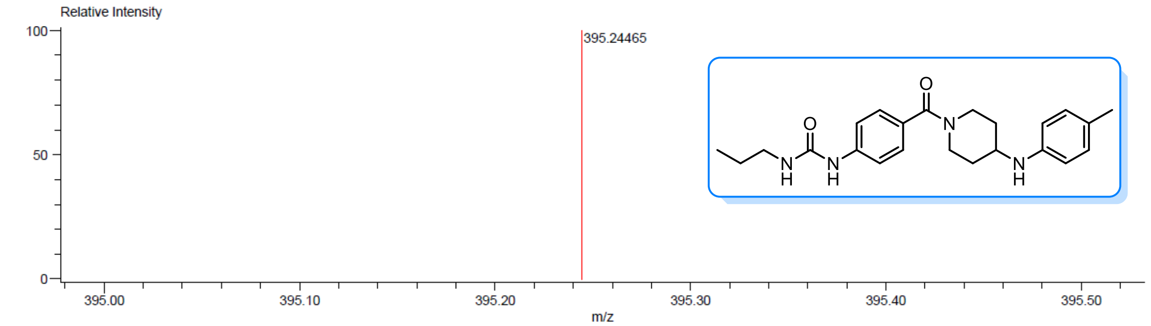

Mass	Intensity	Calc. Mass	Mass Difference (mmu)	Mass Difference (ppm)	Possible Formula	Unsaturation Number
395.24465	116707.57	395.24470	-0.05	-0.13	$^{12}C_{23}{}^{1}H_{31}{}^{14}N_4{}^{16}O_2$	10.5

Spectrum 69. HRMS of 1-propyl-3-(4-(4-(*p*-tolylamino)piperidine-1-carbonyl)phenyl)urea (3b).

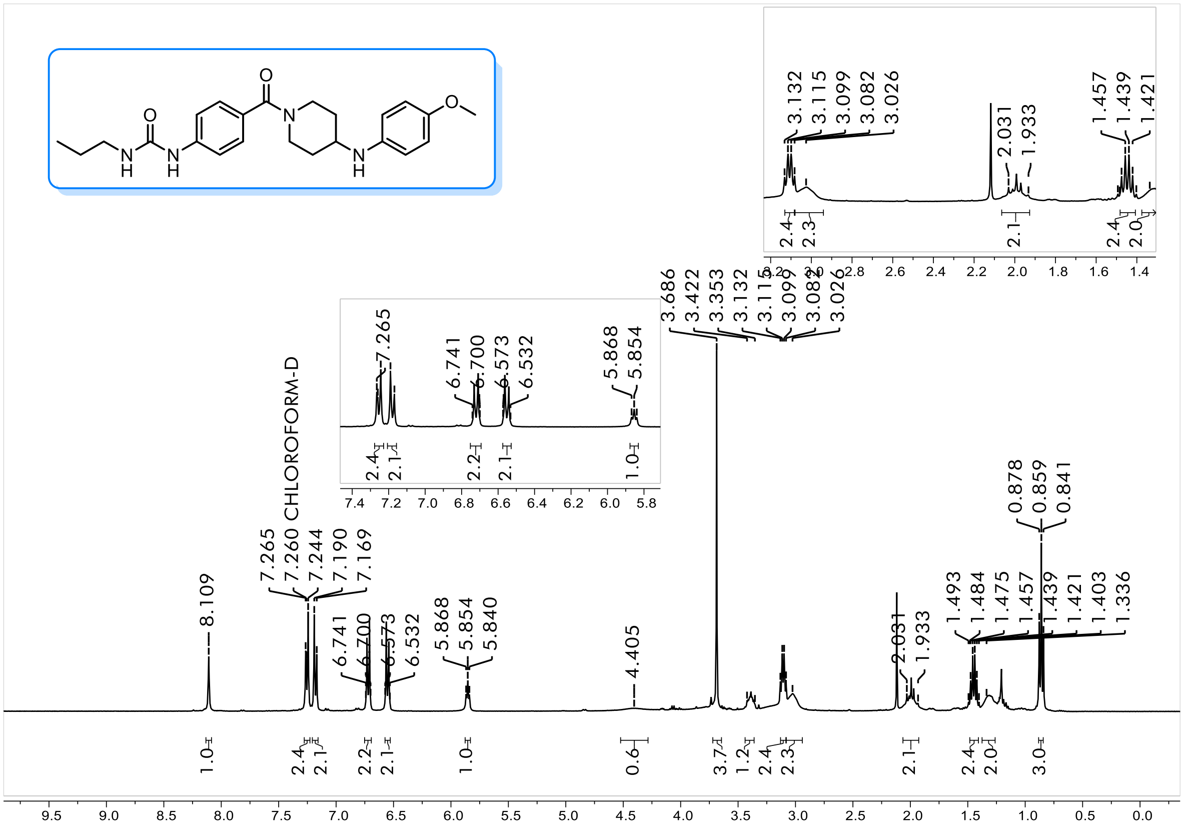

Spectrum 70. ^{1}**H-NMR of 1-(4-(4-((4-methoxyphenyl)amino)piperidine-1-carbonyl)phenyl)-3-propylurea (3c).**

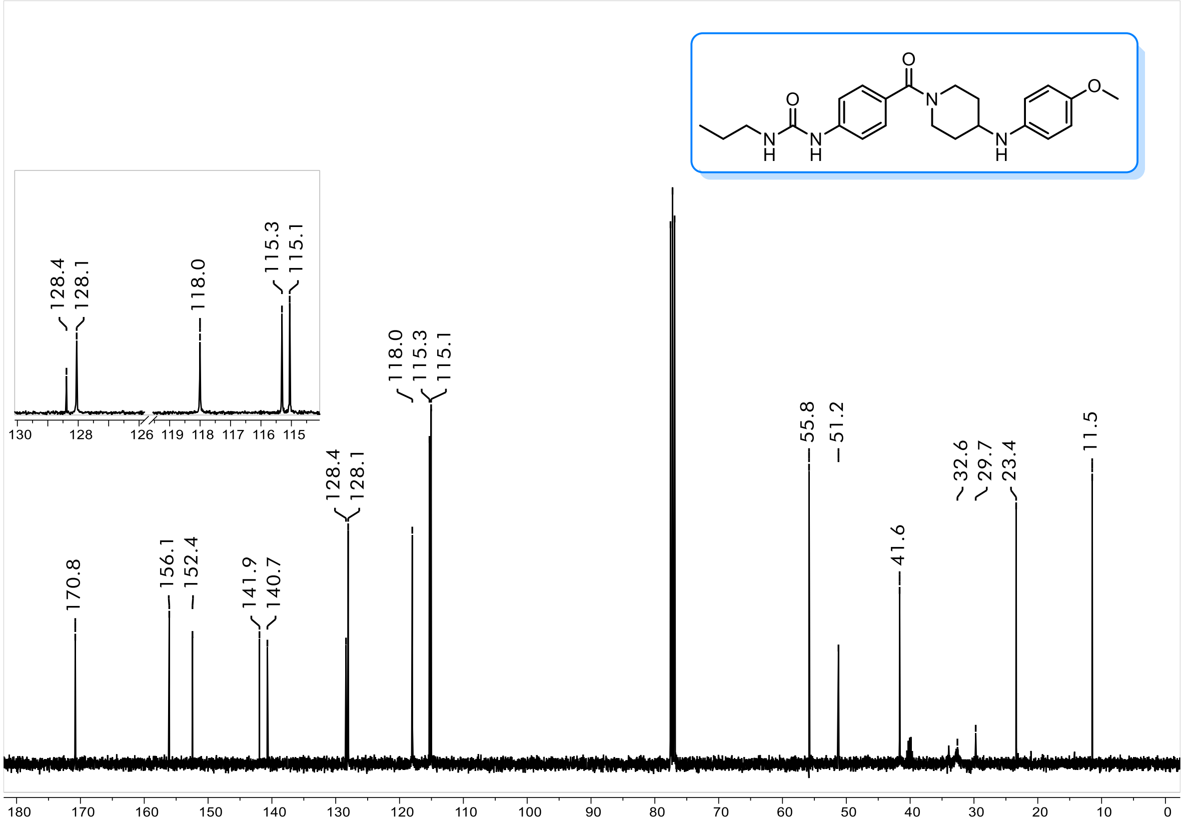

Spectrum 71. ^{13}**C-NMR of 1-(4-(4-((4-methoxyphenyl)amino)piperidine-1-carbonyl)phenyl)-3-propylurea (3c).**

Description:
Ionization Mode:ESI+
History:Determine m/z[Peak Detect[Centroid,30,Area];Correct Base[5.0%]];Correct Base[5.0%];Average(MS[1] 2.2...

Mass Calibration data:CAL_PEG_600
Created:4/9/2024 12:37:59 PM
Created by:AccuTOF

Charge number:1
Element:^{12}C:0 .. 40, ^{1}H:0 .. 70, ^{14}N:4 .. 4, ^{16}O:3 .. 3

Tolerance:100.00(ppm), 5.00 .. 15.00(mmu)

Unsaturation Number:-1.5 .. 100.0 (Fraction:Both)

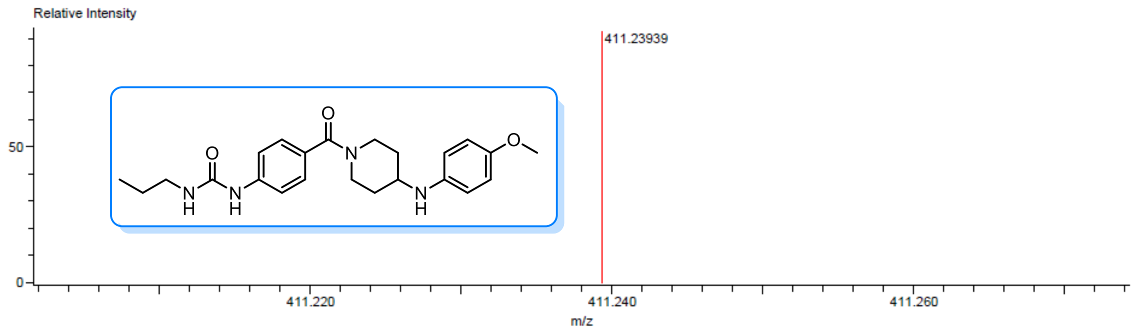

Mass	Intensity	Calc. Mass	Mass Difference (mmu)	Mass Difference (ppm)	Possible Formula	Unsaturation Number
411.23939	97281.25	411.23961	-0.22	-0.54	$^{12}C_{23}{}^{1}H_{31}{}^{14}N_4{}^{16}O_3$	10.5

Spectrum 72. HRMS of 1-(4-(4-((4-methoxyphenyl)amino)piperidine-1-carbonyl)phenyl)-3-propylurea (3c).

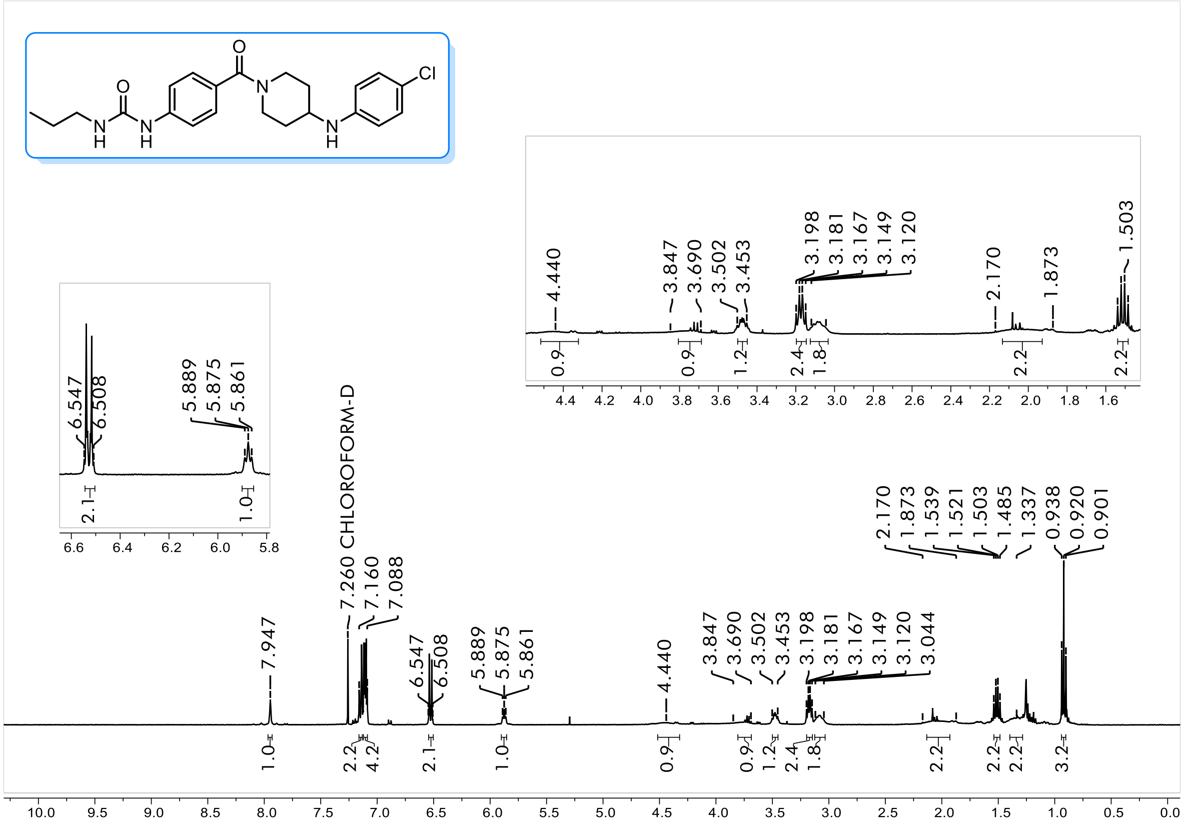

Spectrum 73. ^{1}H-NMR of 1-(4-(4-((4-chlorophenyl)amino)piperidine-1-carbonyl)phenyl)-3-propylurea (3b).

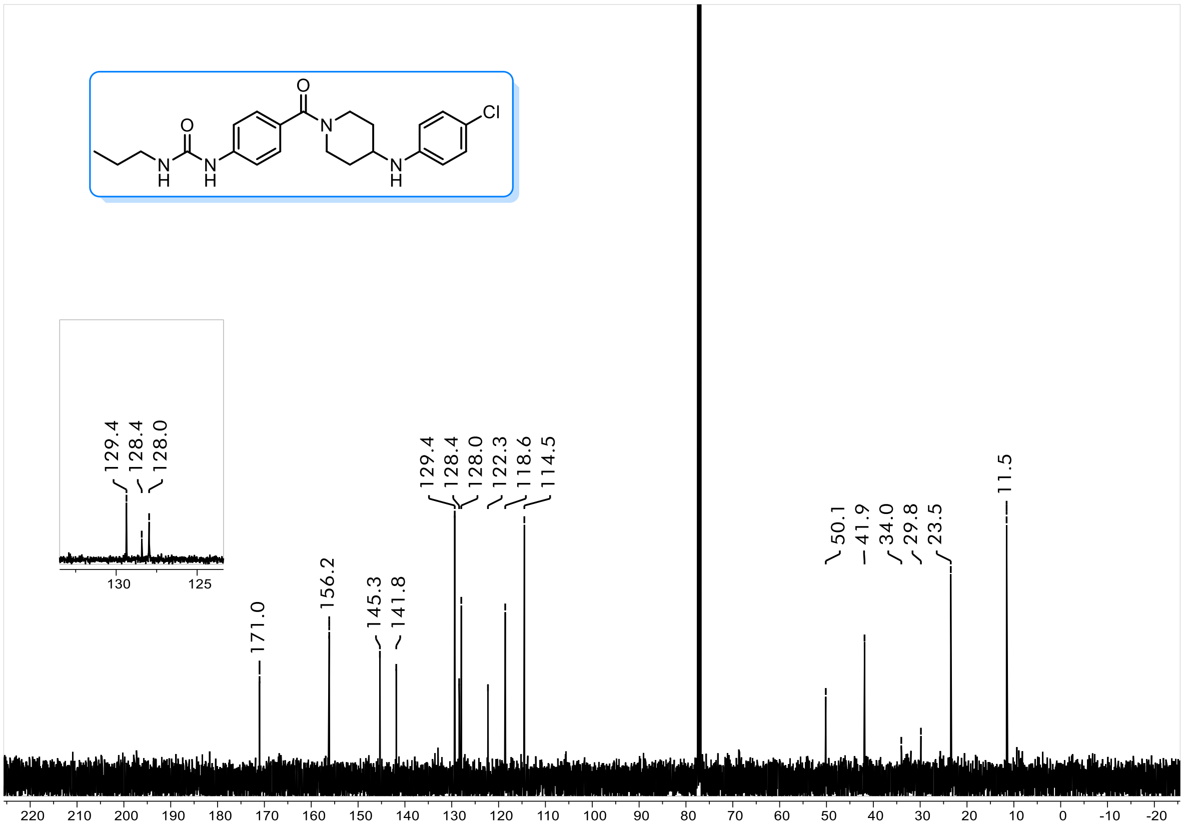

Spectrum 74. ^{13}C-NMR of 1-(4-(4-((4-chlorophenyl)amino)piperidine-1-carbonyl)phenyl)-3-propylurea (3b).

Description:
Ionization Mode:ESI+
History:Determine m/z[Peak Detect[Centroid,30,Area];Correct Base[];Smooth[5]];Correct Base[5.0%];Average(MS[...

Mass Calibration data:Cal_PEG_600
Created:3/11/2024 11:41:07 AM
Created by:AccuTOF

Charge number:1
Element:^{12}C:0 .. 22, ^{1}H:0 .. 55, ^{35}Cl:1 .. 1, ^{14}N:4 .. 4, ^{16}O:2 .. 2

Tolerance:100.00(ppm), 5.00 .. 15.00(mmu)

Unsaturation Number:-1.0 .. 100.0 (Fraction:.5)

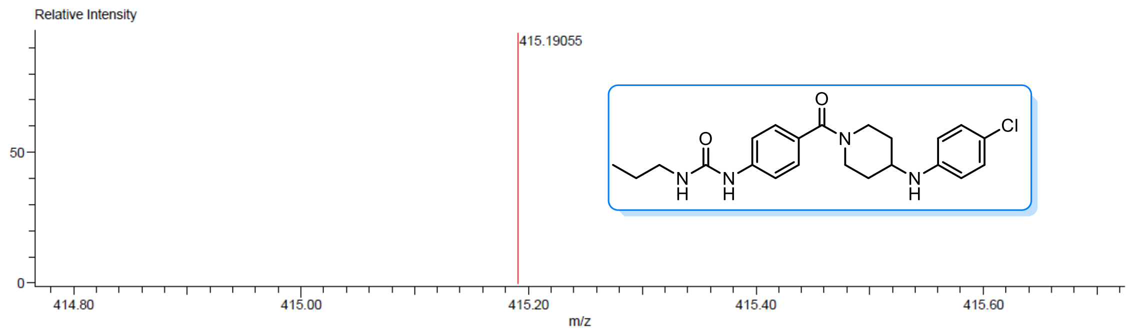

Mass	Intensity	Calc. Mass	Mass Difference (mmu)	Mass Difference (ppm)	Possible Formula	Unsaturation Number
415.19055	60937.91	415.19008	0.47	1.13	$^{12}C_{22}{}^{1}H_{28}{}^{35}Cl_{1}{}^{14}N_{4}{}^{16}O_{2}$	10.5

Spectrum 75. HRMS of 1-(4-(4-((4-chlorophenyl)amino)piperidine-1-carbonyl)phenyl)-3-propylurea (3b).

X-Ray data

Table S1. Crystal data and structure refinement for **1b**

Empirical formula	$C_{18} H_{21} N_3 O_2$	
Formula weight	311.38	
Temperature	150(2) K	
Wavelength	0.71073 Å	
Crystal system	Monoclinic	
Space group	$P2_1/c$	
Unit cell dimensions	a = 9.3039(7) Å	α= 90°.
	b = 9.1436(6) Å	β= 101.586(3)°.
	c = 19.8648(15) Å	γ = 90°.
Volume	1655.5(2) Å^3	
Z	4	
Density (calculated)	1.249 Mg/m^3	
Absorption coefficient	0.083 mm^{-1}	
F(000)	664	
Crystal size	0.321 x 0.270 x 0.186 mm^3	
Theta range for data collection	2.235 to 27.522°.	
Index ranges	-12<=h<=12, -11<=k<=11, -25<=l<=25	
Reflections collected	49793	
Independent reflections	3759 [R(int) = 0.0907]	
Completeness to theta = 25.242°	99.2 %	
Absorption correction	None	
Refinement method	Full-matrix least-squares on F^2	
Data / restraints / parameters	3759 / 2 / 216	
Goodness-of-fit on F^2	1.058	
Final R indices [I>2sigma(I)]	R1 = 0.0462, wR2 = 0.1096	
R indices (all data)	R1 = 0.0533, wR2 = 0.1157	
Largest diff. peak and hole	0.396 and -0.237 e.Å^{-3}	

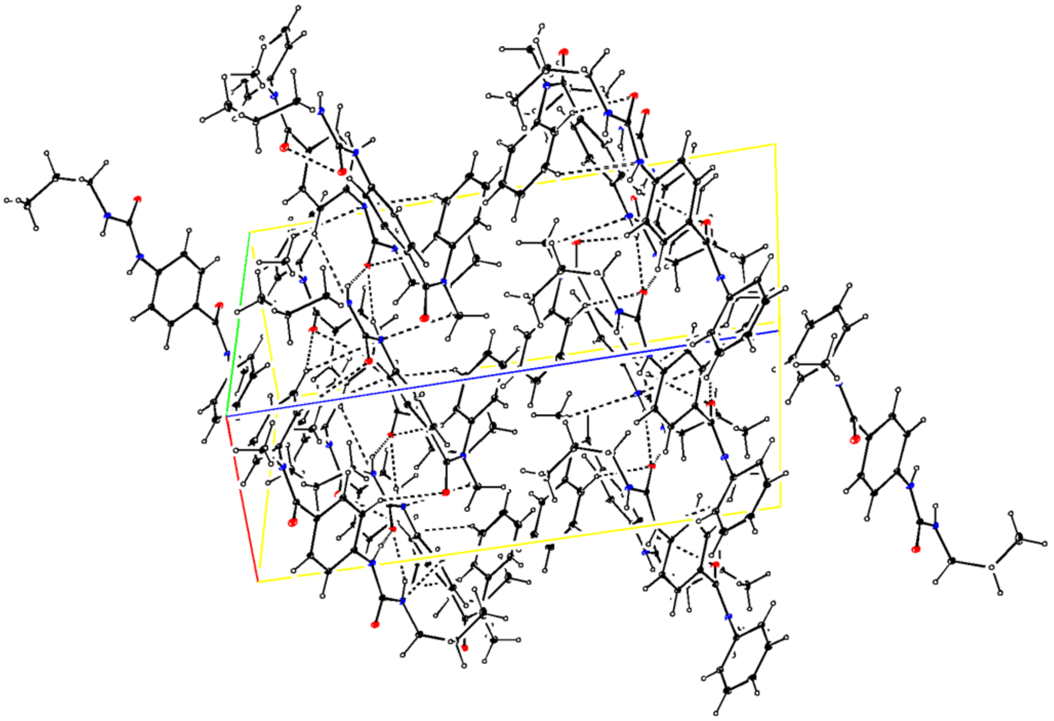

Figure S1.

SWISSADME PREDICTIONS

Table S2. SwissADME predictions.

Compound	logP	Hydrogen donors	Hydrogen aceptors	Rotatable bonds	PSA	Silicos-IT class
**1a**	2.7	2	2	8	61.44	Moderately soluble
**1b**	3.15	2	2	8	61.44	Poorly soluble
**1c**	2.82	2	3	9	70.67	Moderately soluble
**1d**	3.39	2	2	8	61.44	Poorly soluble
**1e**	3.74	2	2	8	61.44	Poorly soluble
**1f**	3.74	2	2	8	61.44	Poorly soluble
**1g**	2.6	2	4	8	79.9	Moderately soluble
**2a**	2.88	2	2	9	61.44	Poorly soluble
**2b**	3.18	2	2	9	61.44	Poorly soluble
**2c**	2.87	2	3	10	70.67	Poorly soluble
**2d**	3.4	2	2	9	61.44	Poorly soluble
**2e**	3.18	2	2	10	61.44	Poorly soluble
**2f**	3.78	2	2	9	61.44	Poorly soluble
**2g**	3.15	2	2	10	61.44	Poorly soluble
**3a**	3	3	2	9	73.47	Poorly soluble
**3b**	3.33	3	2	9	73.47	Poorly soluble
**3c**	2.99	3	3	10	82.7	Poorly soluble
**3d**	3.52	3	2	9	73.47	Poorly soluble
